# Supplementary material for: Long-Term PDE-5A Inhibition Improves Myofilament Function in Left and Right Ventricular Cardiomyocytes through Partially Different Mechanisms in Diabetic Rat Hearts
Source: Antioxidants (Basel). 2021 Nov 6;10(11):1776. doi: 10.3390/antiox10111776 (PMC8615283; doi:10.3390/antiox10111776)
Supplement: Supplementary file 1 [file antioxidants-10-01776-s001.zip › antioxidants-1426499-supplementary.pdf]

Left ventricle_Cardiac Troponin I_original membranes_ Ser-22/23-P

1. Lean
2. Lean+Vard
3. ZDF
4. ZDF+Vard

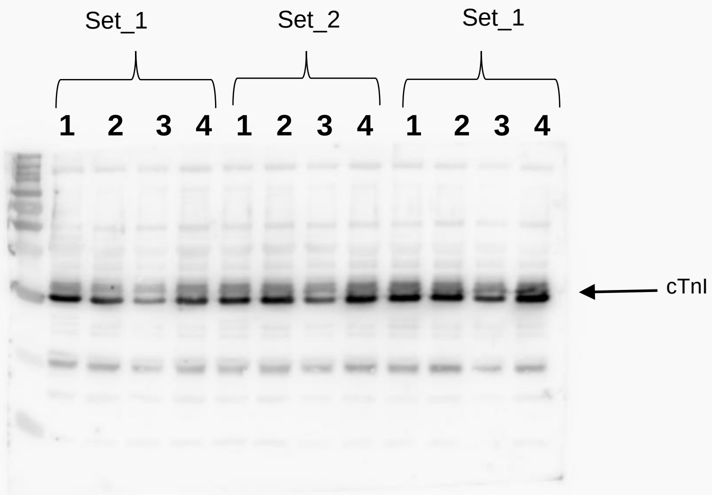

Left ventricle_Cardiac Troponin I_original membranes_ Ser-22/23-P-Blot stain

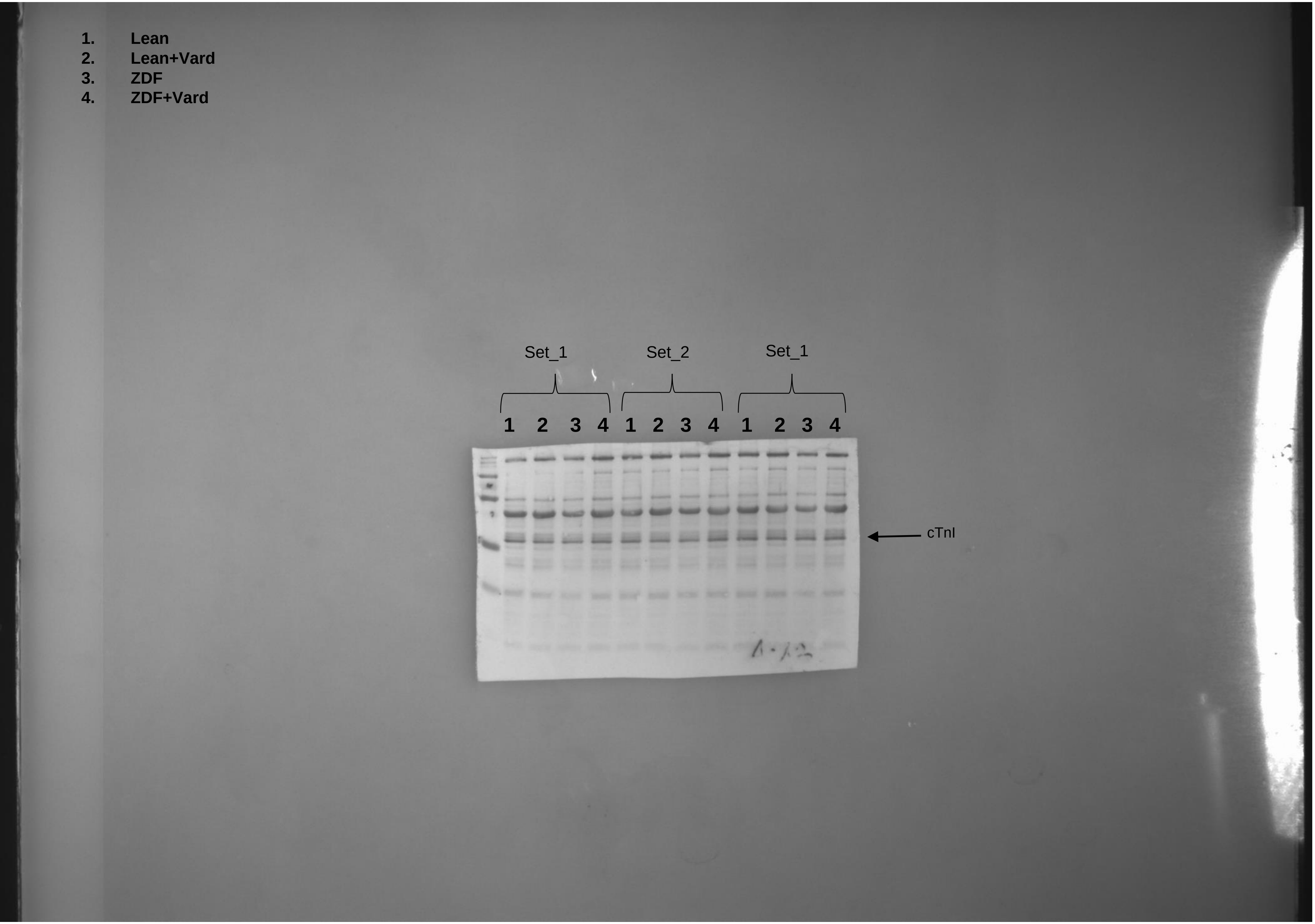

Left ventricle_Cardiac Troponin I_original membranes_ Ser-22/23-P

1. Lean
2. Lean+Vard
3. ZDF
4. ZDF+Vard

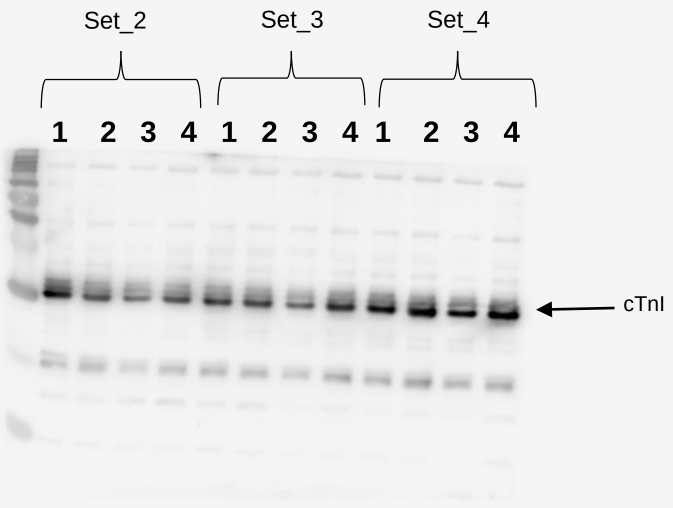

Left ventricle_Cardiac Troponin I_original membranes_ Ser-22/23-P-Blot stain

1. Lean
2. Lean+Vard
3. ZDF
4. ZDF+Vard

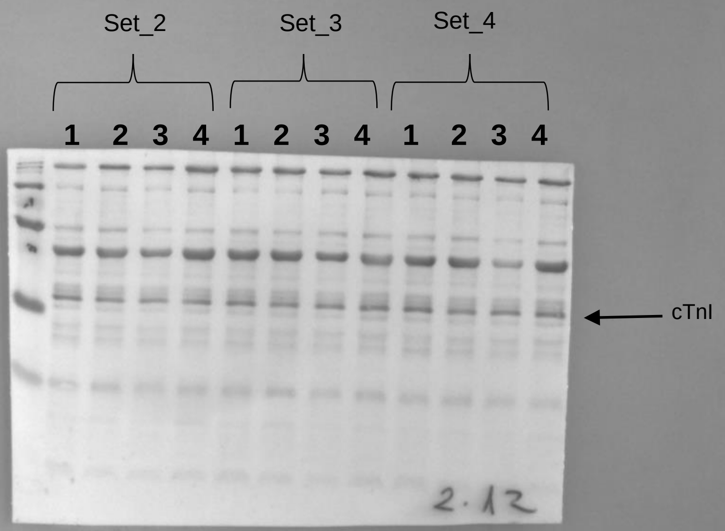

Left ventricle_Cardiac Troponin I_original membranes_ Ser-22/23-P

1. Lean
2. Lean+Vard
3. ZDF
4. ZDF+Vard

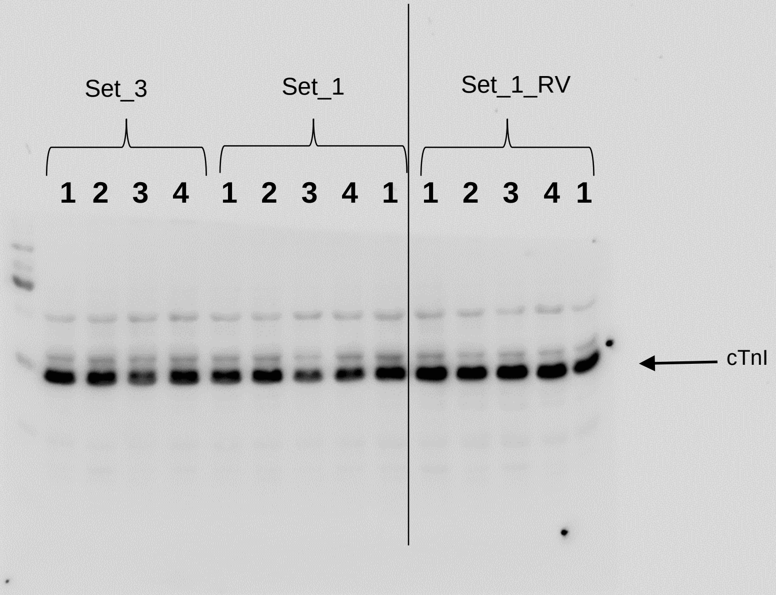

<u>**Left ventricle_Cardiac Troponin I_original membranes_ Ser-22/23-P-Blot stain**</u>

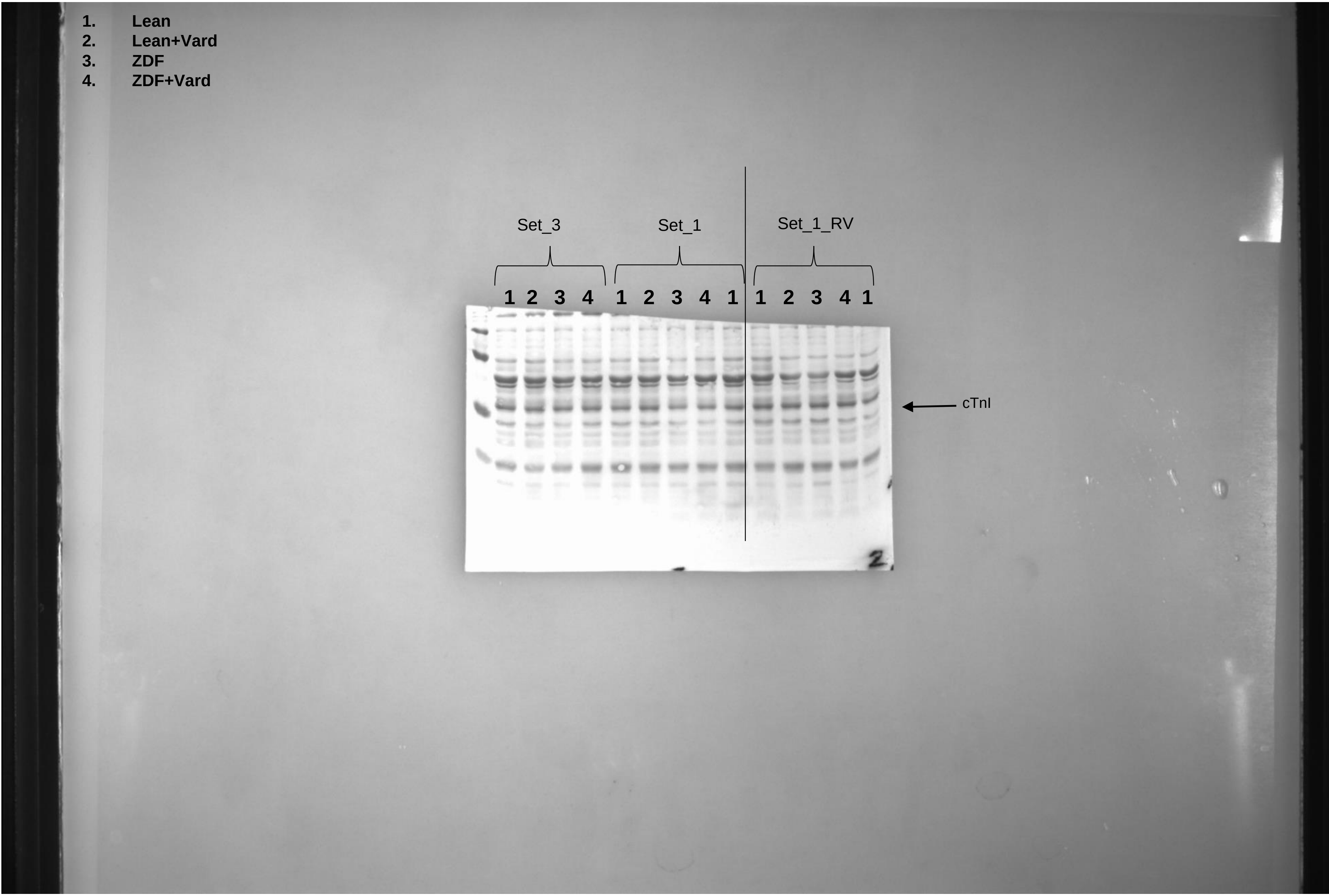

Left ventricle_Cardiac Troponin I_original membranes_ Ser-22/23-P

1. Lean
2. Lean+Vard
3. ZDF
4. ZDF+Vard

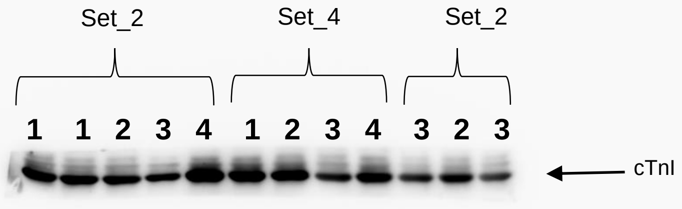

Left ventricle_Cardiac Troponin I_original membranes_ Ser-22/23-P-Blot stain

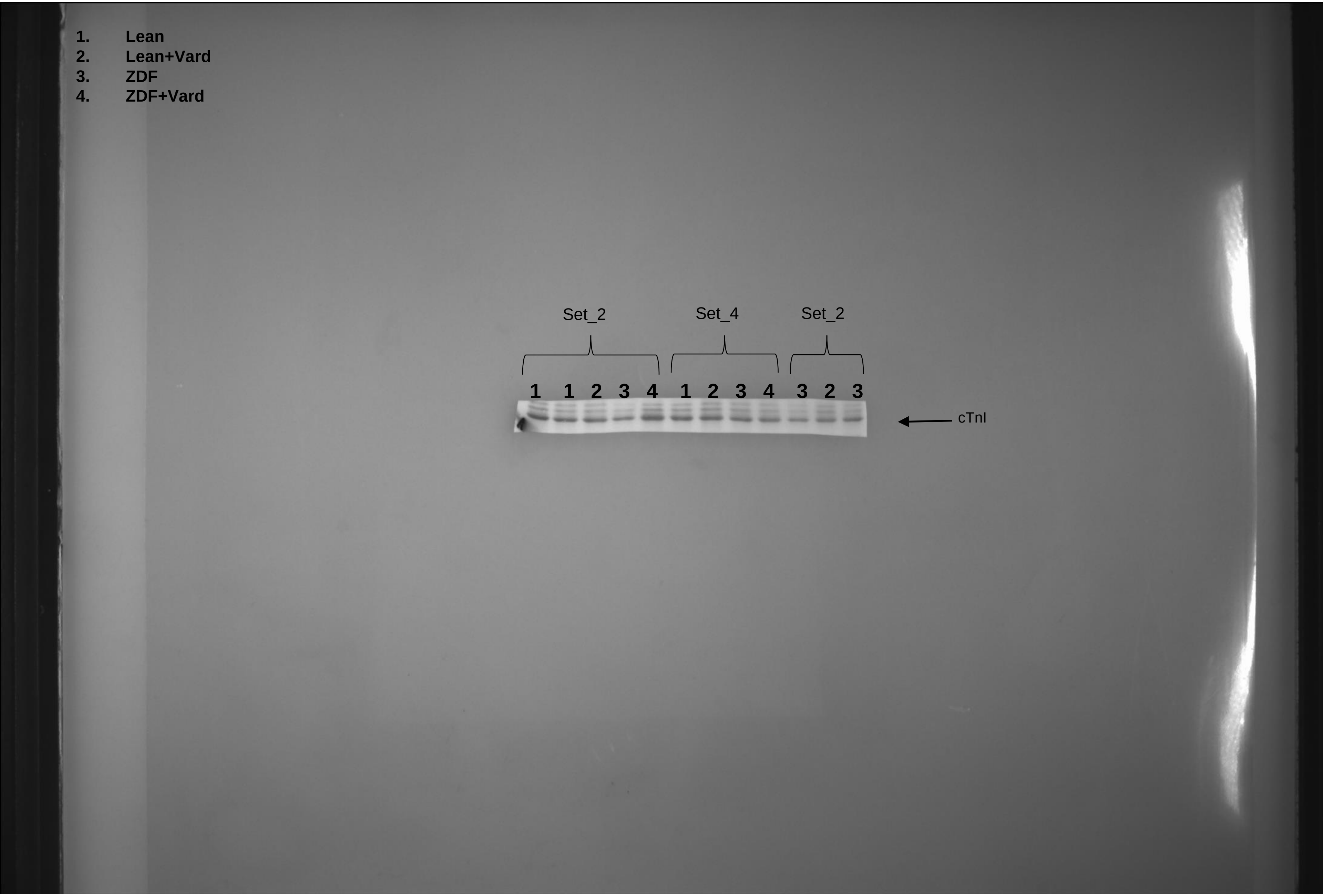

Left ventricle_Cardiac Troponin I_original membranes_ Ser-43-P

1. Lean
2. Lean+Vard
3. ZDF
4. ZDF+Vard

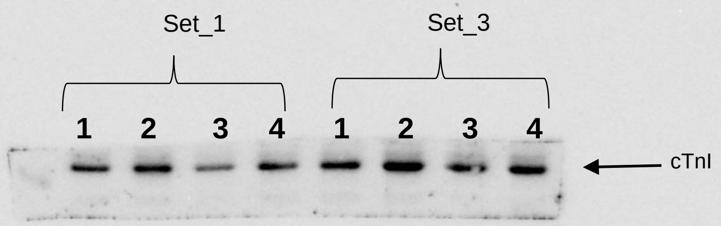

Left ventricle_Cardiac Troponin I_original membranes_ Ser-43-P-Blot stain

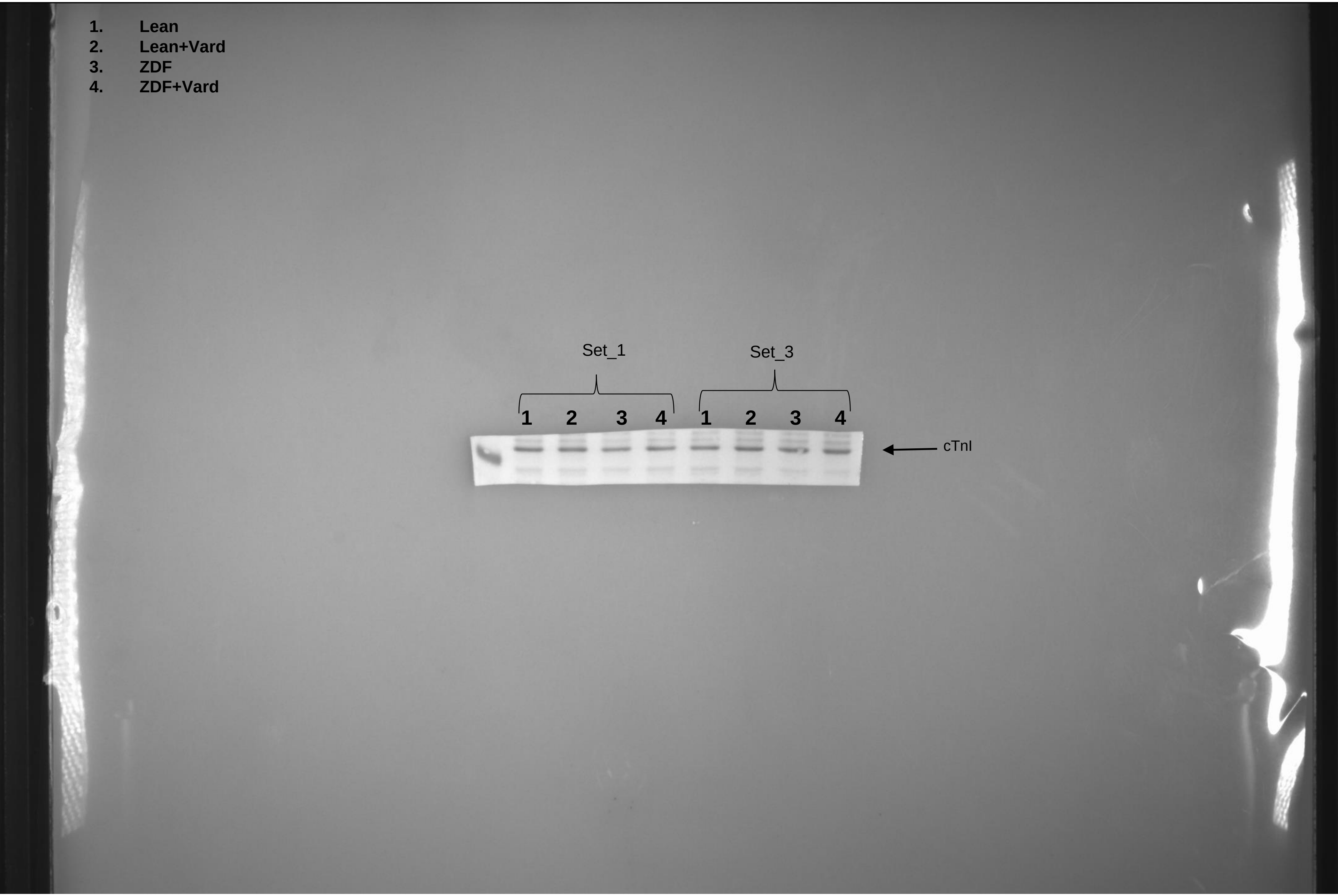

Left ventricle_Cardiac Troponin I_original membranes_ Ser-43-P

1. Lean
2. Lean+Vard
3. ZDF
4. ZDF+Vard

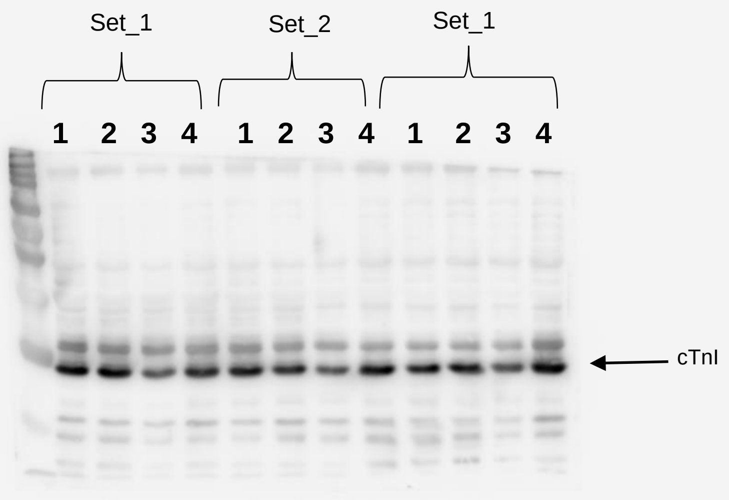

Left ventricle_Cardiac Troponin I_original membranes_ Ser-43-P-Blot stain

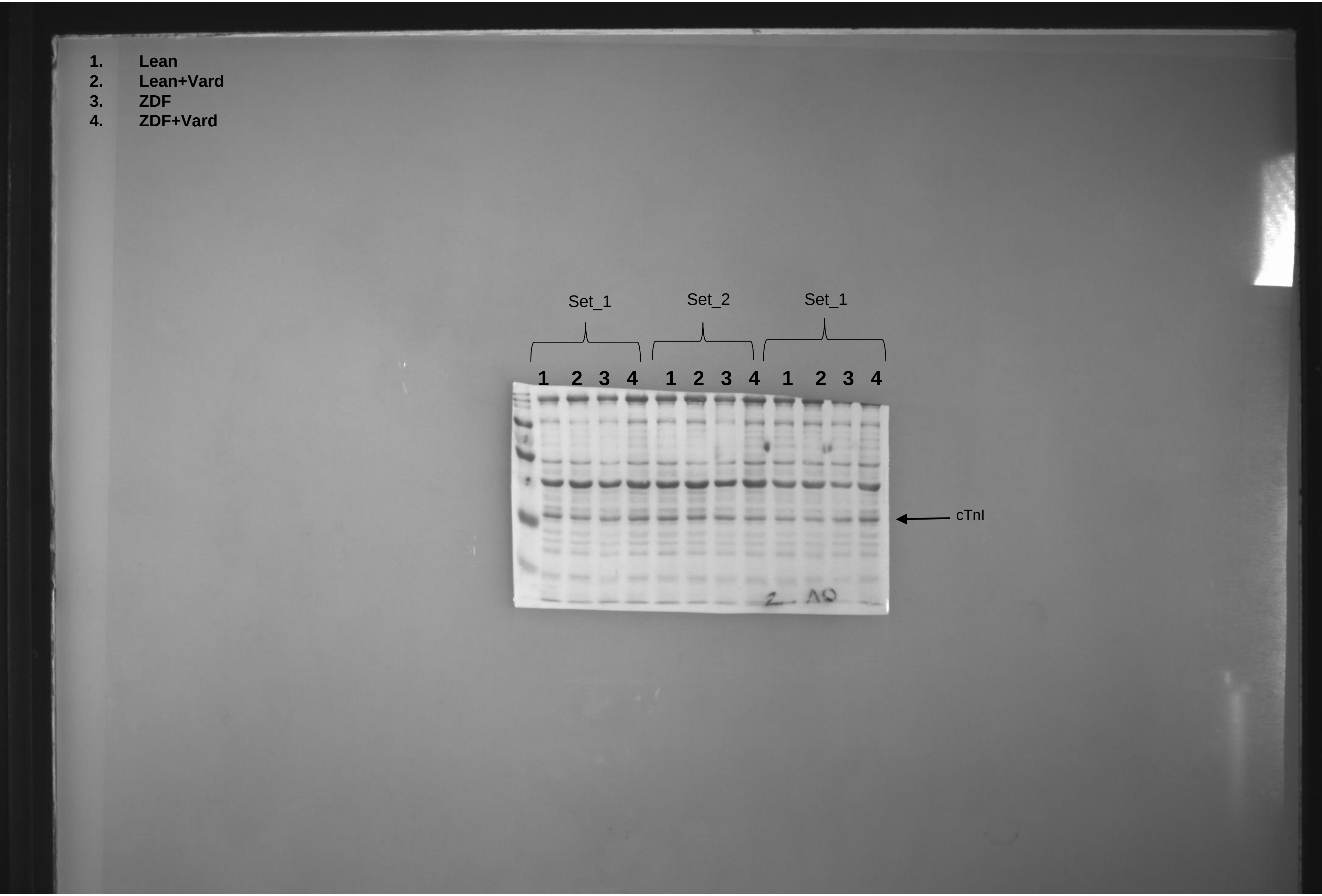

Left ventricle_Cardiac Troponin I_original membranes_ Ser-43-P

1. Lean
2. Lean+Vard
3. ZDF
4. ZDF+Vard

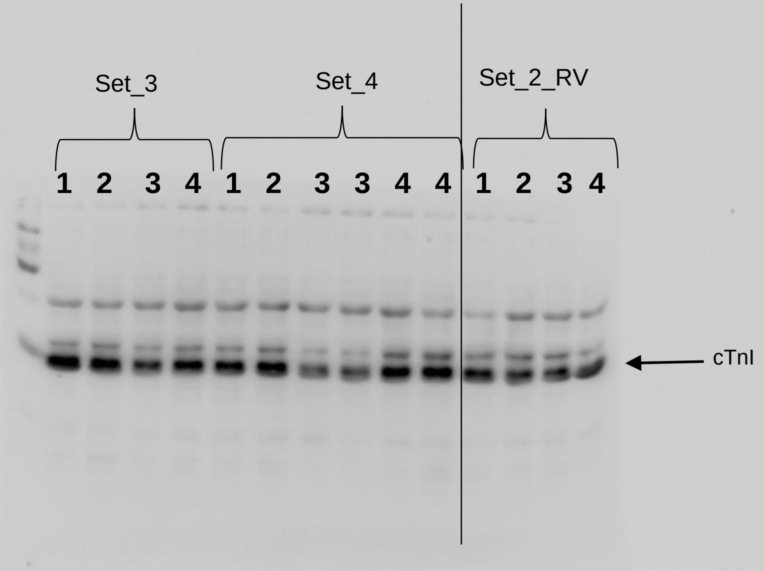

Left ventricle_Cardiac Troponin I_original membranes_ Ser-43-P-Blot stain

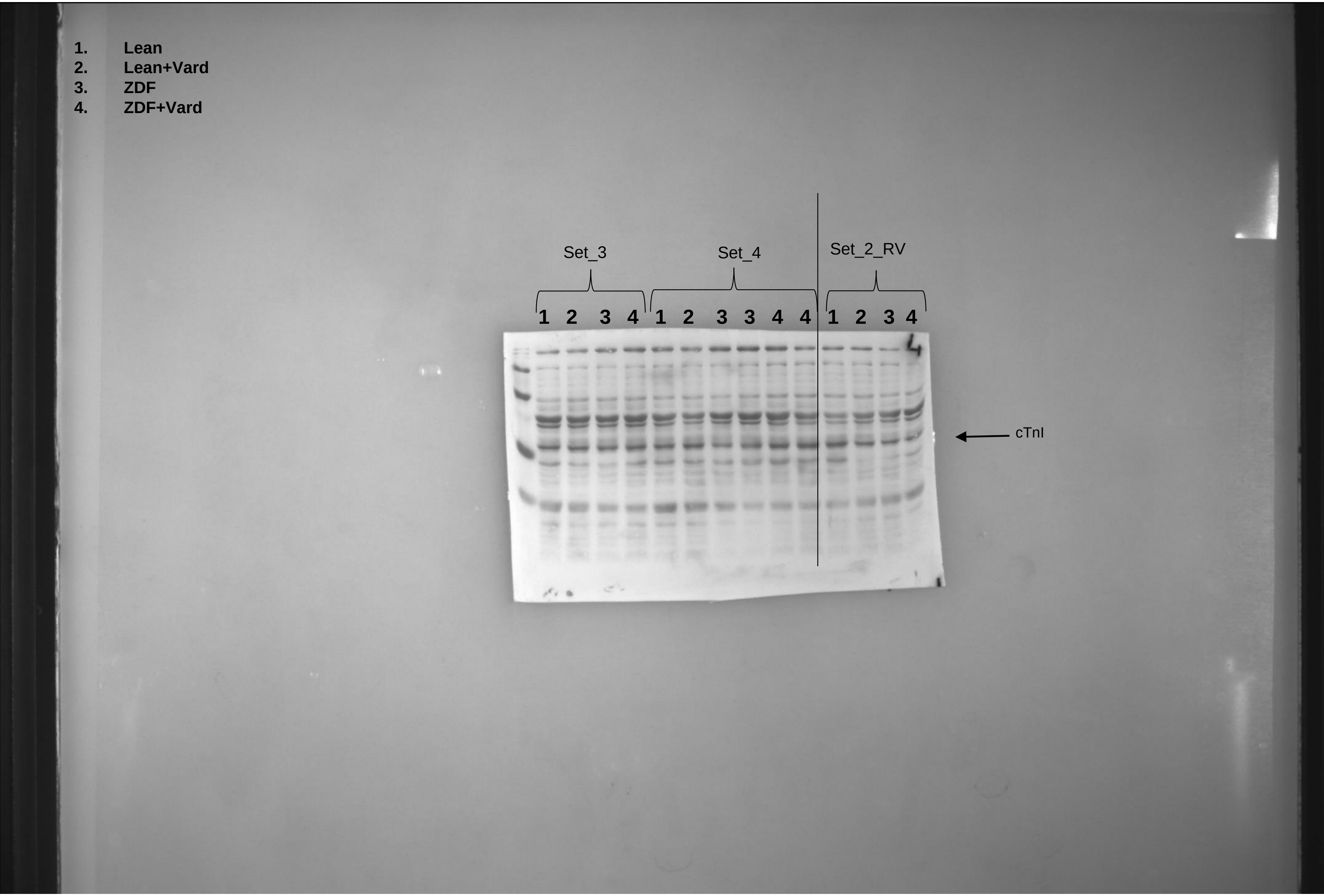

Left ventricle_Cardiac Troponin I_original membranes_ Ser-43-P

1. Lean
2. Lean+Vard
3. ZDF
4. ZDF+Vard

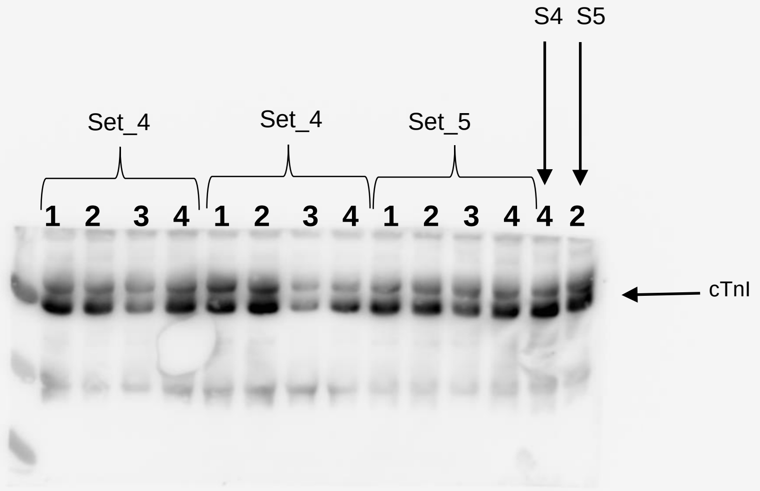

Left ventricle_Cardiac Troponin I_original membranes_ Ser-43-P-Blot stain

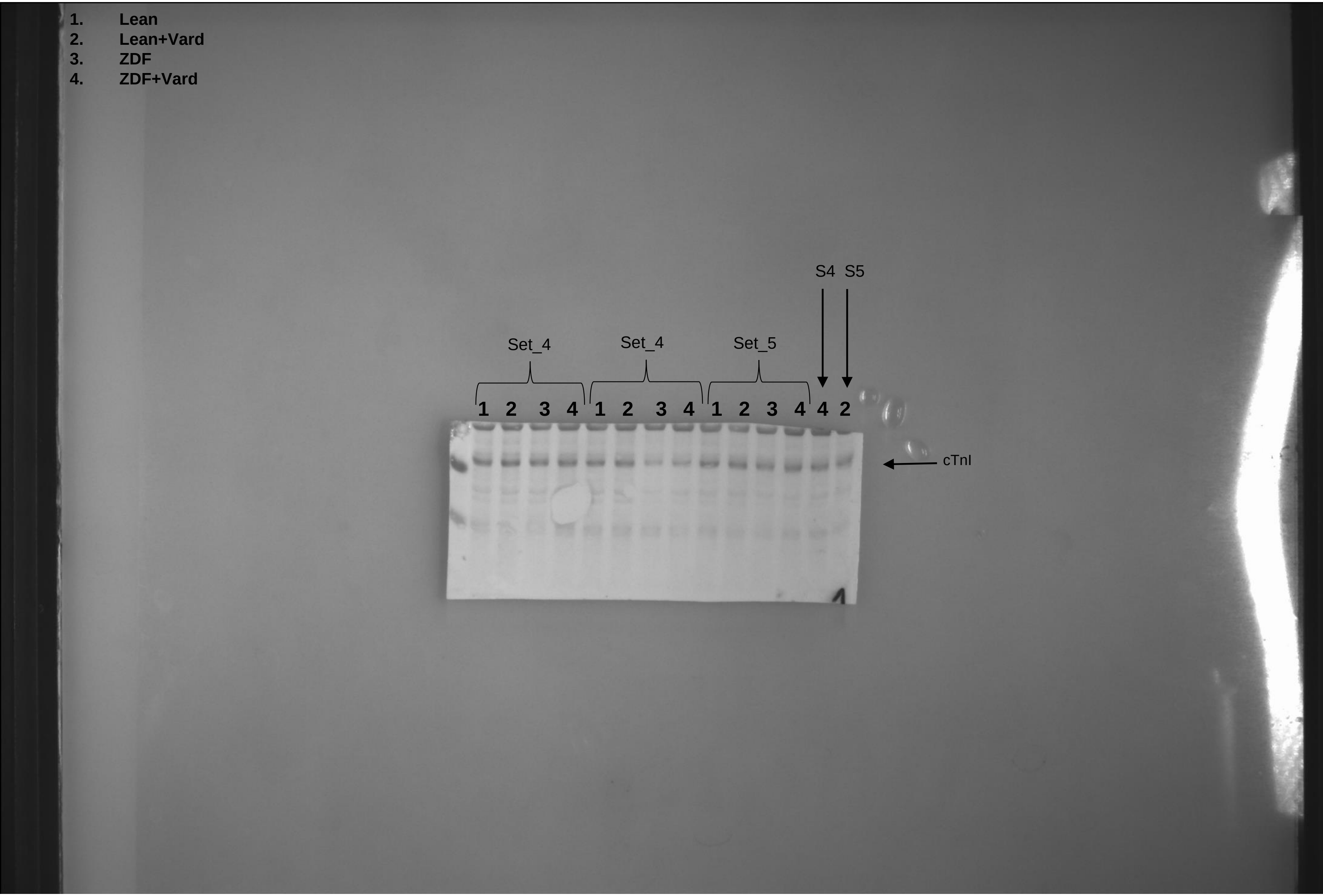

Left ventricle_Cardiac Troponin I_original membranes_ Ser-43-P

1. Lean
2. Lean+Vard
3. ZDF
4. ZDF+Vard

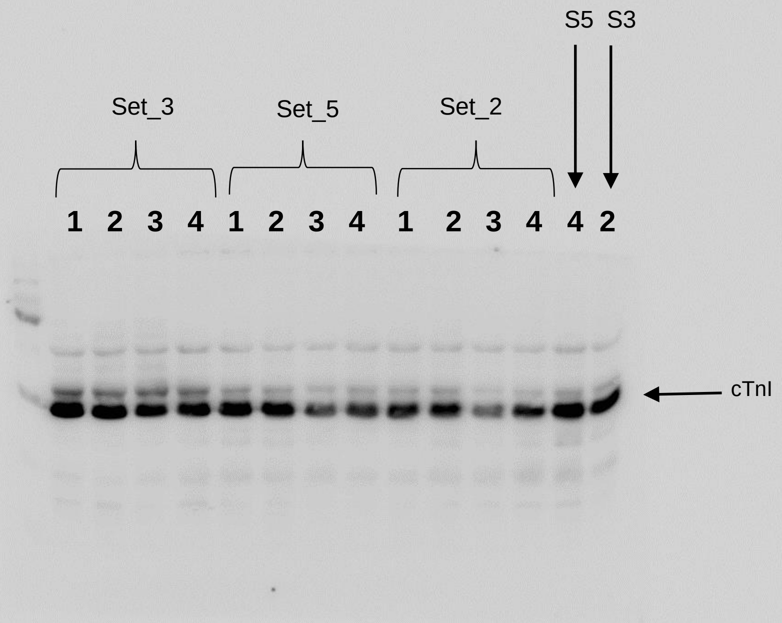

Left ventricle_Cardiac Troponin I_original membranes_ Ser-43-P-Blot stain

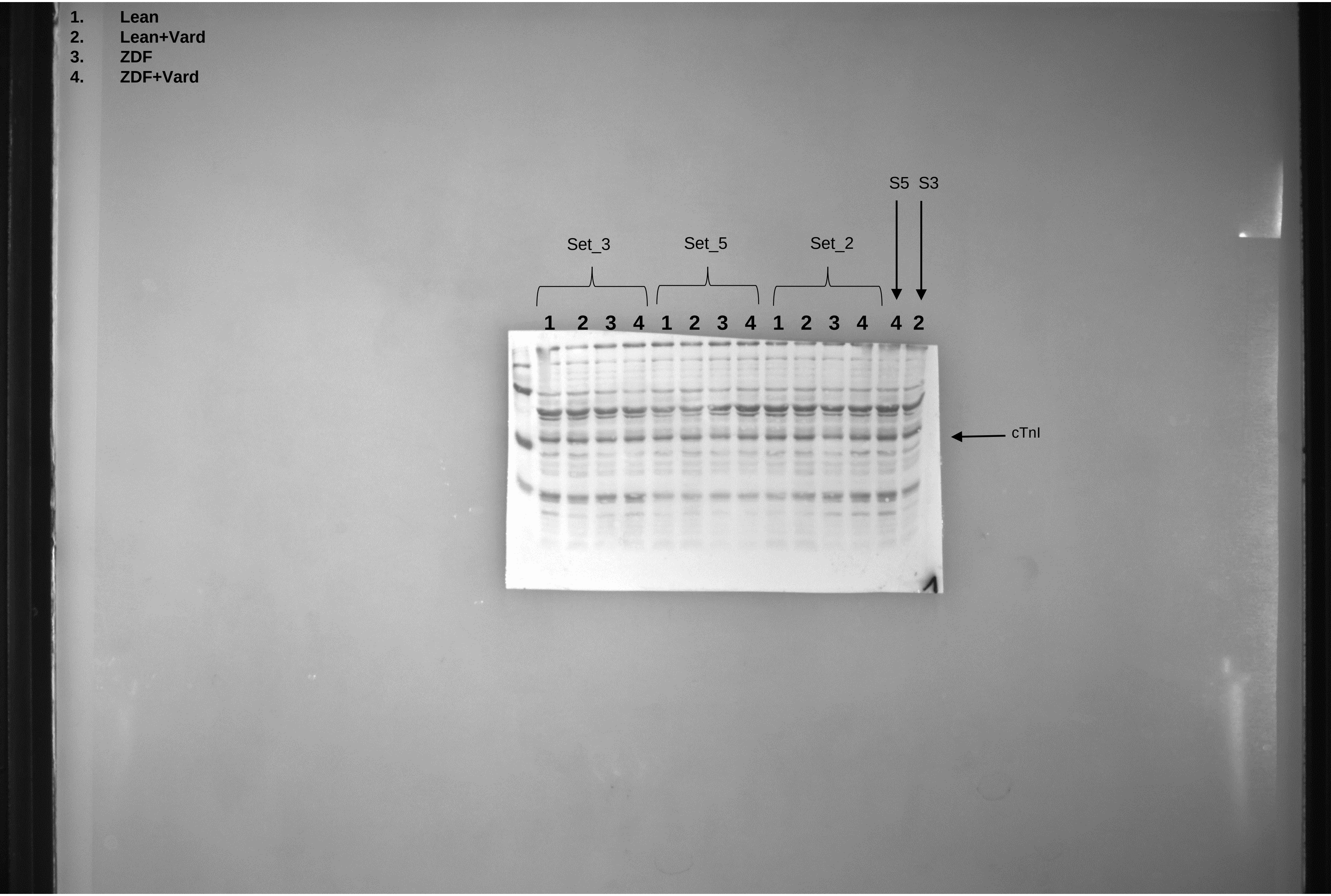

Left ventricle_Cardiac Troponin I_original membranes_ Thr-144-P

1. Lean
2. Lean+Vard
3. ZDF
4. ZDF+Vard

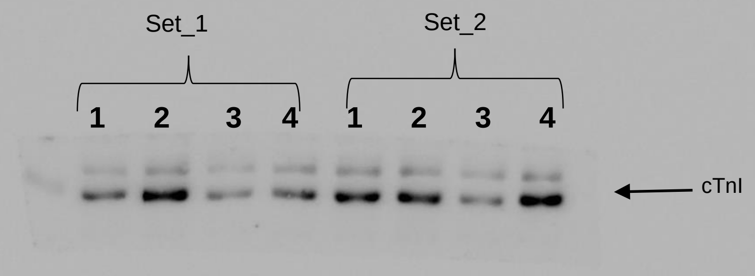

Left ventricle_Cardiac Troponin I_original membranes_ Thr-144-P-Blot stain

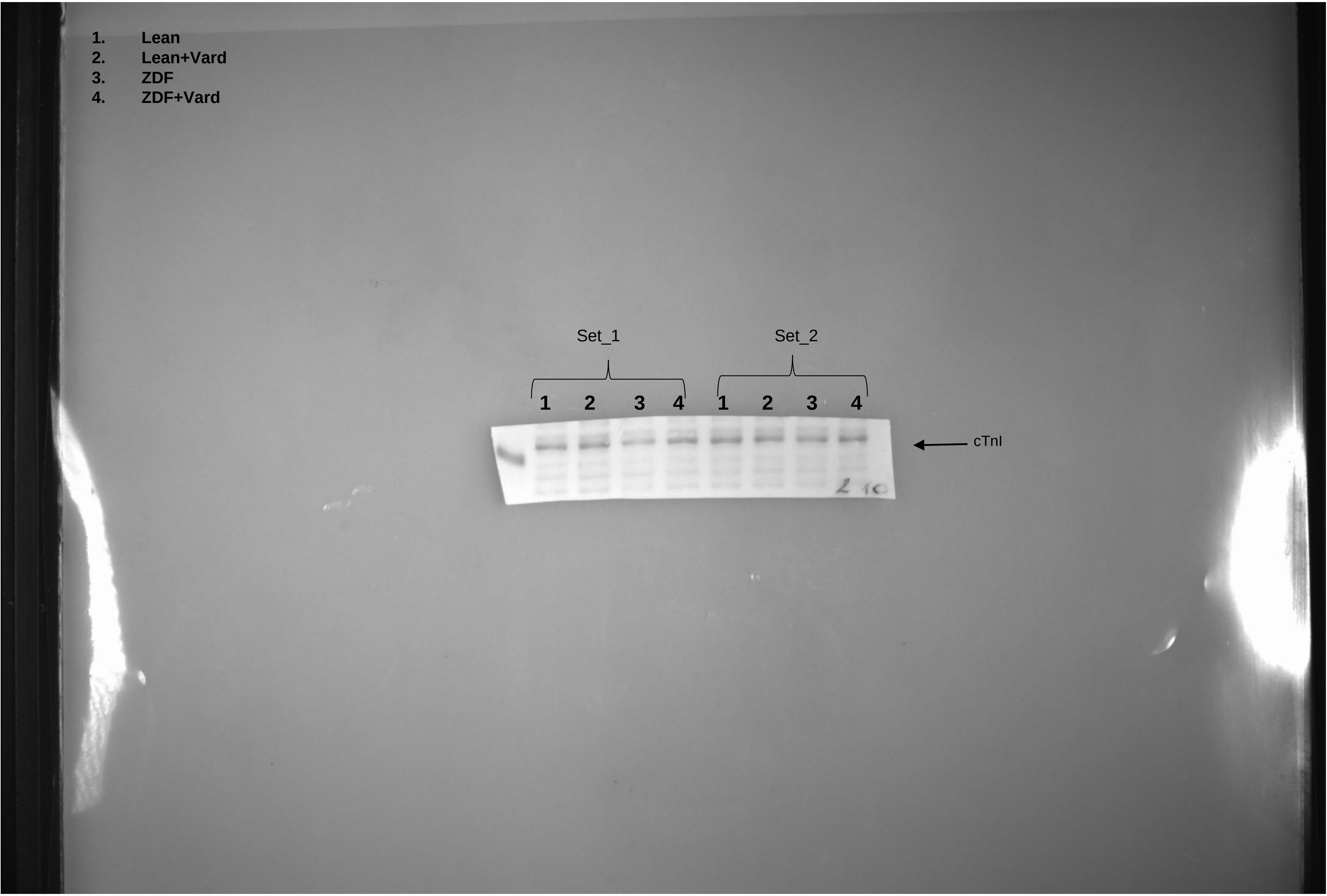

Left ventricle_Cardiac Troponin I_original membranes_ Thr-144-P

1. Lean
2. Lean+Vard
3. ZDF
4. ZDF+Vard

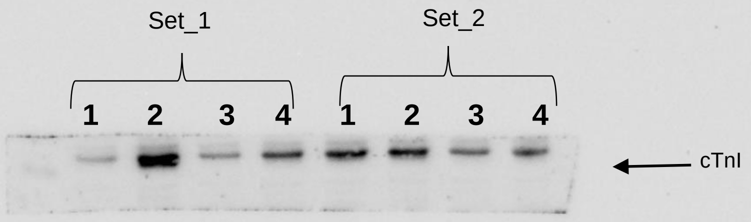

Left ventricle_Cardiac Troponin I_original membranes_ Thr-144-P-Blot stain

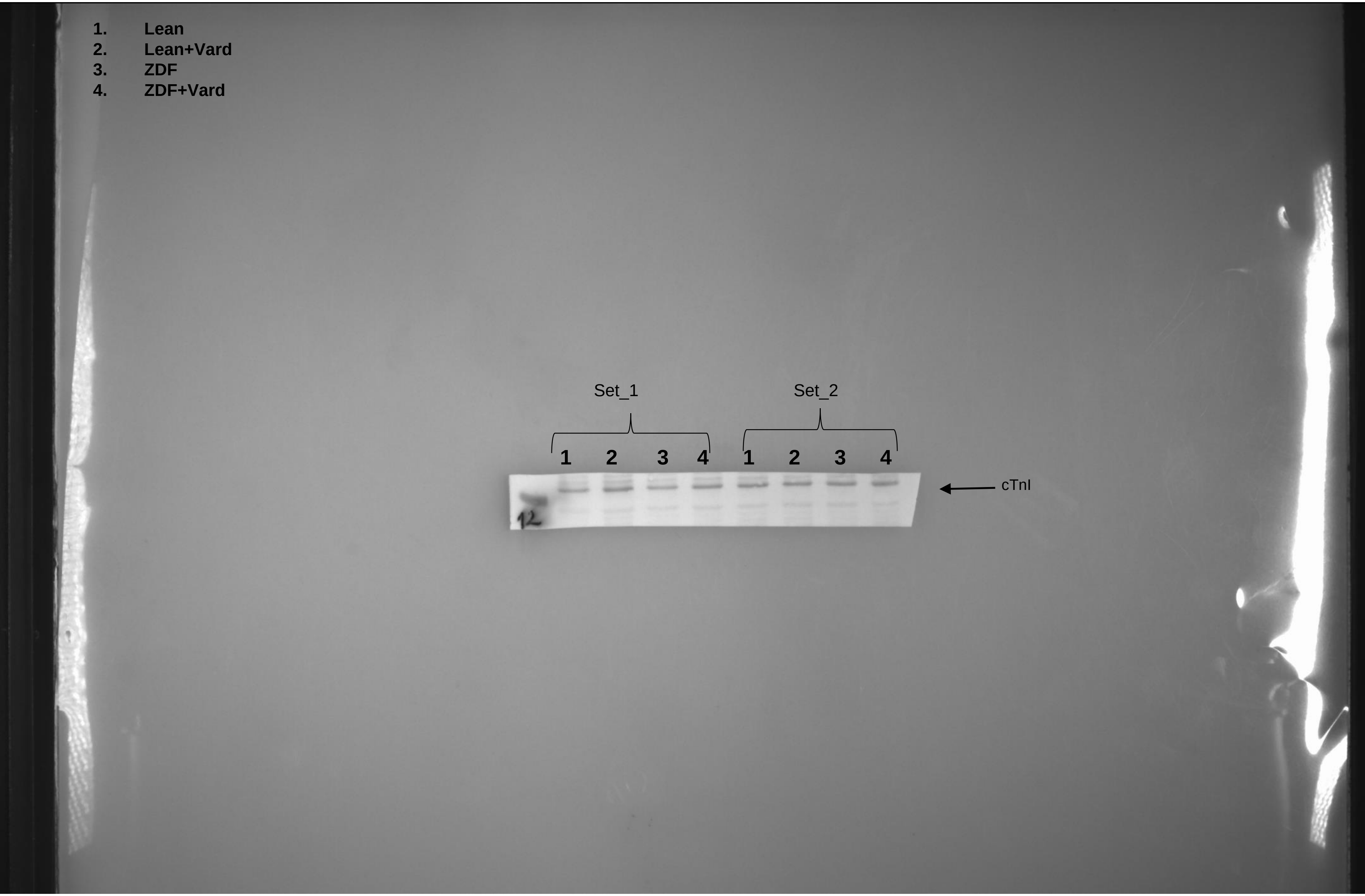

Left ventricle_Cardiac Troponin I_original membranes_ Thr-144-P

1. Lean
2. Lean+Vard
3. ZDF
4. ZDF+Vard

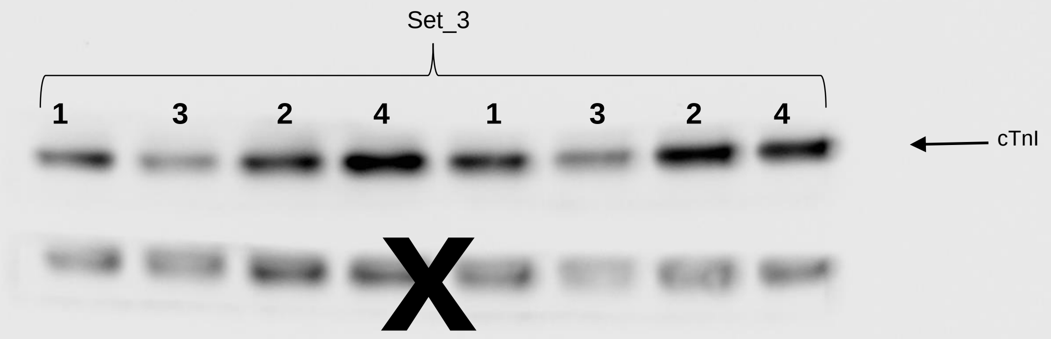

Left ventricle_Cardiac Troponin I_original membranes_ Thr-144-P-Blot stain

1. Lean
2. Lean+Vard
3. ZDF
4. ZDF+Vard

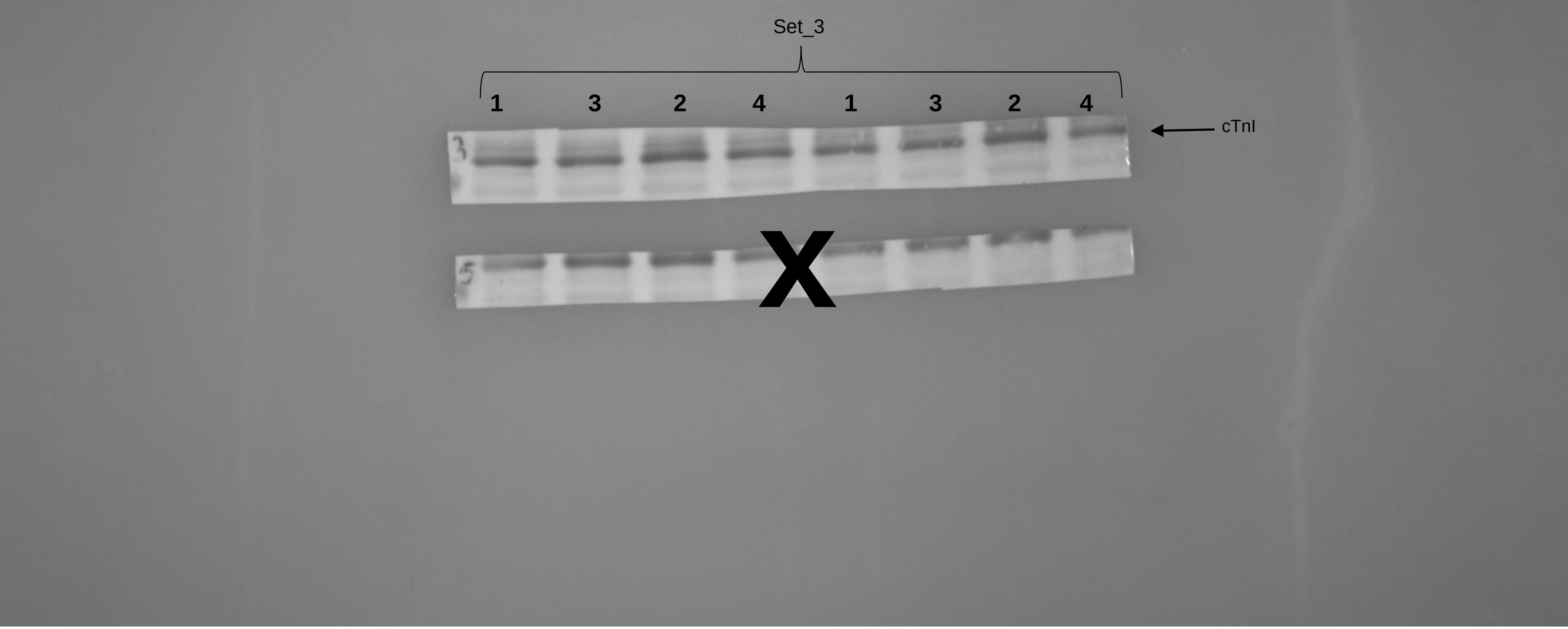

Left ventricle_Cardiac Troponin I_original membranes_ Thr-144-P

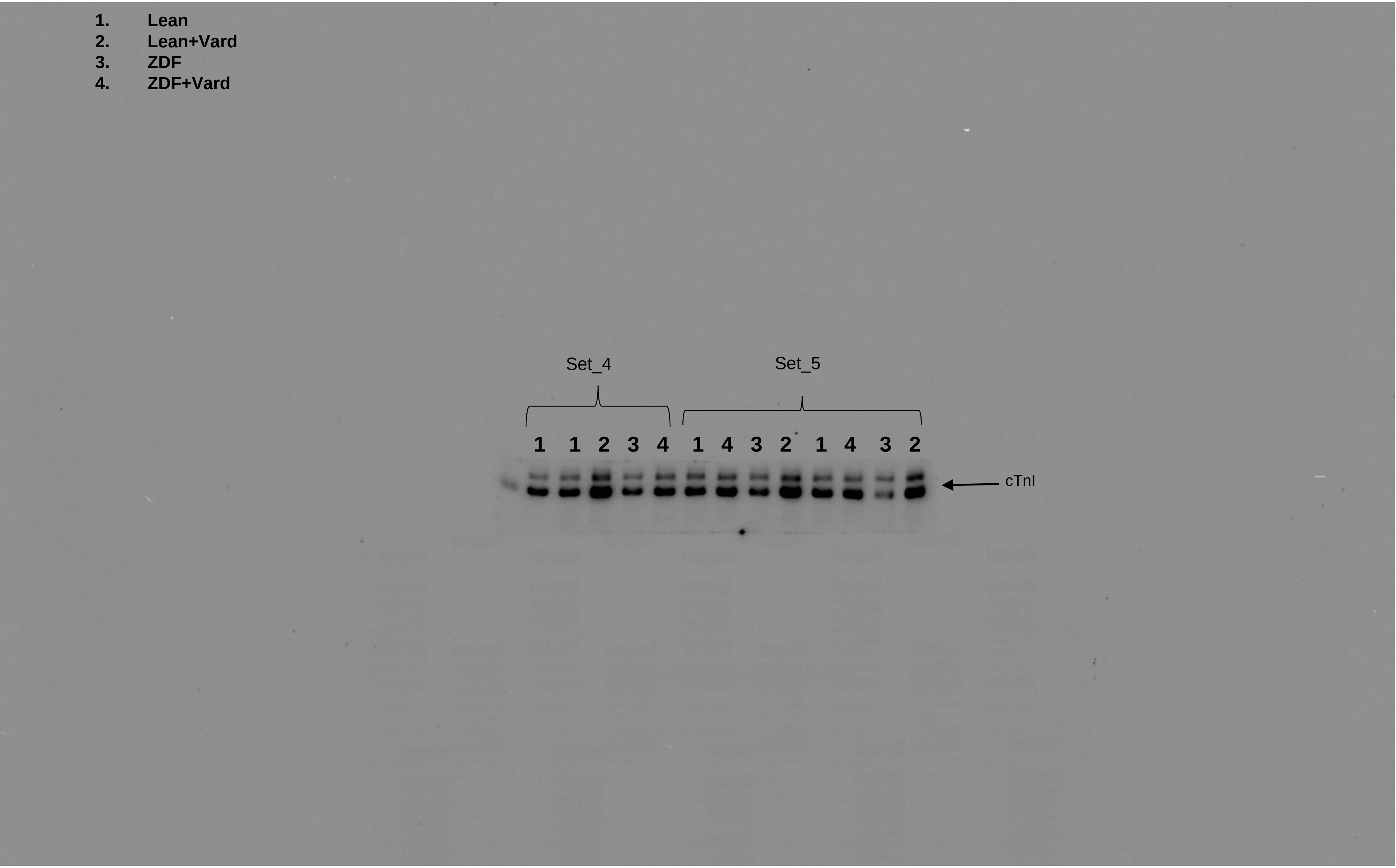

Left ventricle_Cardiac Troponin I_original membranes_ Thr-144-P-Blot stain

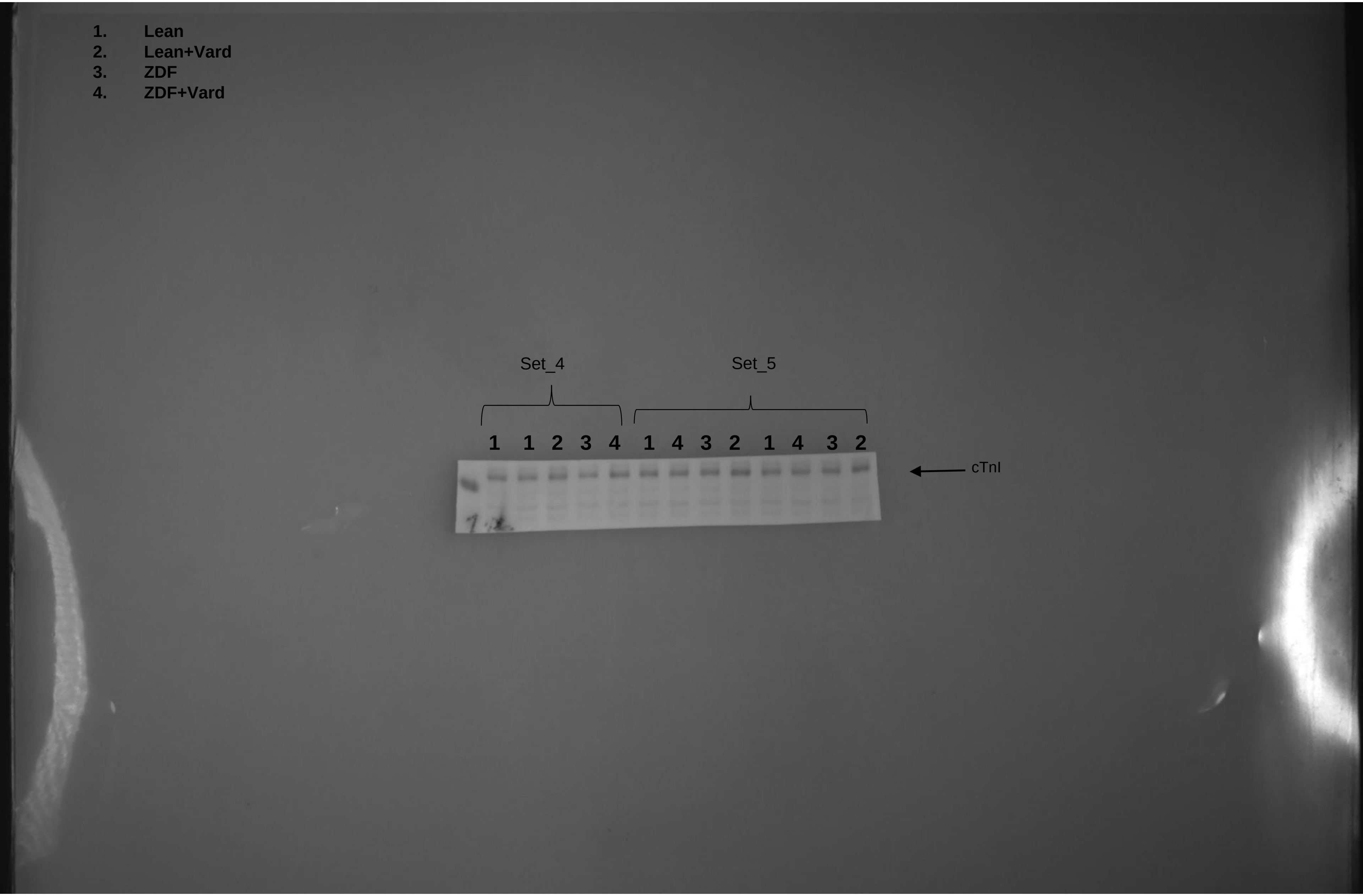

Left ventricle_Cardiac Troponin I_original membranes_ Thr-144-P

1. Lean
2. Lean+Vard
3. ZDF
4. ZDF+Vard

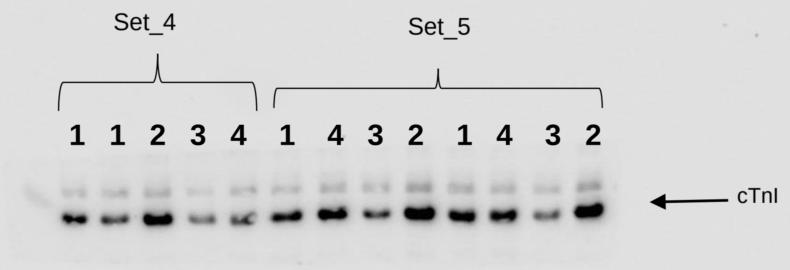

Left ventricle_Cardiac Troponin I_original membranes_ Thr-144-Blot stain

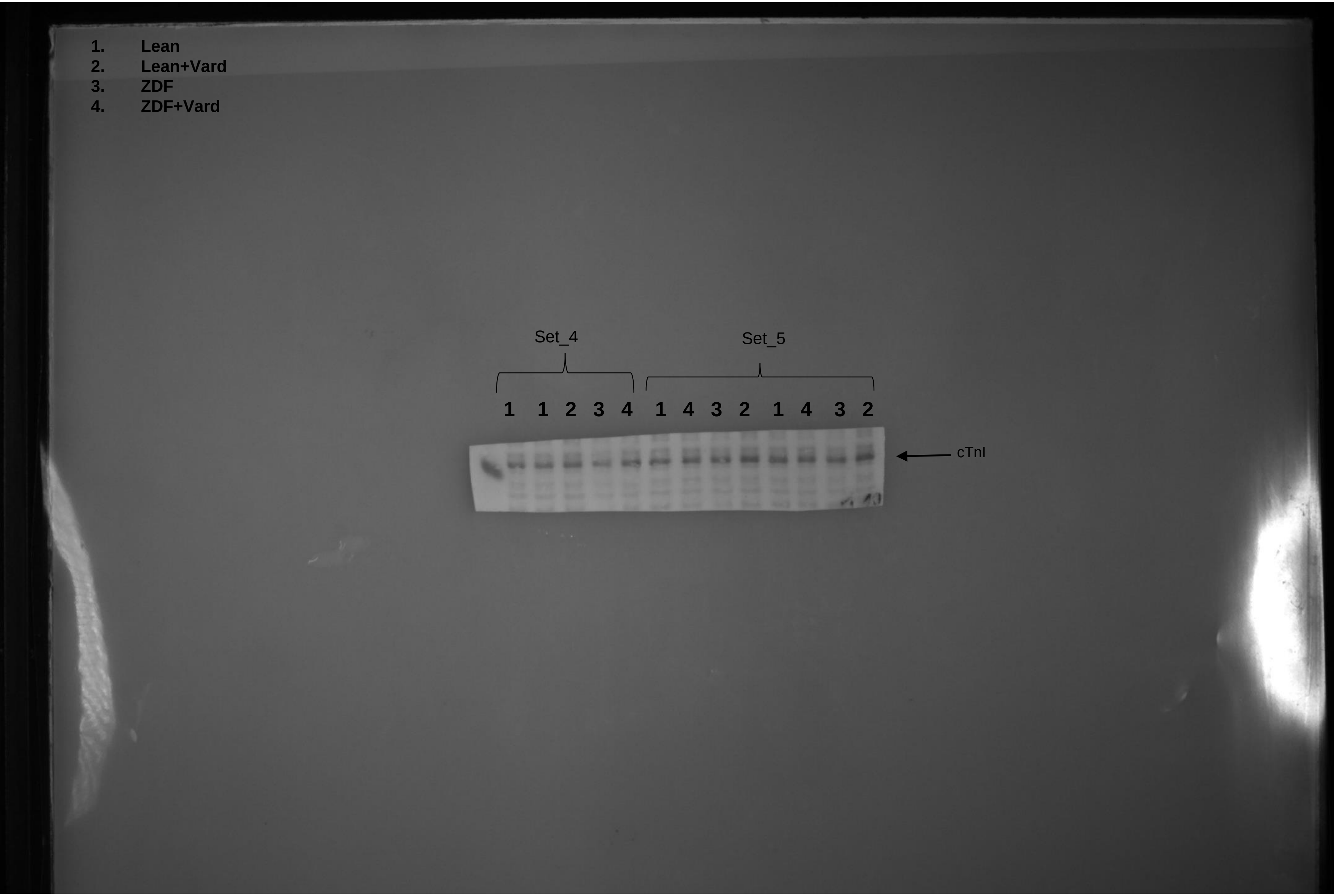

Left ventricle_Cardiac myosin binding protein C_original membranes_ Ser-282-P

1. Lean
2. Lean+Vard
3. ZDF
4. ZDF+Vard

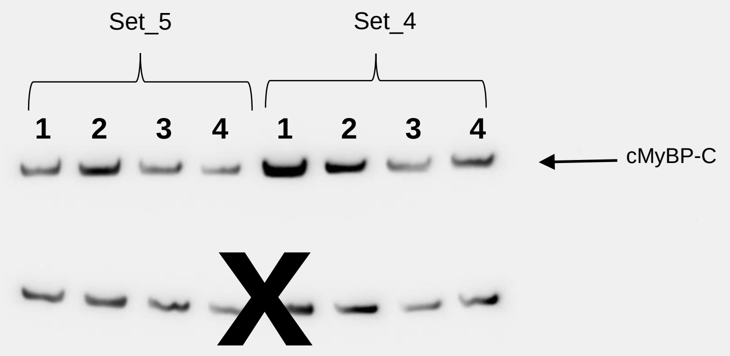

Left ventricle_Cardiac myosin binding protein C_original membranes_ Ser-282-P-Blot stain

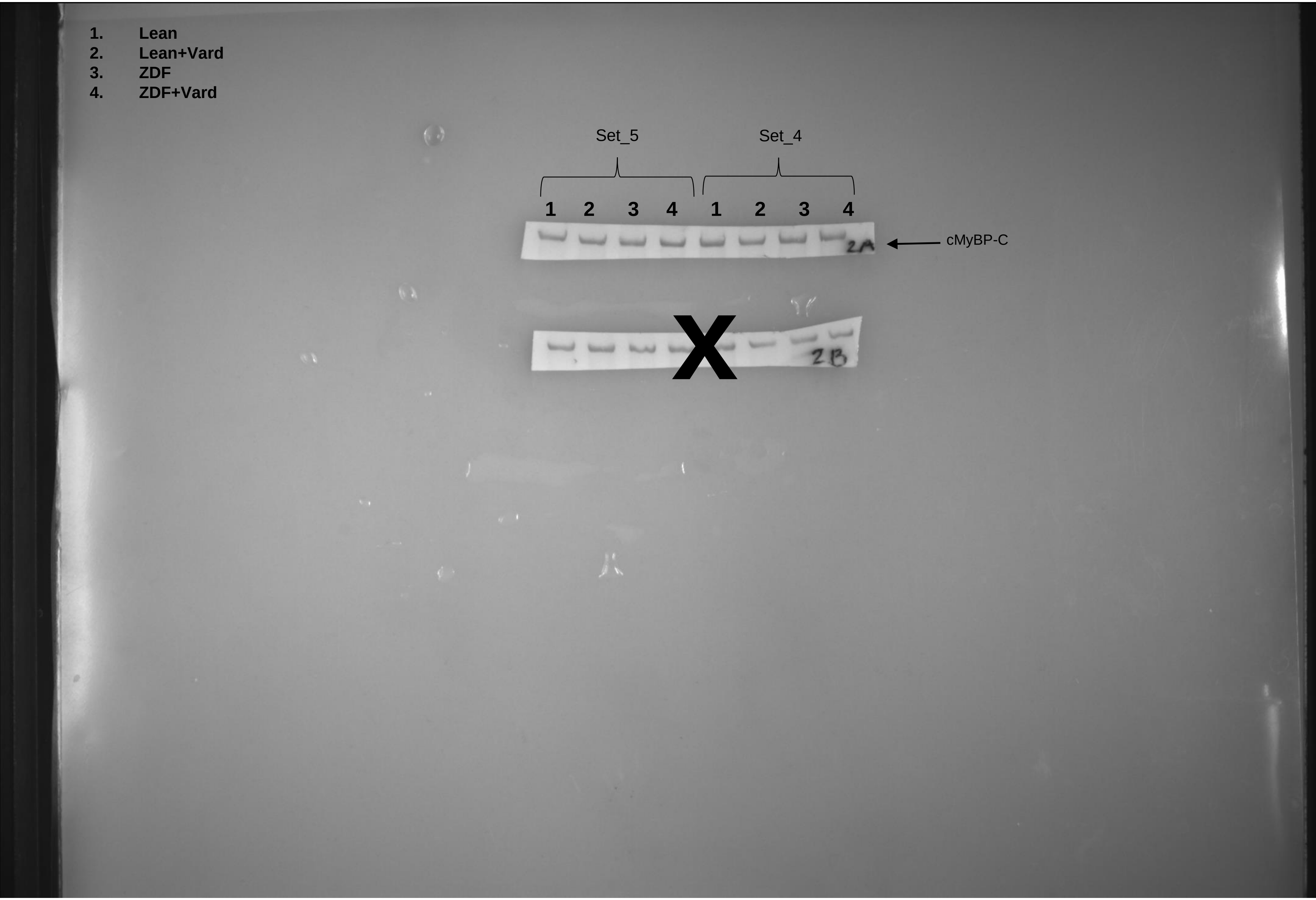

Left ventricle_Cardiac myosin binding protein C_original membranes_ Ser-282-P

1. Lean
2. Lean+Vard
3. ZDF
4. ZDF+Vard

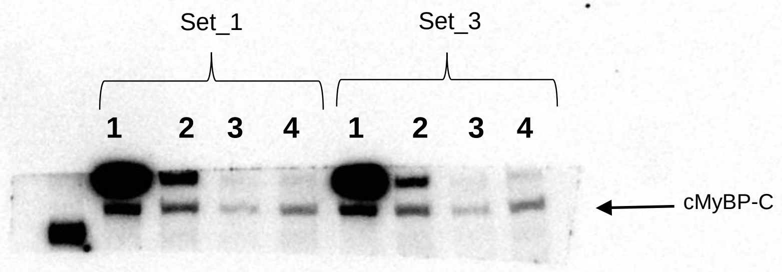

Left ventricle_Cardiac myosin binding protein C_original membranes_ Ser-282-P-Blot stain

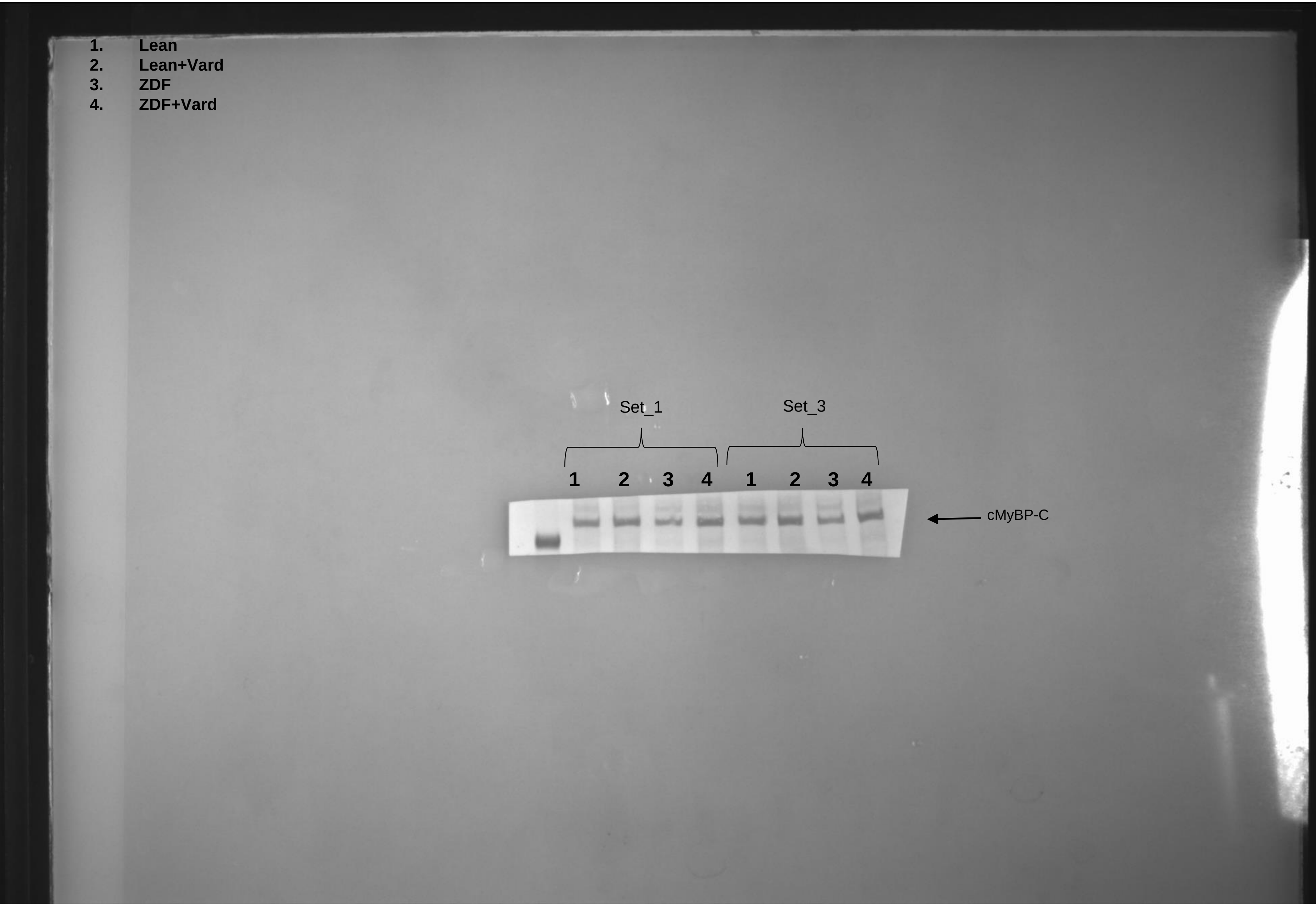

Left ventricle_Cardiac myosin binding protein C_original membranes_ Ser-282-P

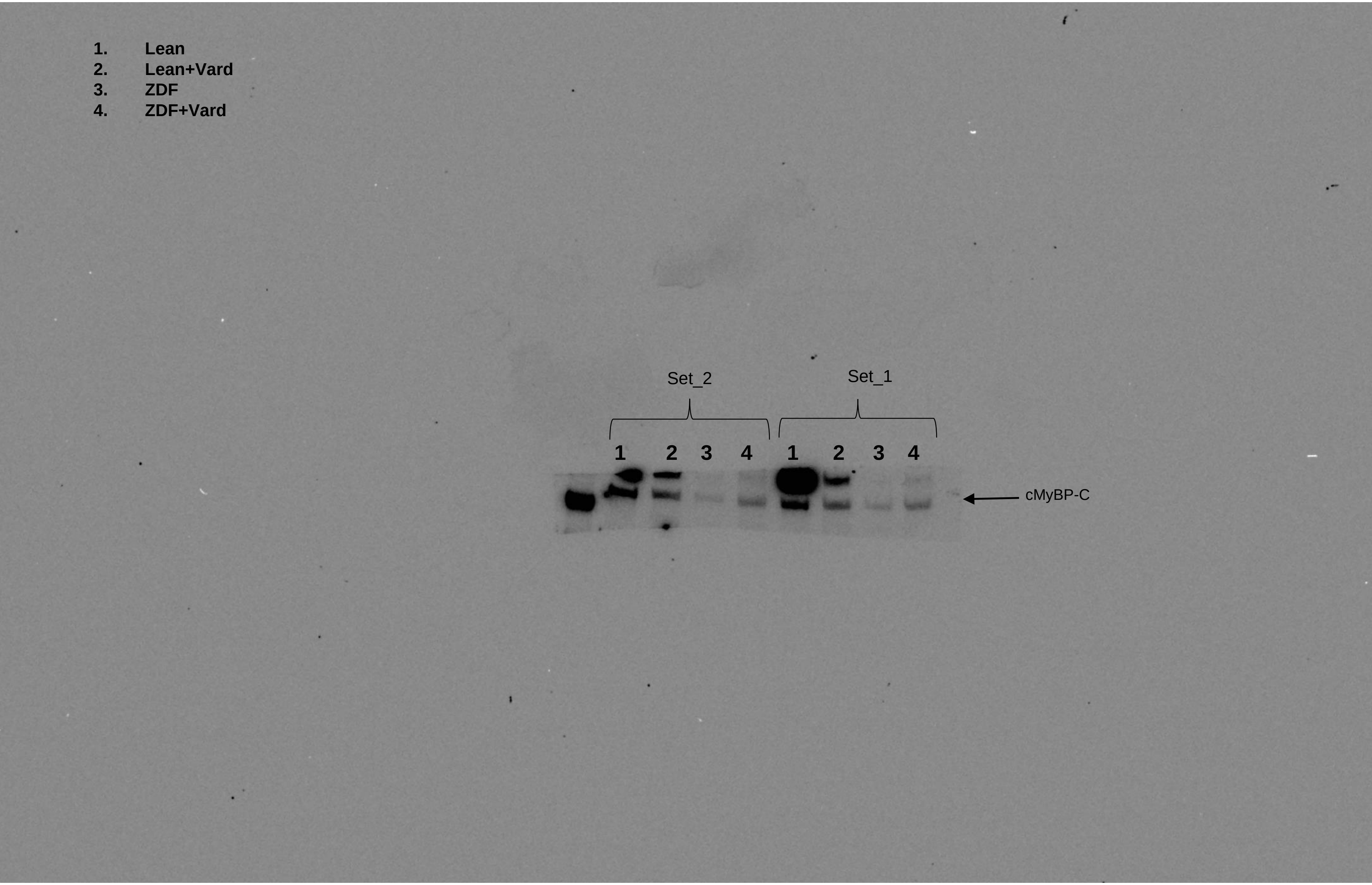

Left ventricle_Cardiac myosin binding protein C_original membranes_ Ser-282-P-Blot stain

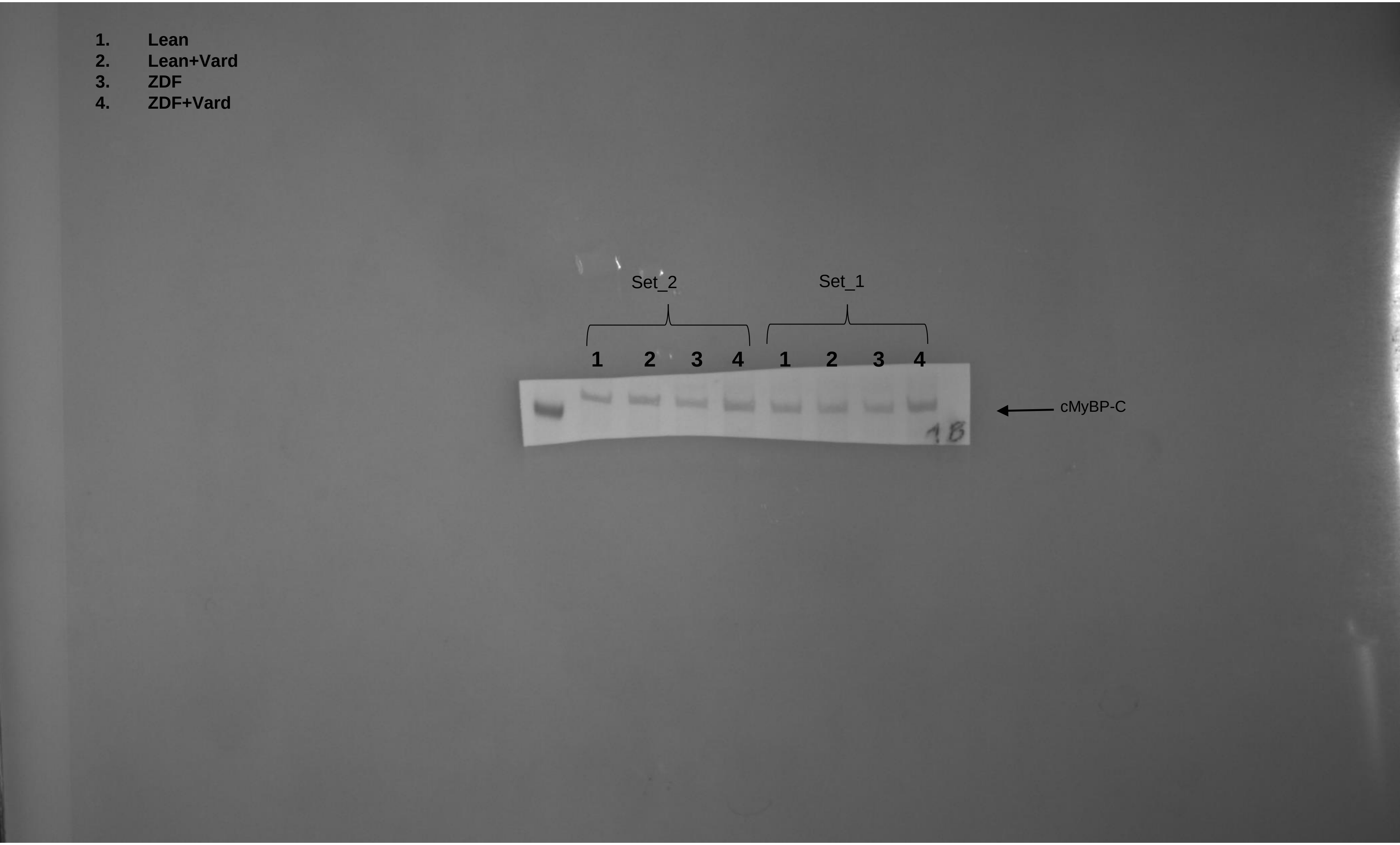

Left ventricle_Cardiac myosin binding protein C_original membranes_ Ser-282-P

1. Lean
2. Lean+Vard
3. ZDF
4. ZDF+Vard

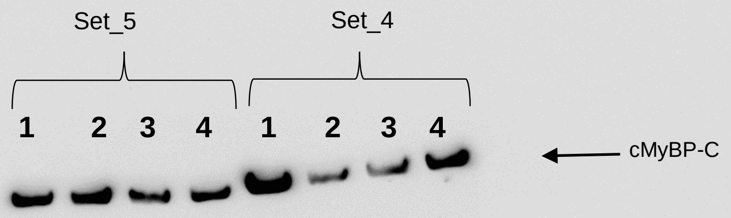

Left ventricle_Cardiac myosin binding protein C_original membranes_ Ser-282-P-Blot stain

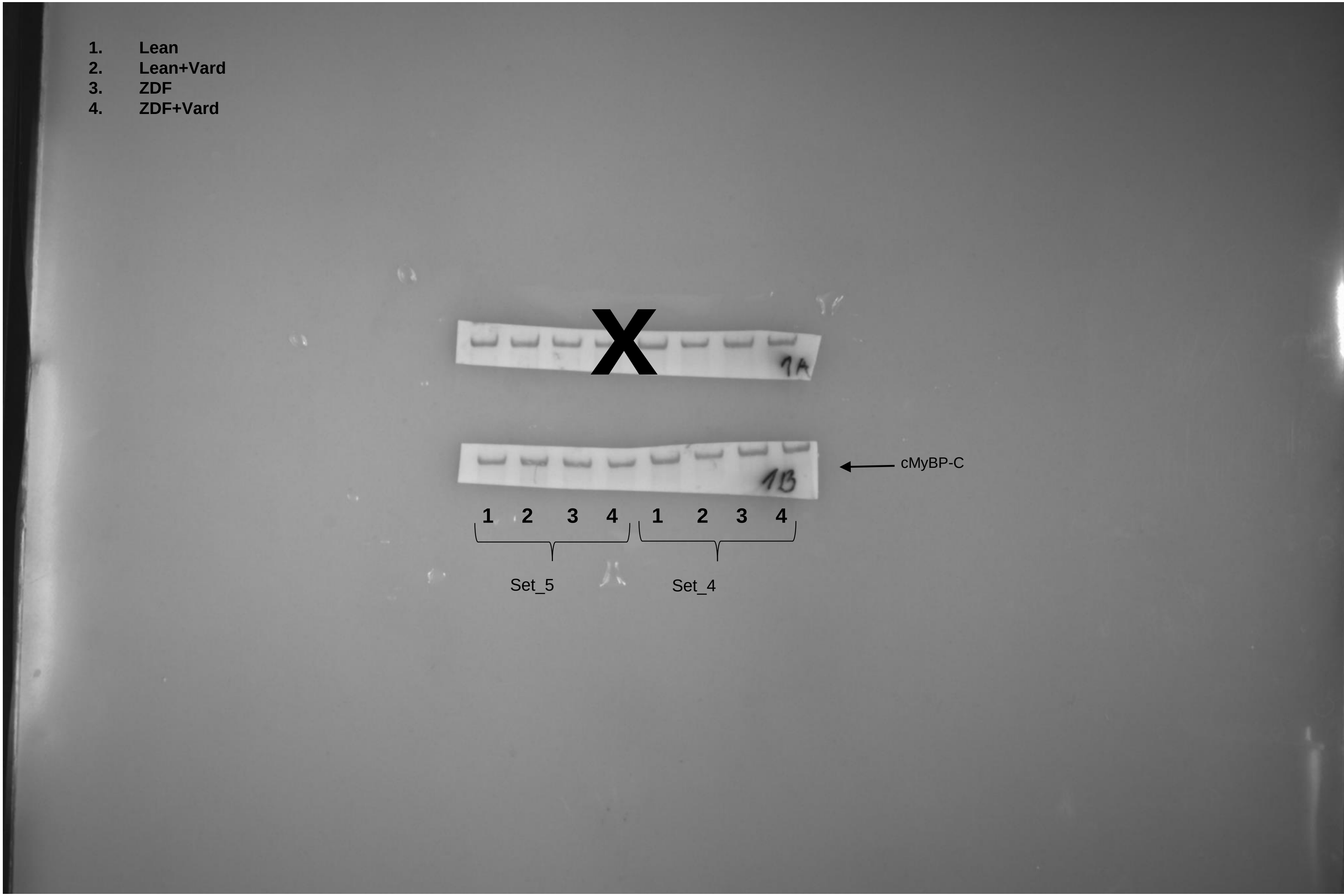

Left ventricle_Cardiac myosin binding protein C_original membranes_ Ser-282-P

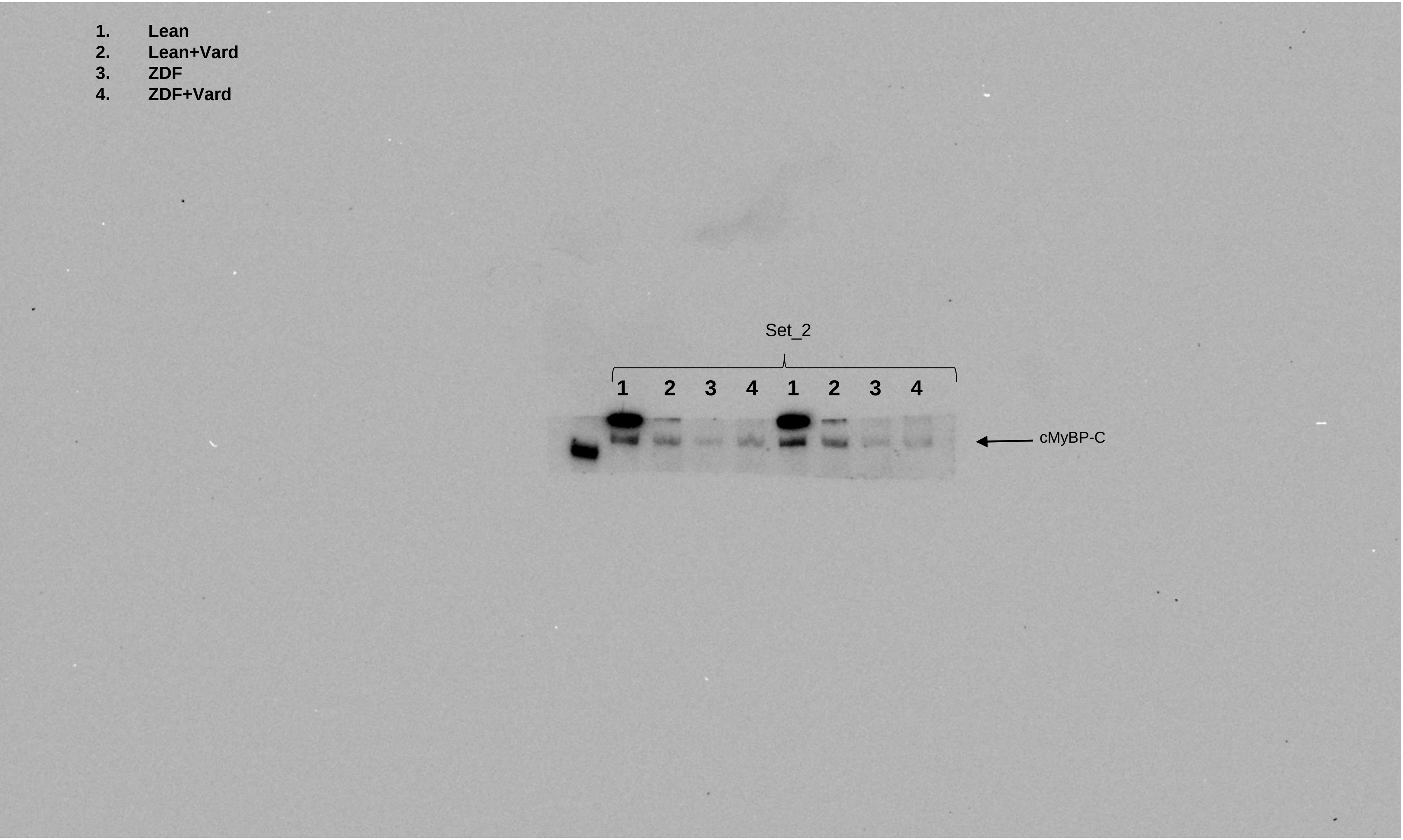

Left ventricle_Cardiac myosin binding protein C_original membranes_ Ser-282-P- Blot stain

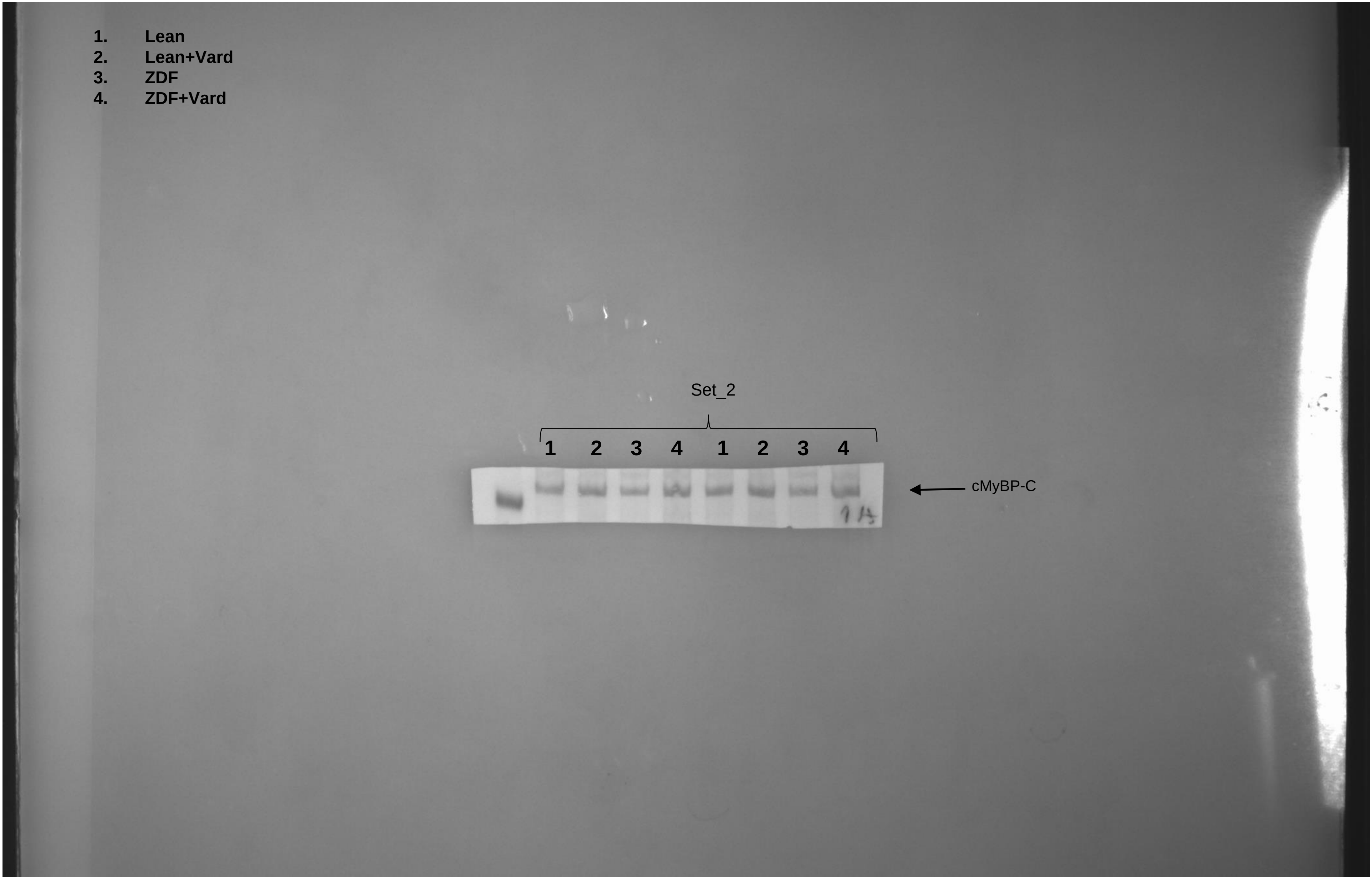

Left ventricle_Titin-P_original gel

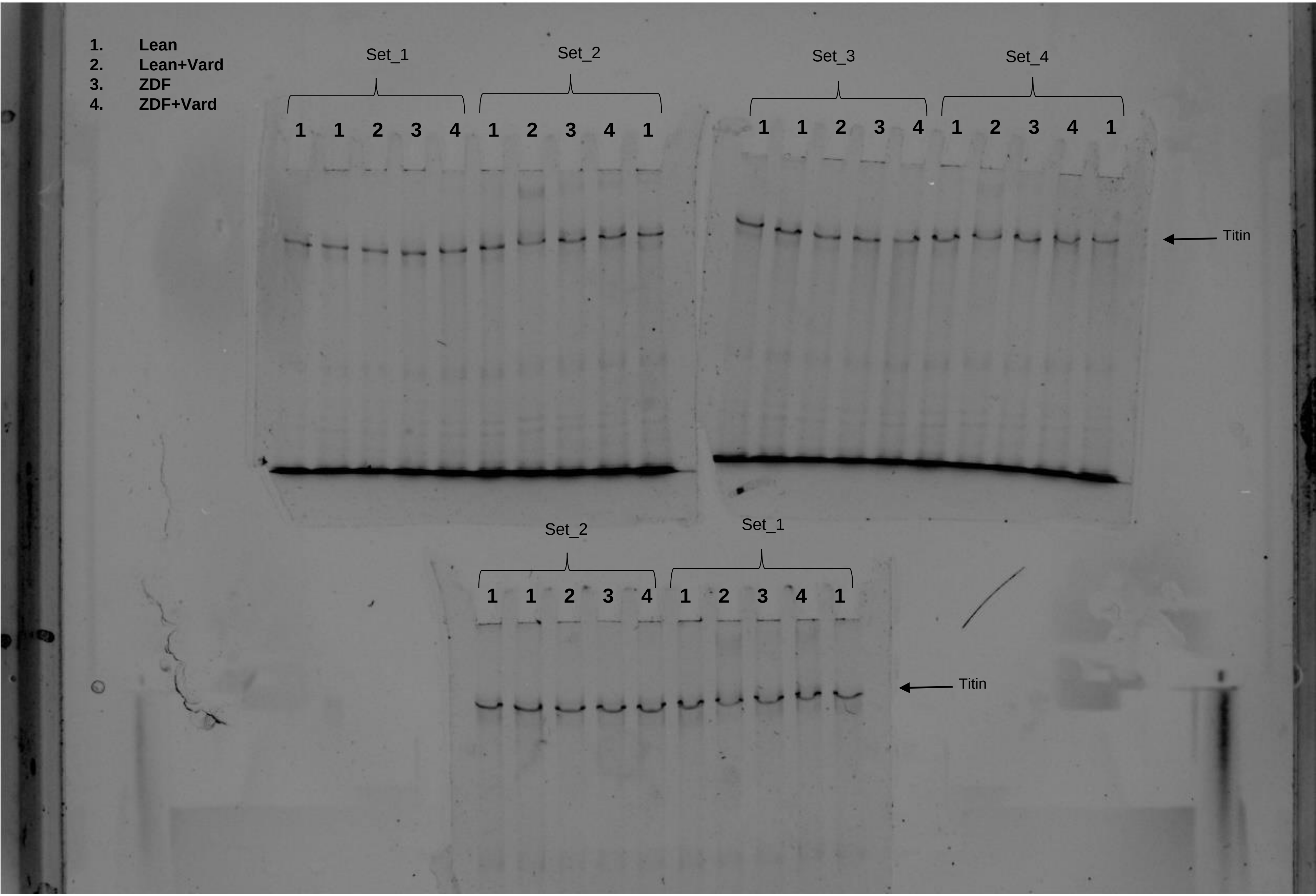

Left ventricle_Titin-total protein_original gel

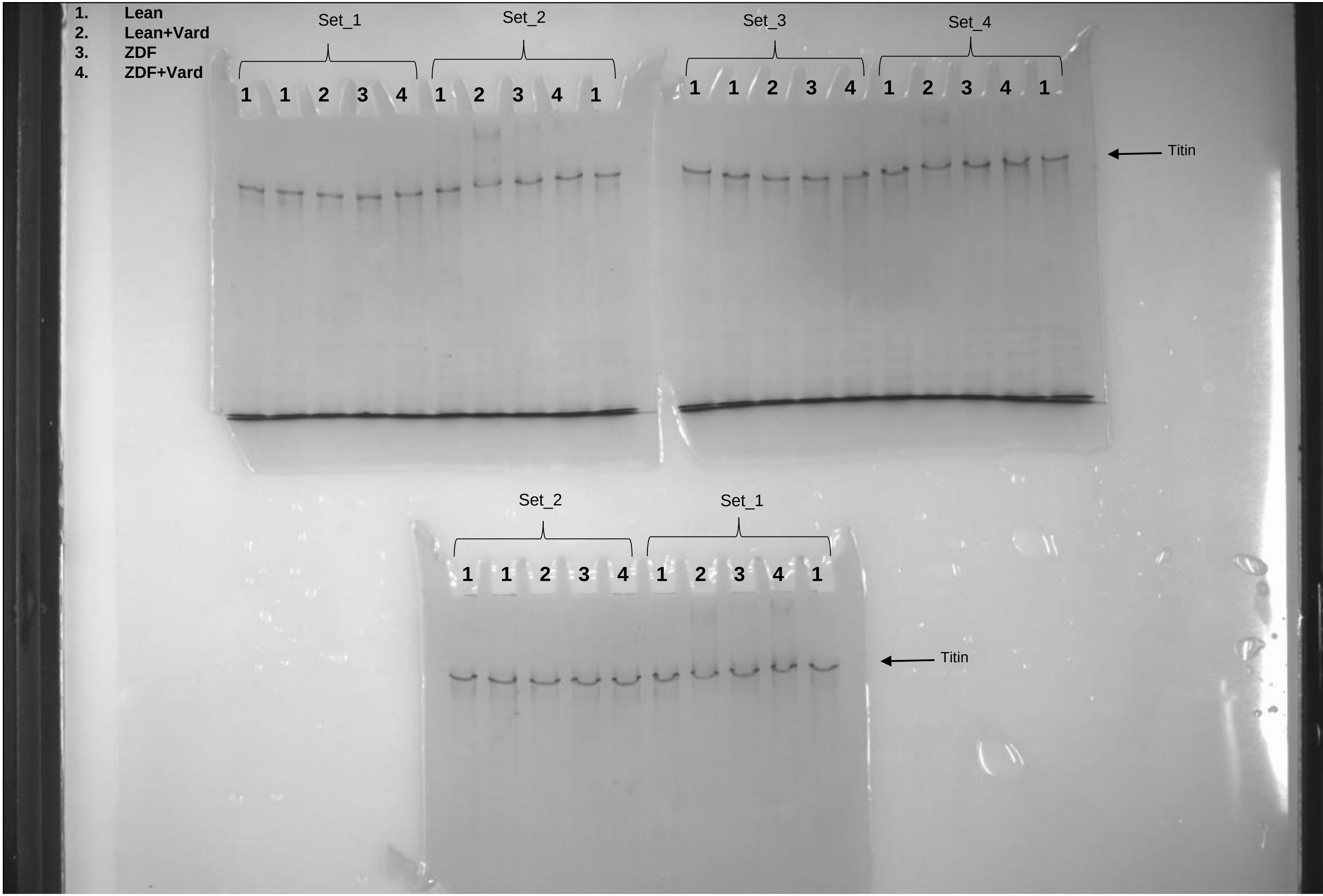

Left ventricle_Titin-P_original gel

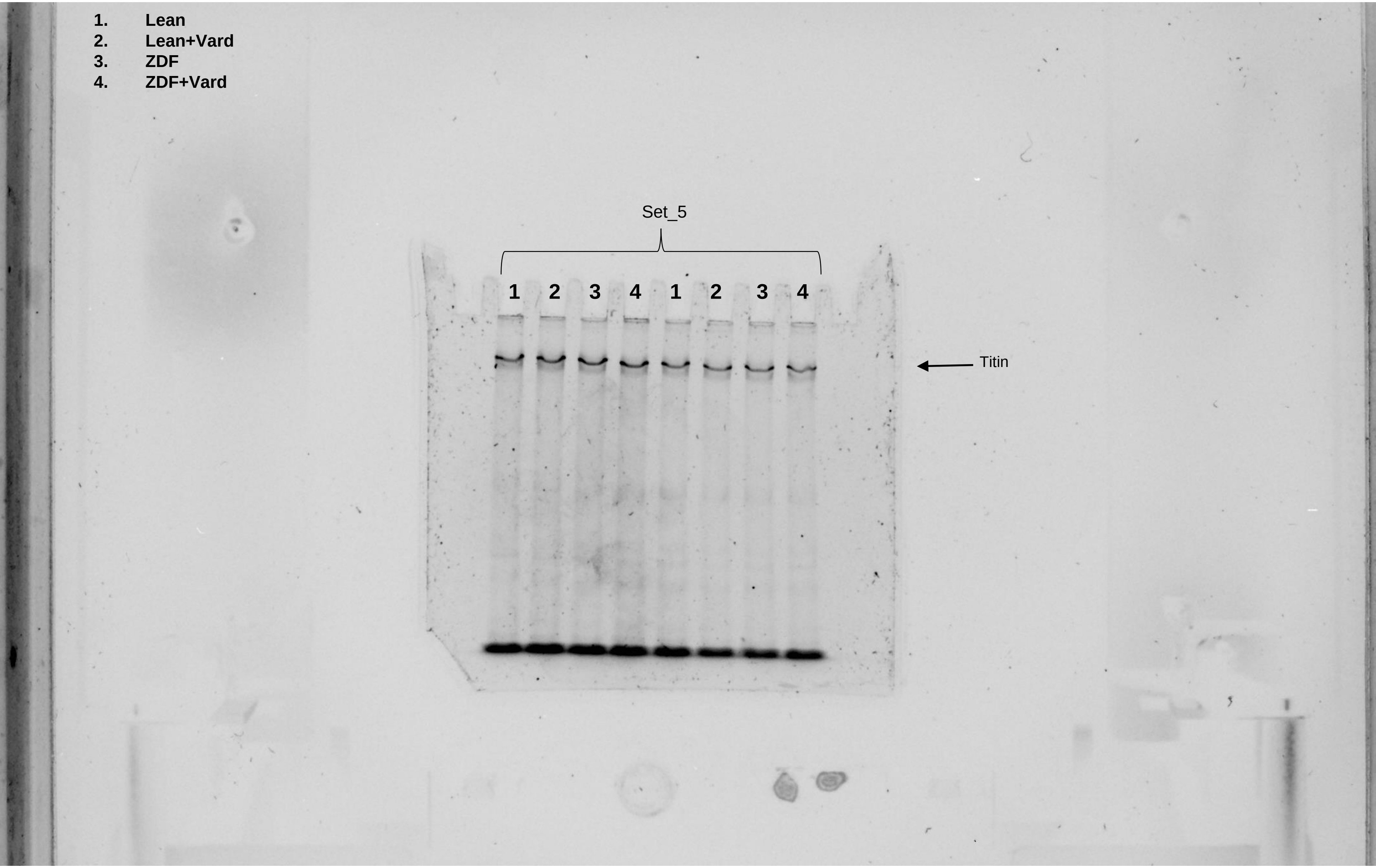

Left ventricle_Titin-total protein_original gel

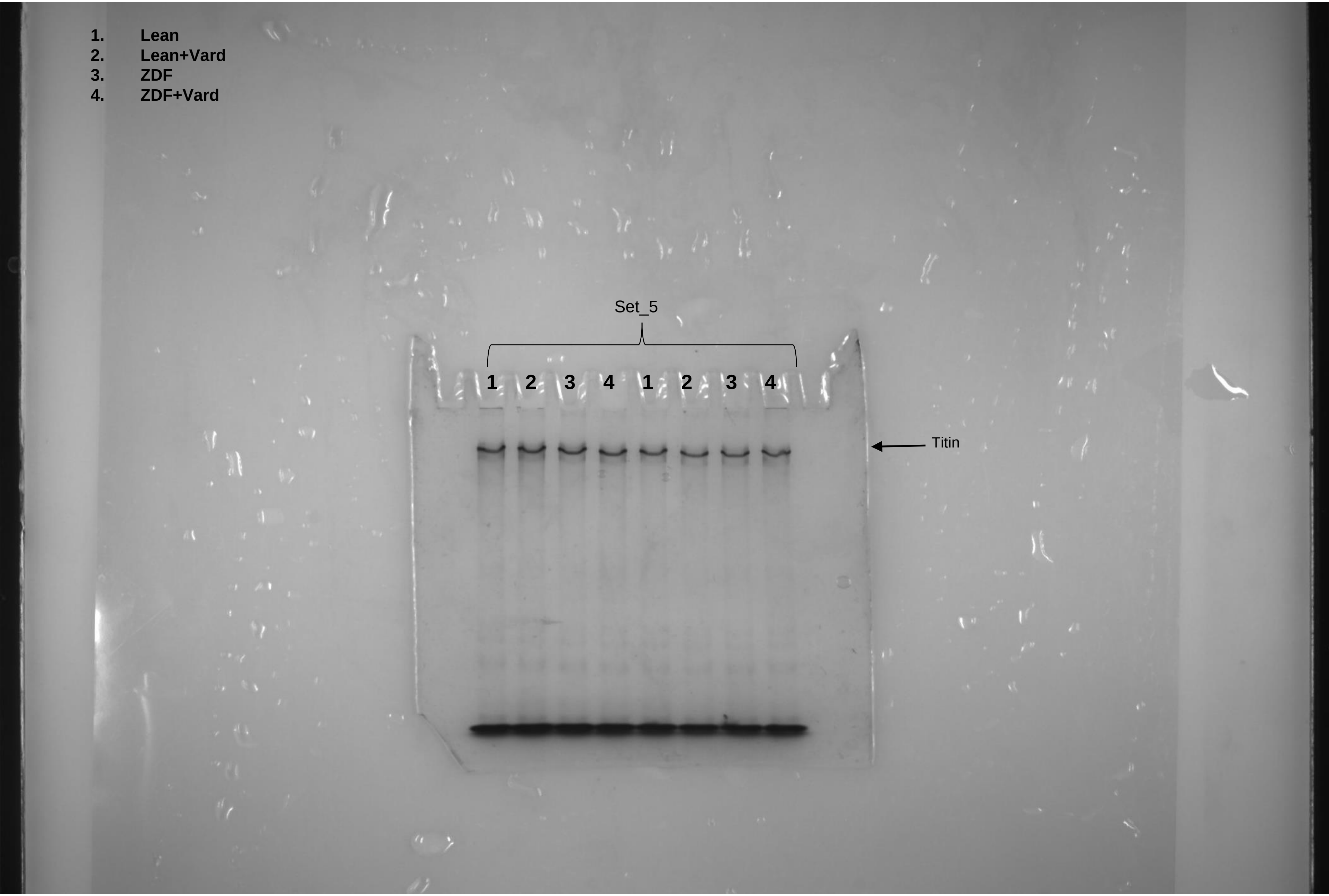

Left ventricle_Titin-P_original gel

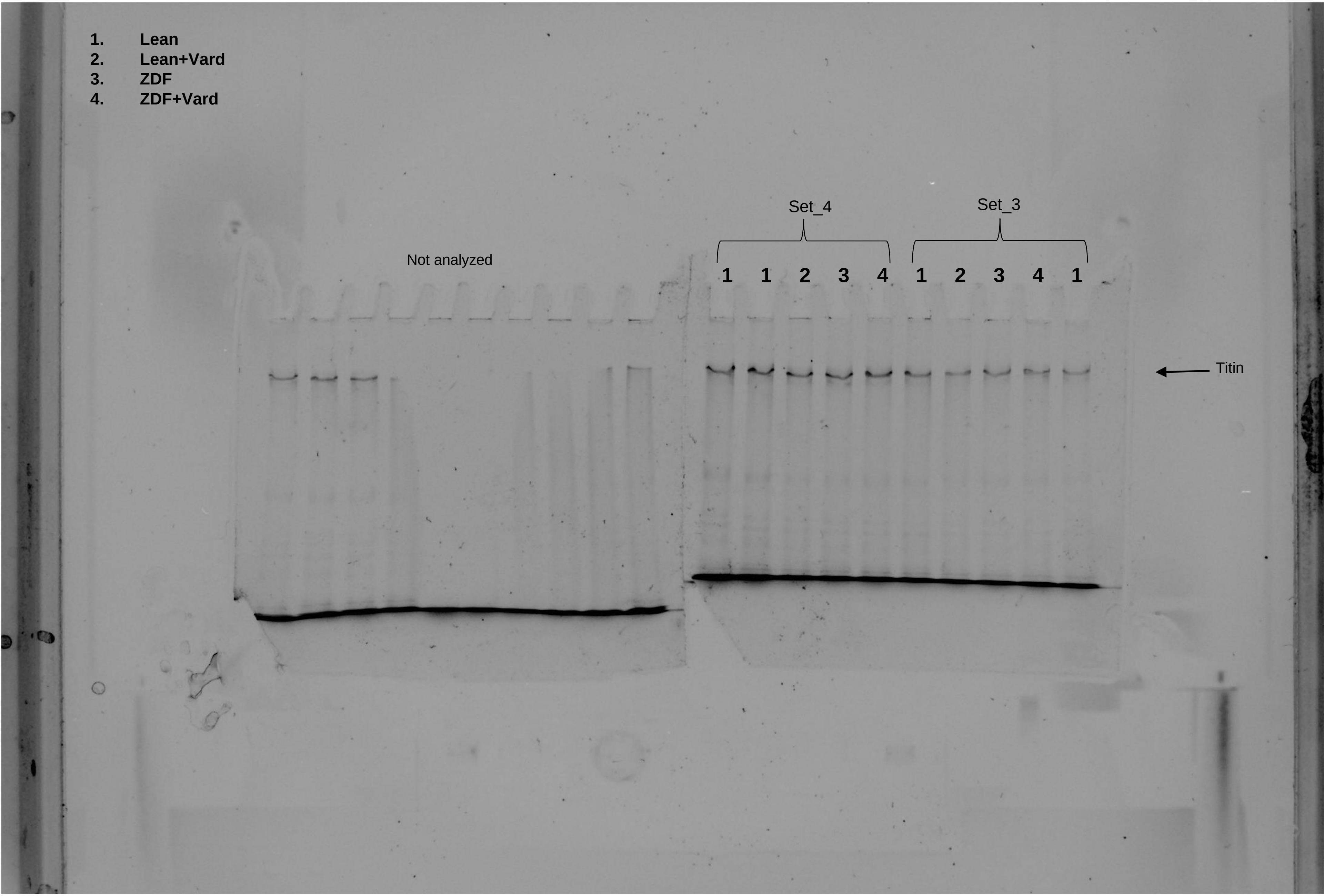

Left ventricle_Titin-total protein_original gel

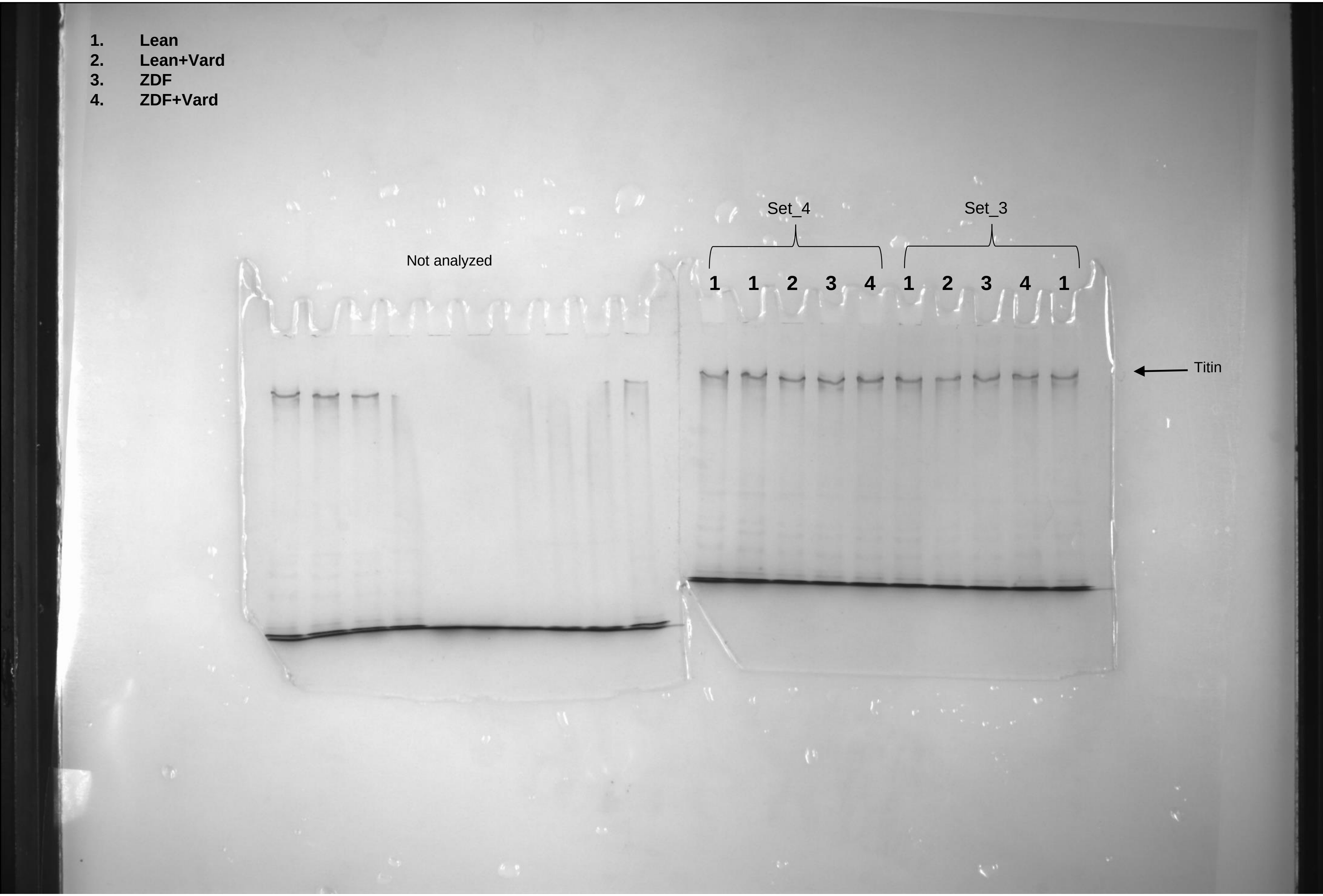

Right ventricle_Cardiac Troponin I_original membranes_ Ser-22/23-P

1. Lean
2. Lean+Vard
3. ZDF
4. ZDF+Vard

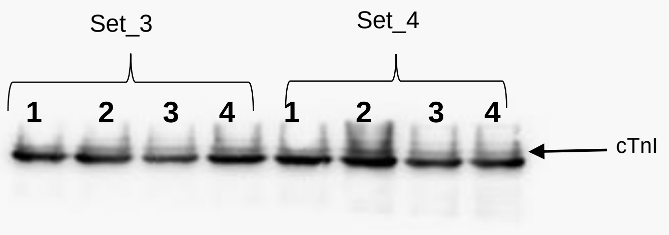

Right ventricle_Cardiac Troponin I_original membranes_ Ser-22/23-Blot stain

1. Lean
2. Lean+Vard
3. ZDF
4. ZDF+Vard

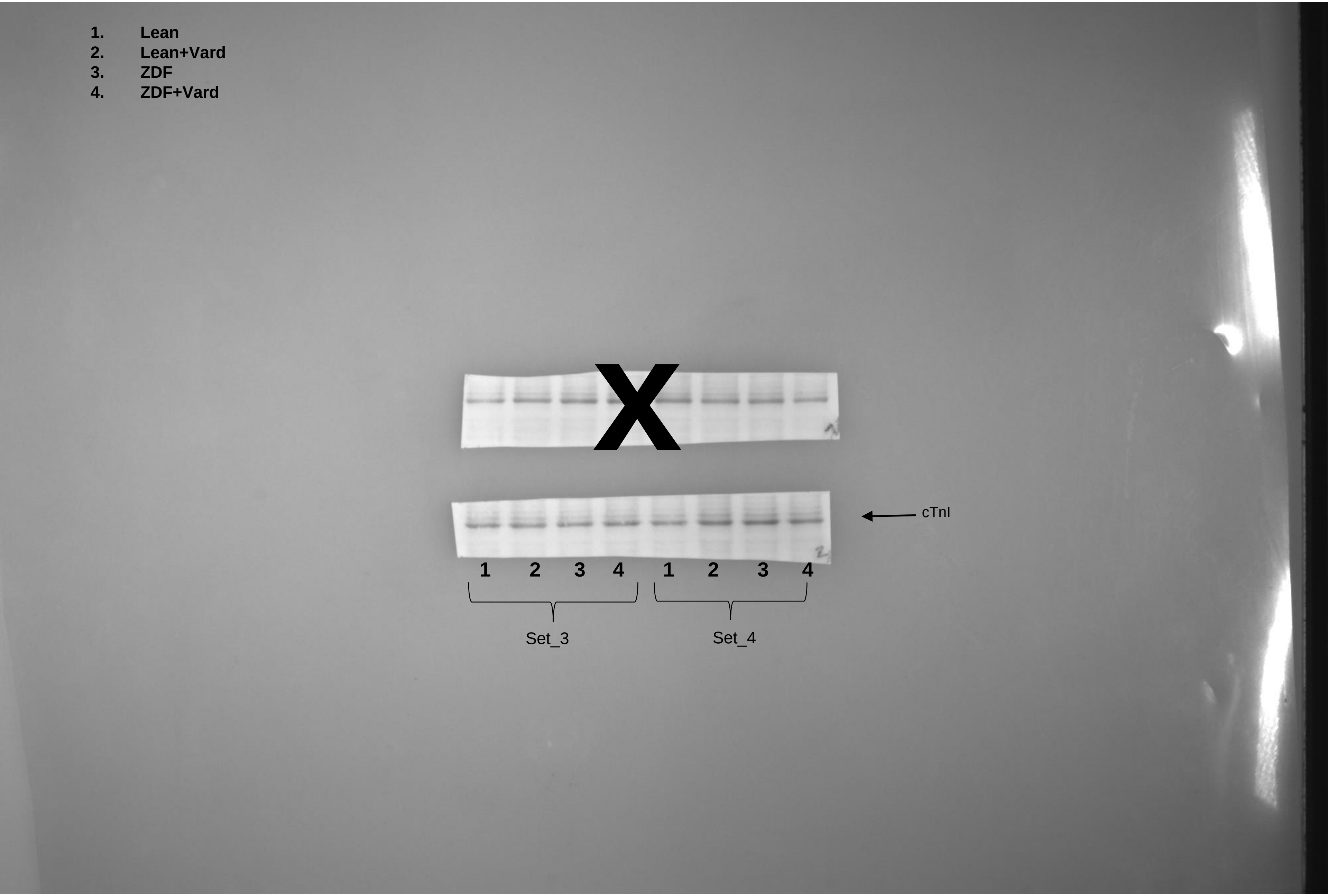

Right ventricle_Cardiac Troponin I_original membranes_ Ser-22/23

1. Lean
2. Lean+Vard
3. ZDF
4. ZDF+Vard

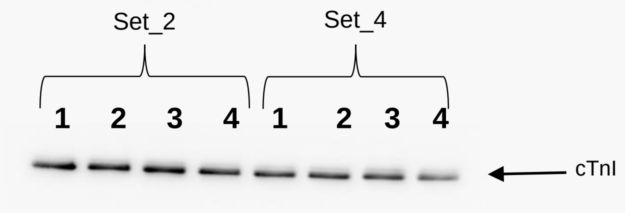

Right ventricle_Cardiac Troponin I_original membranes_ Ser-22/23-Blot stain

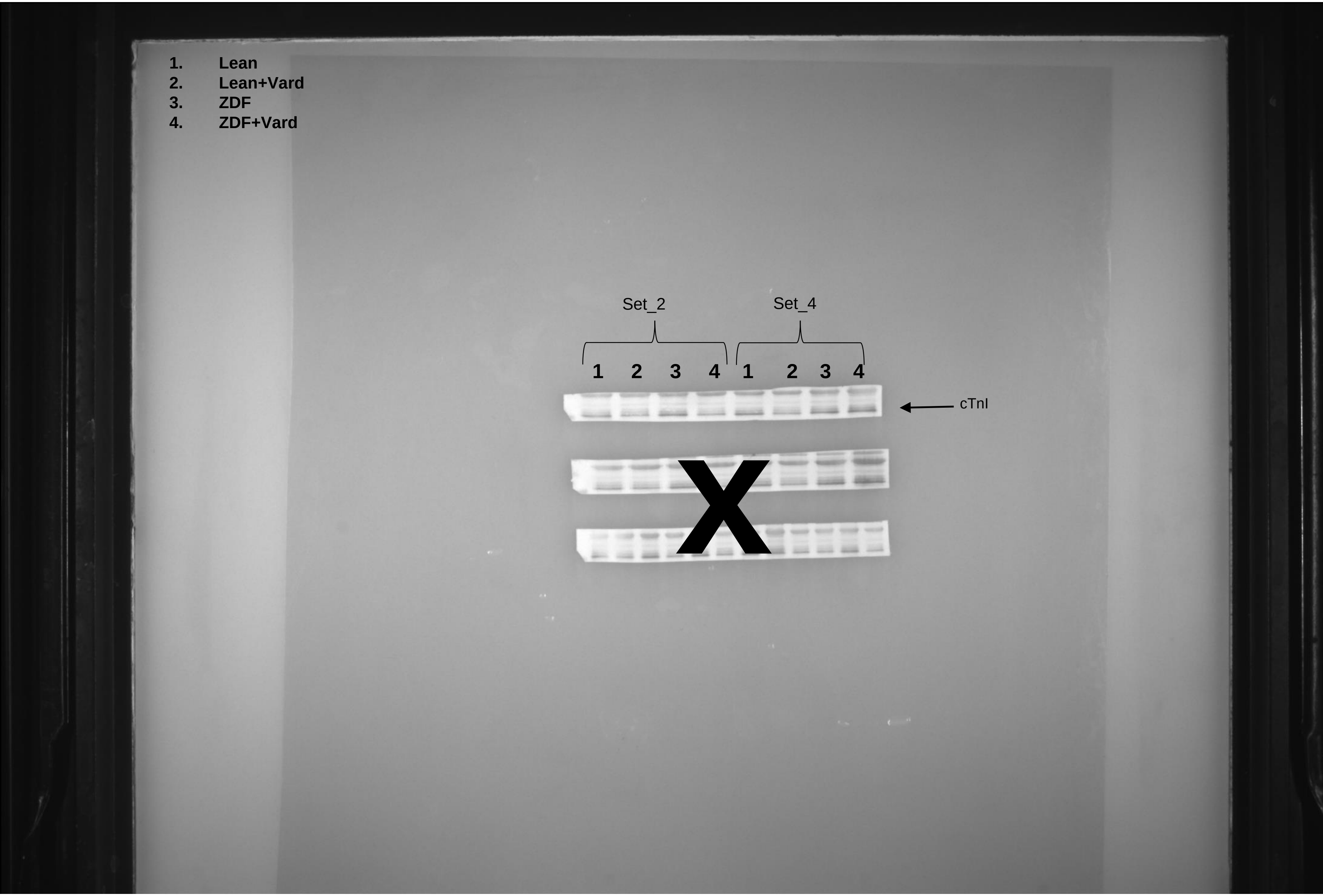

Right ventricle_Cardiac Troponin I_original membranes_ Ser-22/23-P

1. Lean
2. Lean+Vard
3. ZDF
4. ZDF+Vard

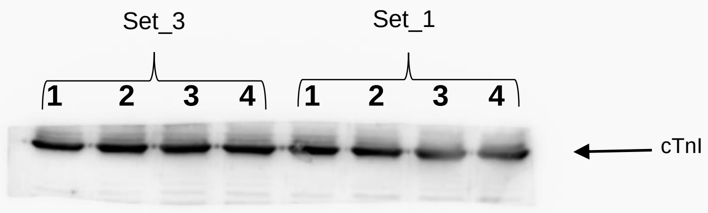

Right ventricle_Cardiac Troponin I_original membranes_ Ser-22/23-P-Blot stain

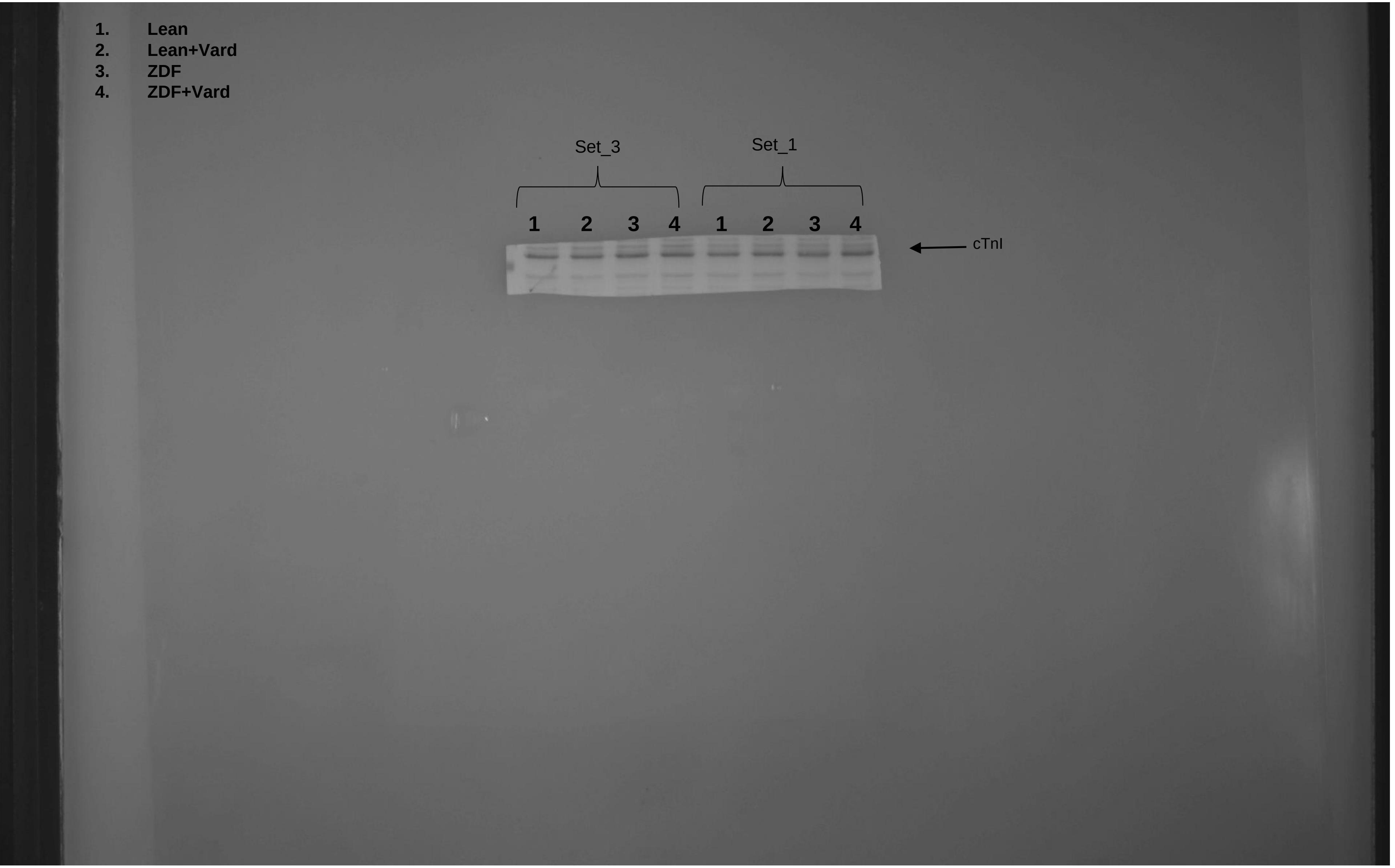

Right ventricle_Cardiac Troponin I_original membranes_ Ser-22/23-P

1. Lean
2. Lean+Vard
3. ZDF
4. ZDF+Vard

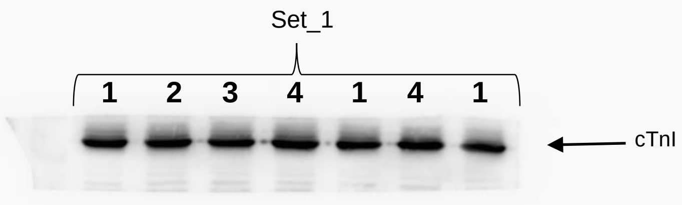

Right ventricle_Cardiac Troponin I_original membranes_ Ser-22/23-P-Blot stain

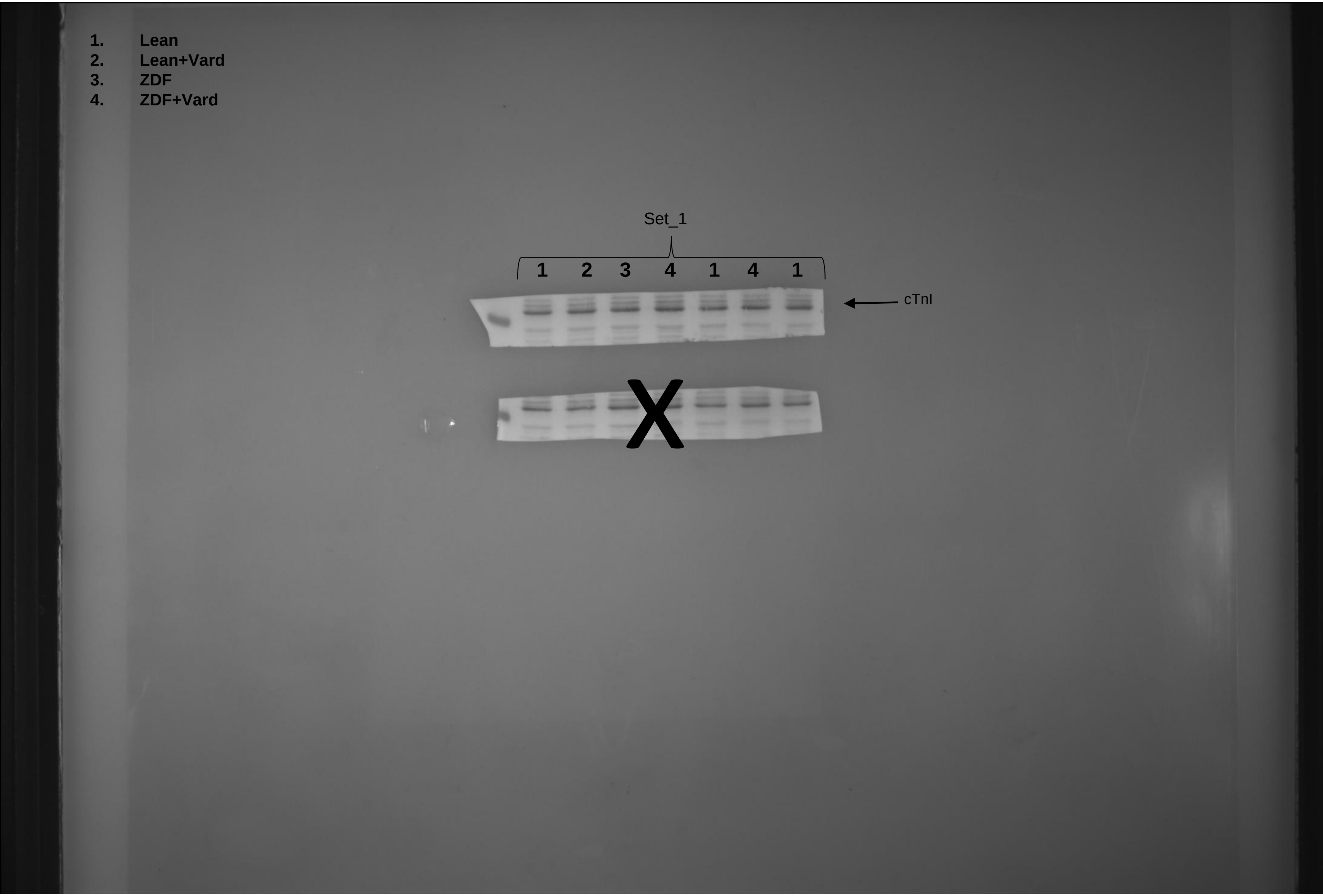

Right ventricle_Cardiac Troponin I_original membranes_ Ser-22/23-P

1. Lean
2. Lean+Vard
3. ZDF
4. ZDF+Vard

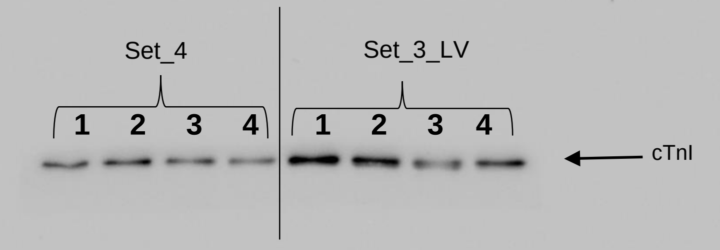

Right ventricle_Cardiac Troponin I_original membranes_ Ser-22/23-P-Blot stain

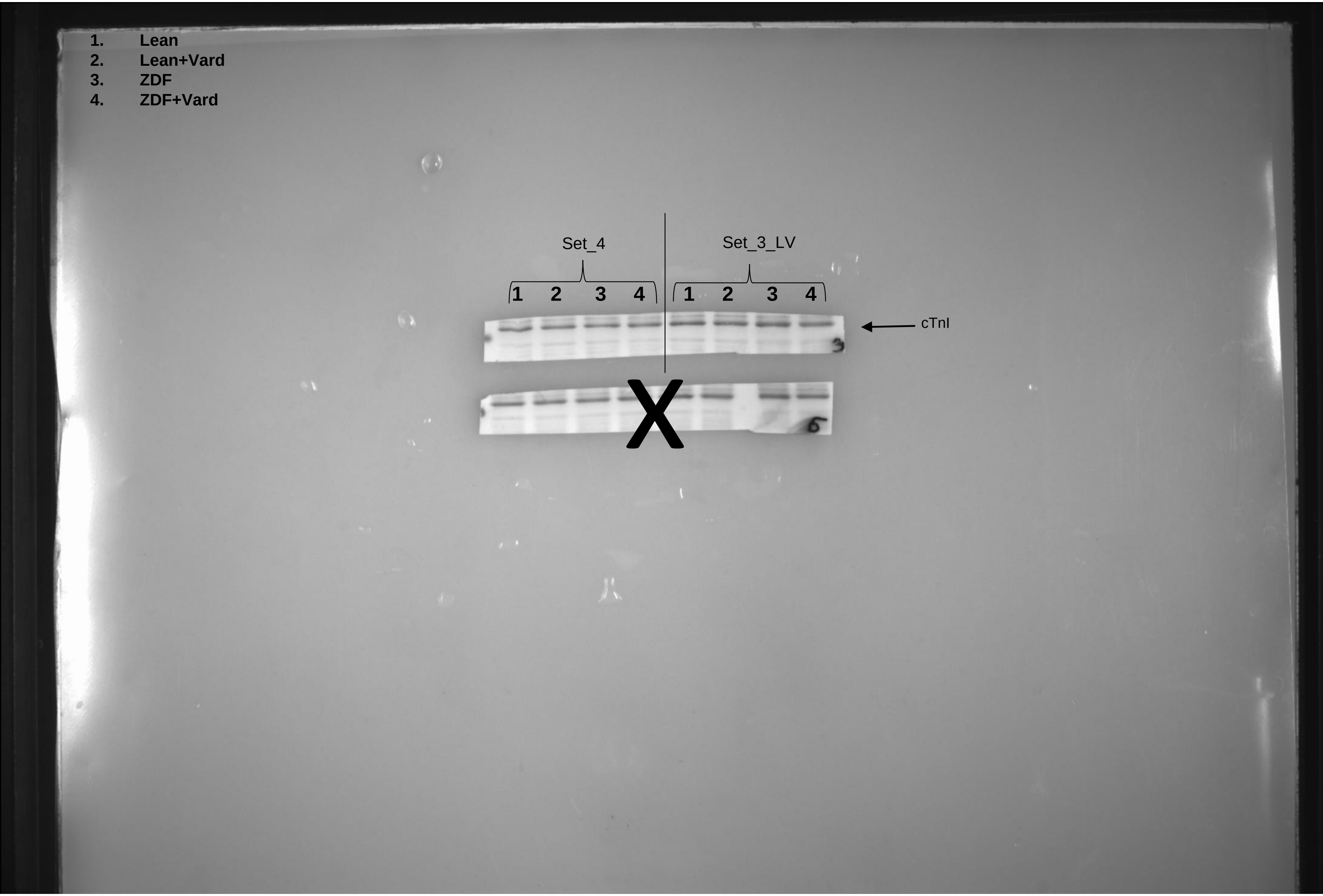

Right ventricle_Cardiac Troponin I_original membranes_ Ser-22/23-P-

1. Lean
2. Lean+Vard
3. ZDF
4. ZDF+Vard

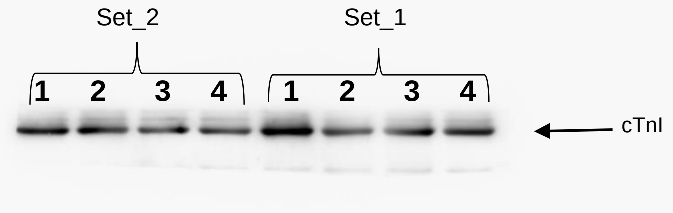

Right ventricle_Cardiac Troponin I_original membranes_ Ser-22/23-P-Blot stain

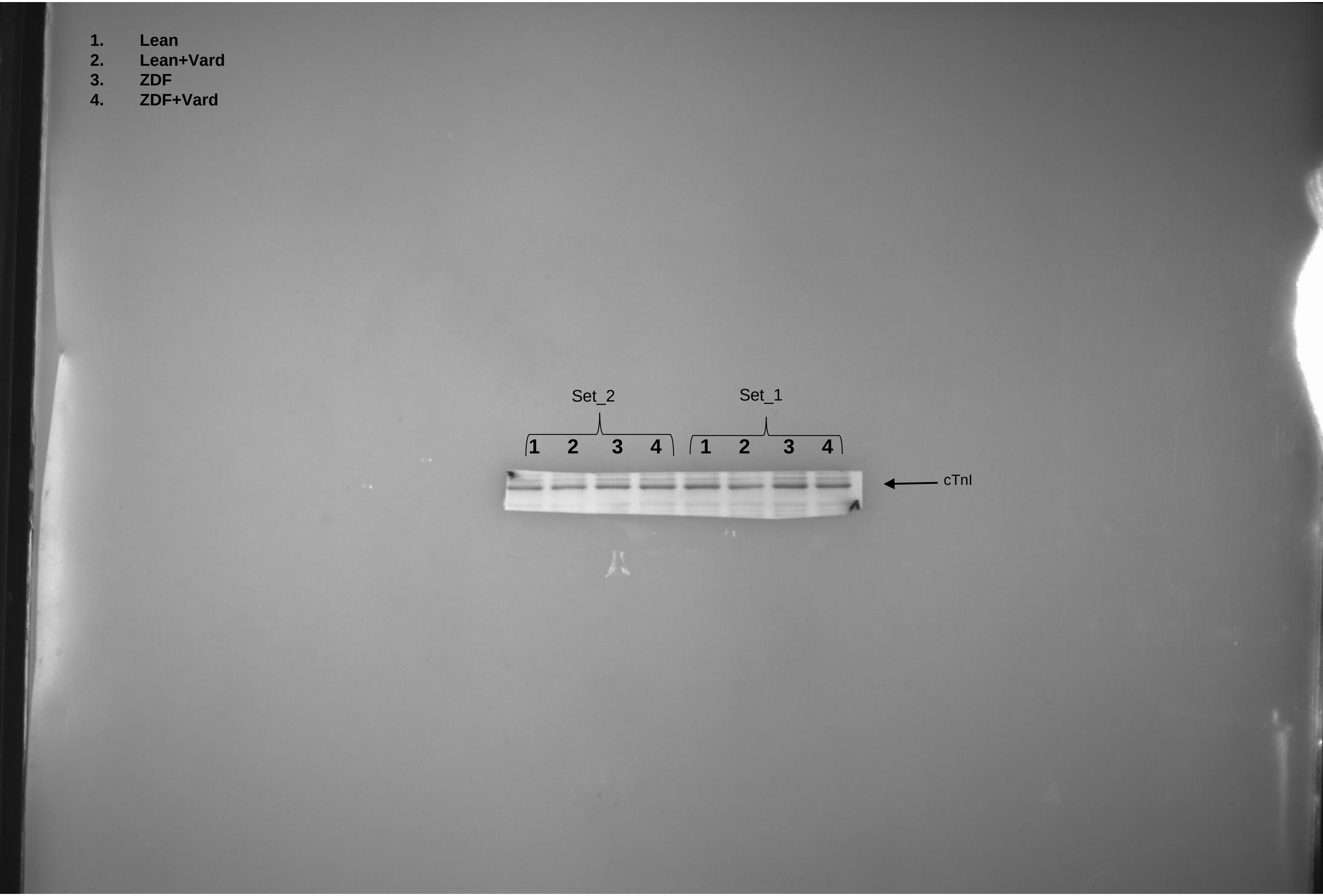

Right ventricle_Cardiac Troponin I_original membranes_ Ser-43-P

1. Lean
2. Lean+Vard
3. ZDF
4. ZDF+Vard

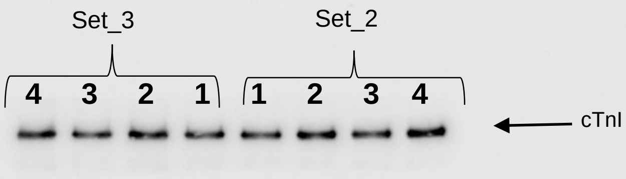

Right ventricle_Cardiac Troponin I_original membranes_ Ser-43-P-Blot stain

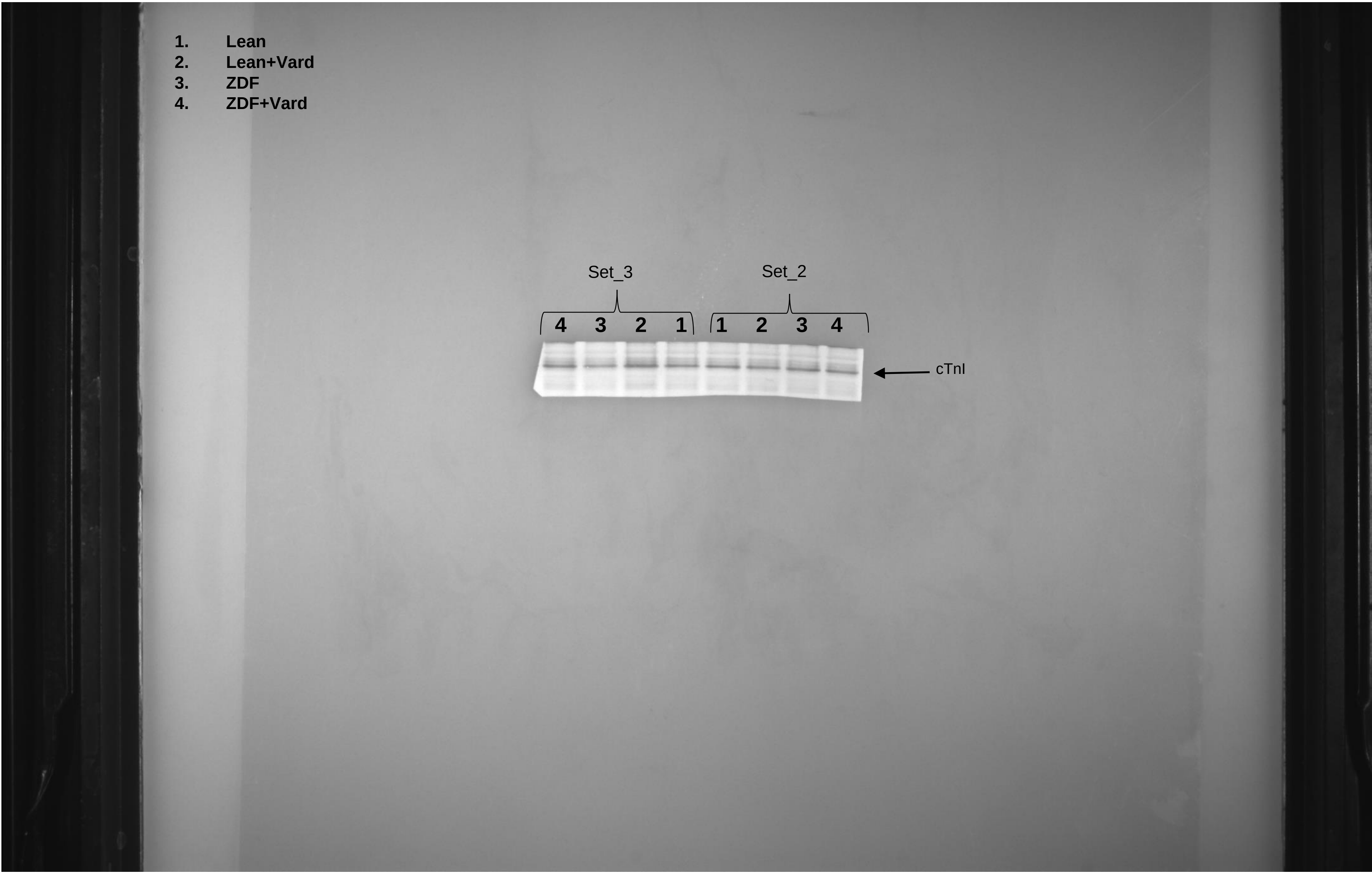

Right ventricle_Cardiac Troponin I_original membranes_ Ser-43-P

1. Lean
2. Lean+Vard
3. ZDF
4. ZDF+Vard

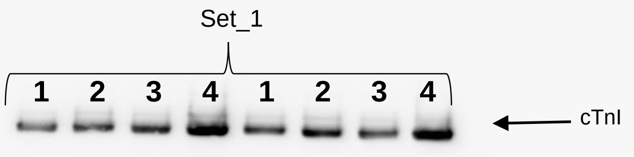

Right ventricle_Cardiac Troponin I_original membranes_ Ser-43-P-Blot stain

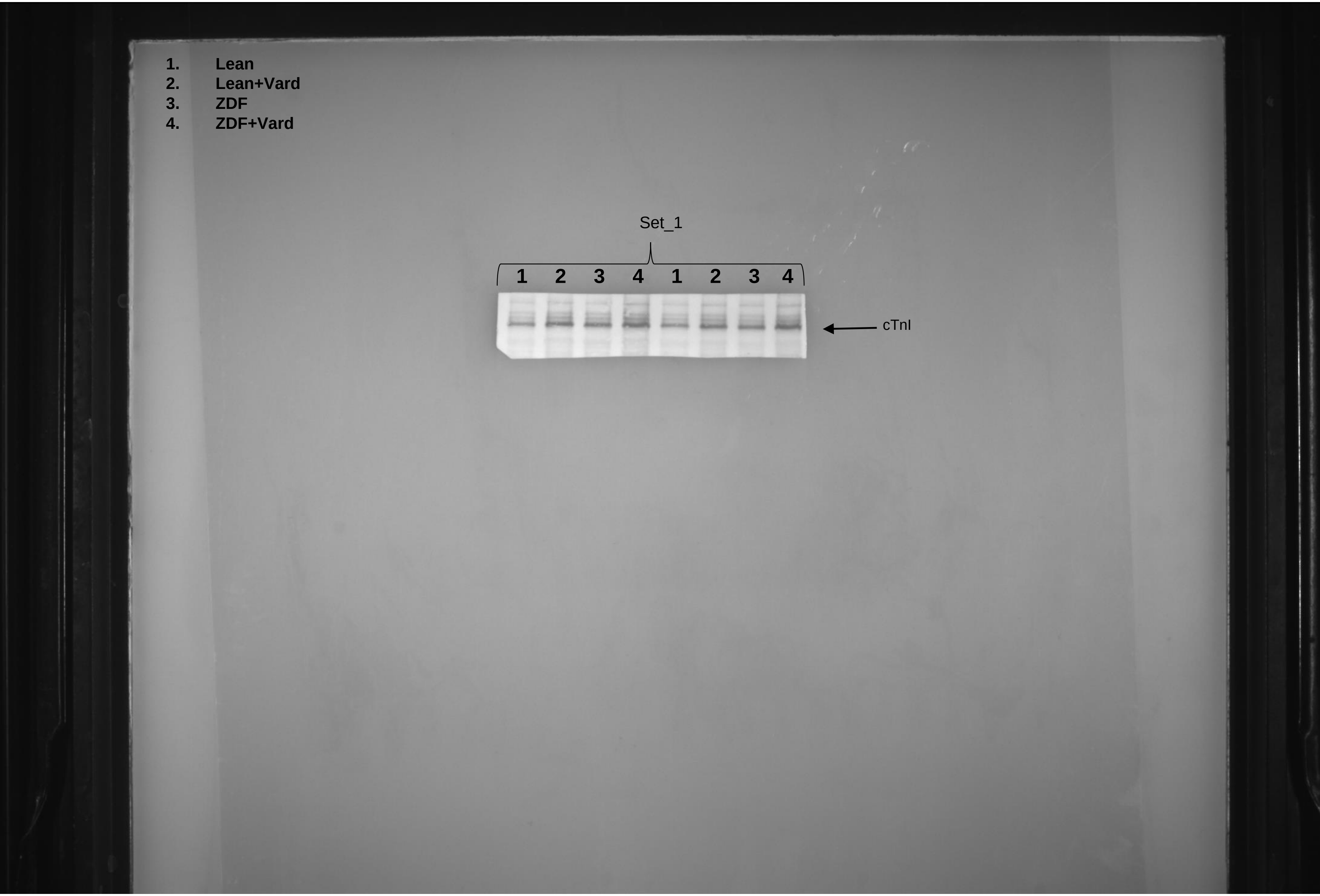

Right ventricle_Cardiac Troponin I_original membranes_ Ser-43-P

1. Lean
2. Lean+Vard
3. ZDF
4. ZDF+Vard

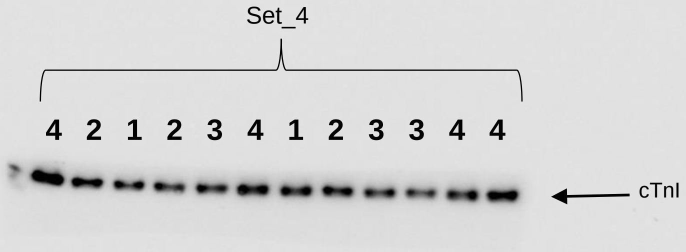

Right ventricle_Cardiac Troponin I_original membranes_ Ser-43-P-Blot stain

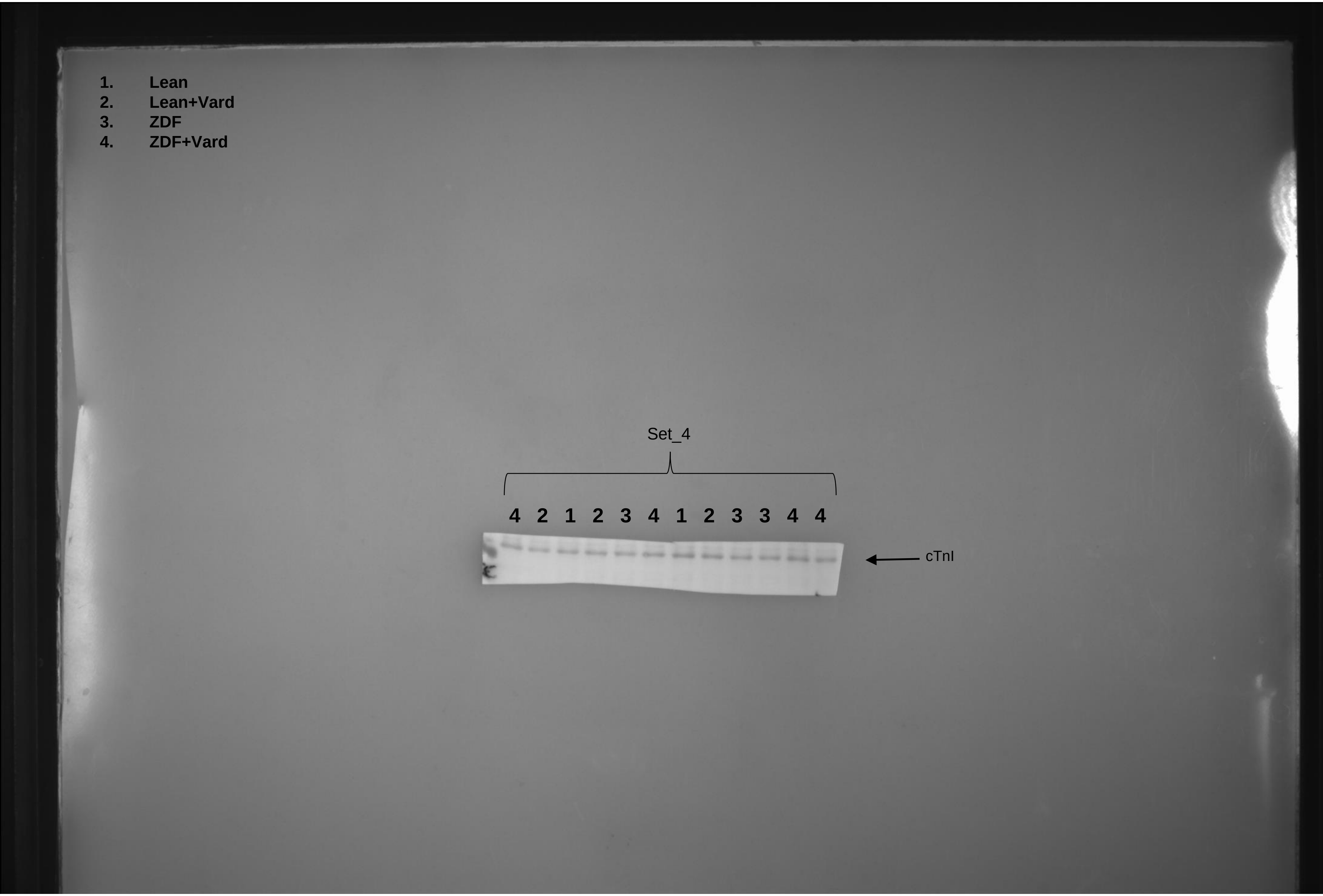

Right ventricle_Cardiac Troponin I_original membranes_ Ser-43-P

1. Lean
2. Lean+Vard
3. ZDF
4. ZDF+Vard

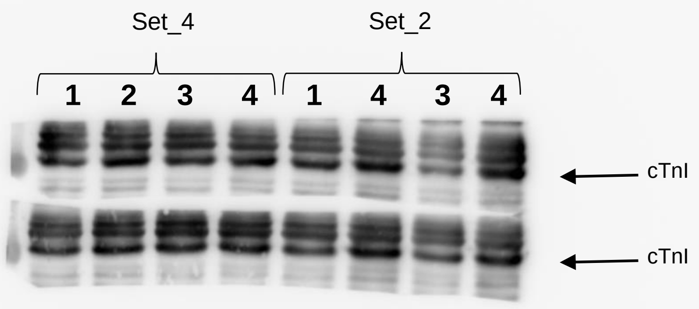

Right ventricle_Cardiac Troponin I_original membranes_ Ser-43-P-Blot stain

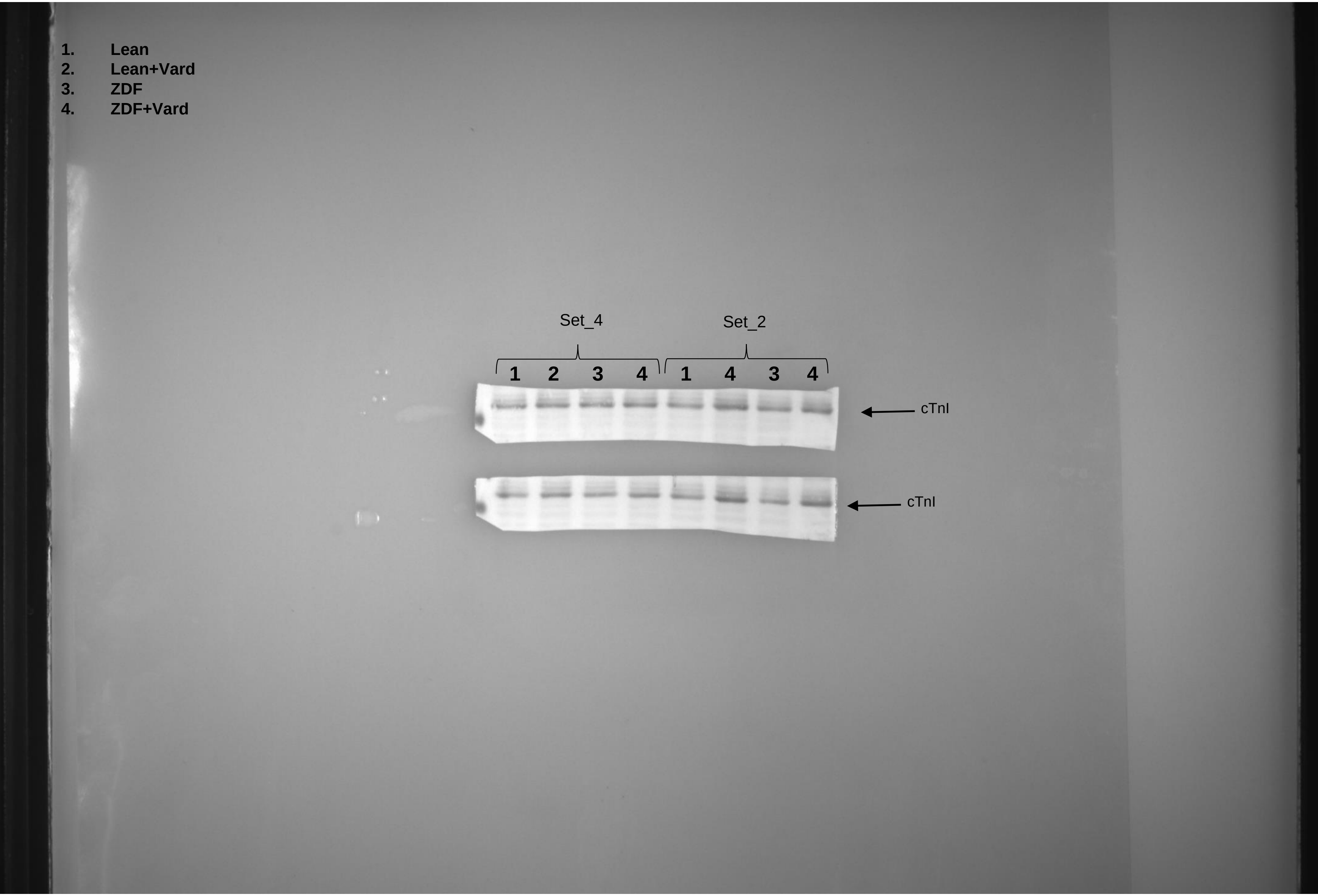

Right ventricle_Cardiac Troponin I_original membranes_ Ser-43-P

1. Lean
2. Lean+Vard
3. ZDF
4. ZDF+Vard

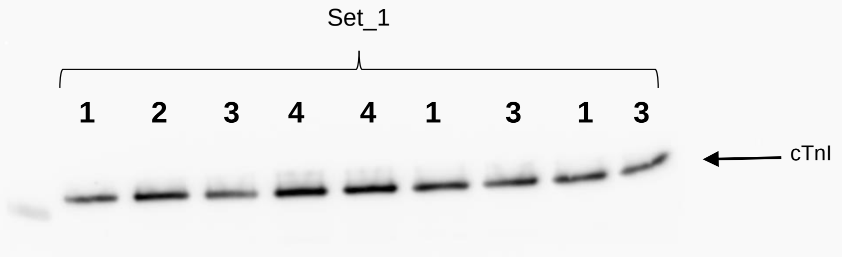

Right ventricle_Cardiac Troponin I_original membranes_ Ser-43-P-Blot stain

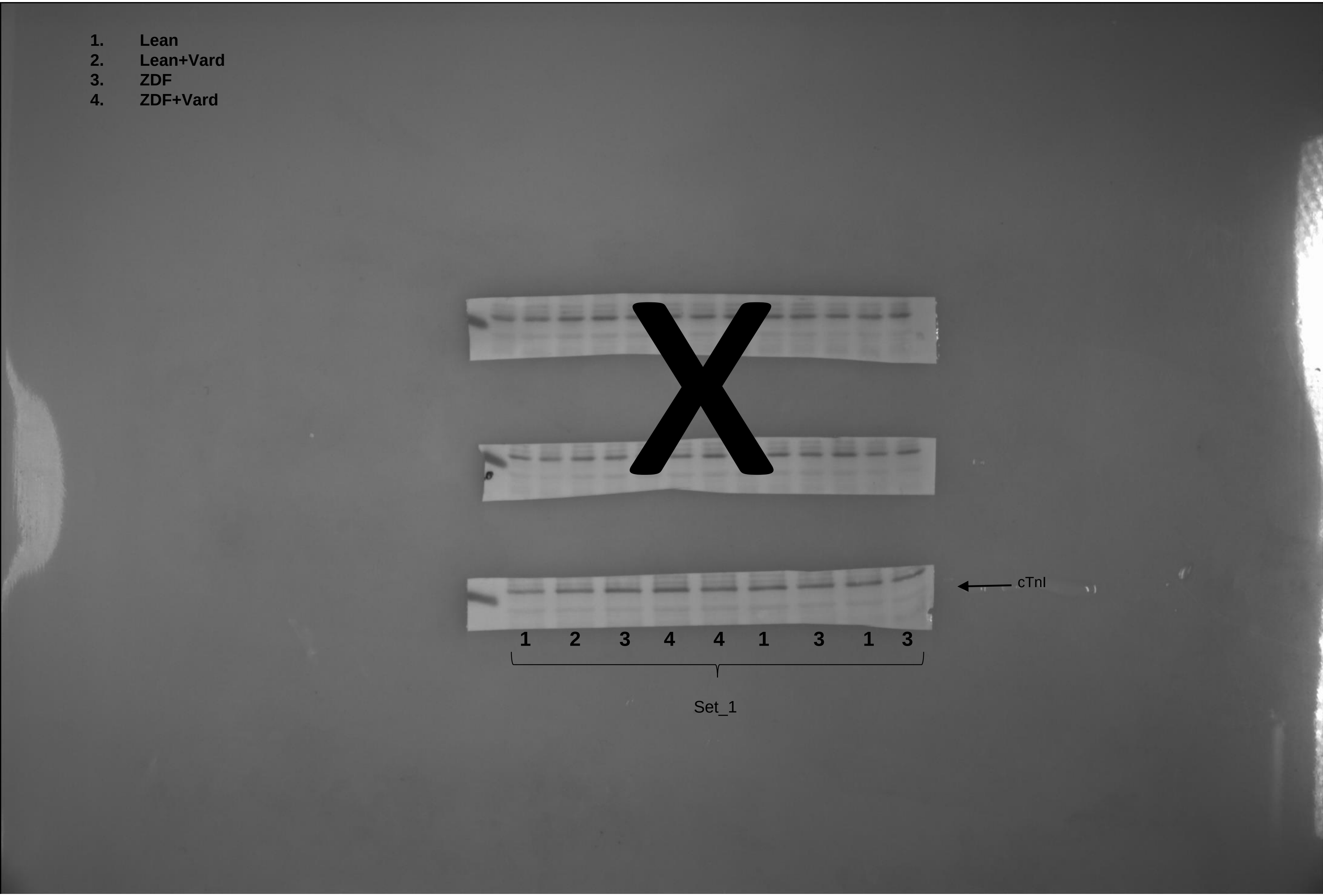

Right ventricle_Cardiac Troponin I_original membranes_ Thr-144-P

1. Lean
2. Lean+Vard
3. ZDF
4. ZDF+Vard

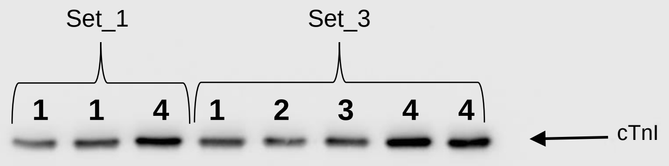

Right ventricle_Cardiac Troponin I_original membranes_ Thr-144-P-Blot stain

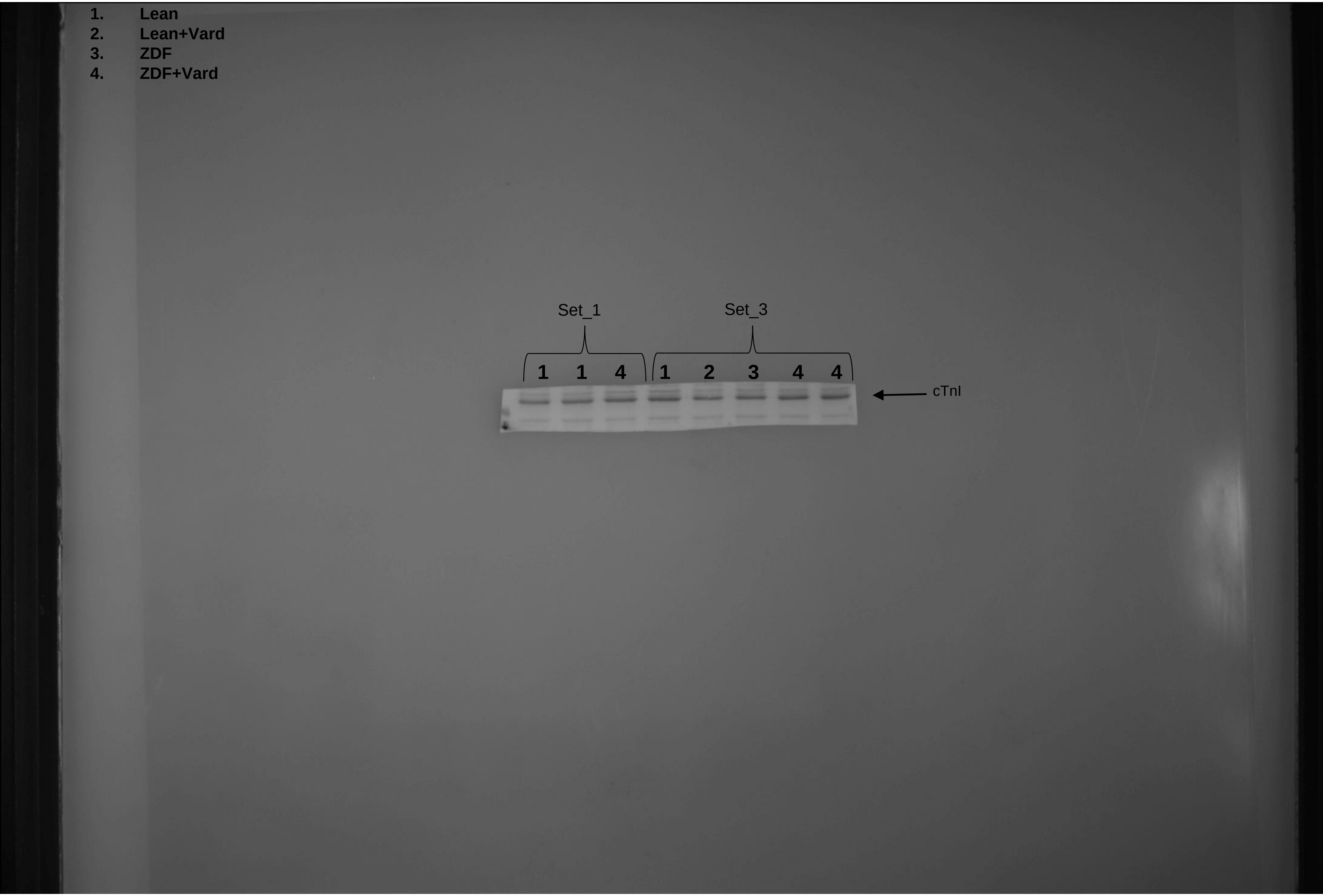

Right ventricle_Cardiac Troponin I_original membranes_ Thr-144-P

1. Lean
2. Lean+Vard
3. ZDF
4. ZDF+Vard

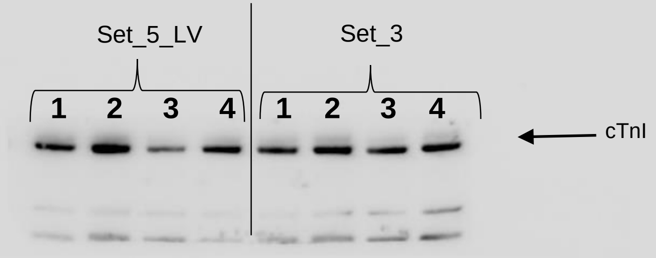

Right ventricle_Cardiac Troponin I_original membranes_ Thr-144-P-Blot stain

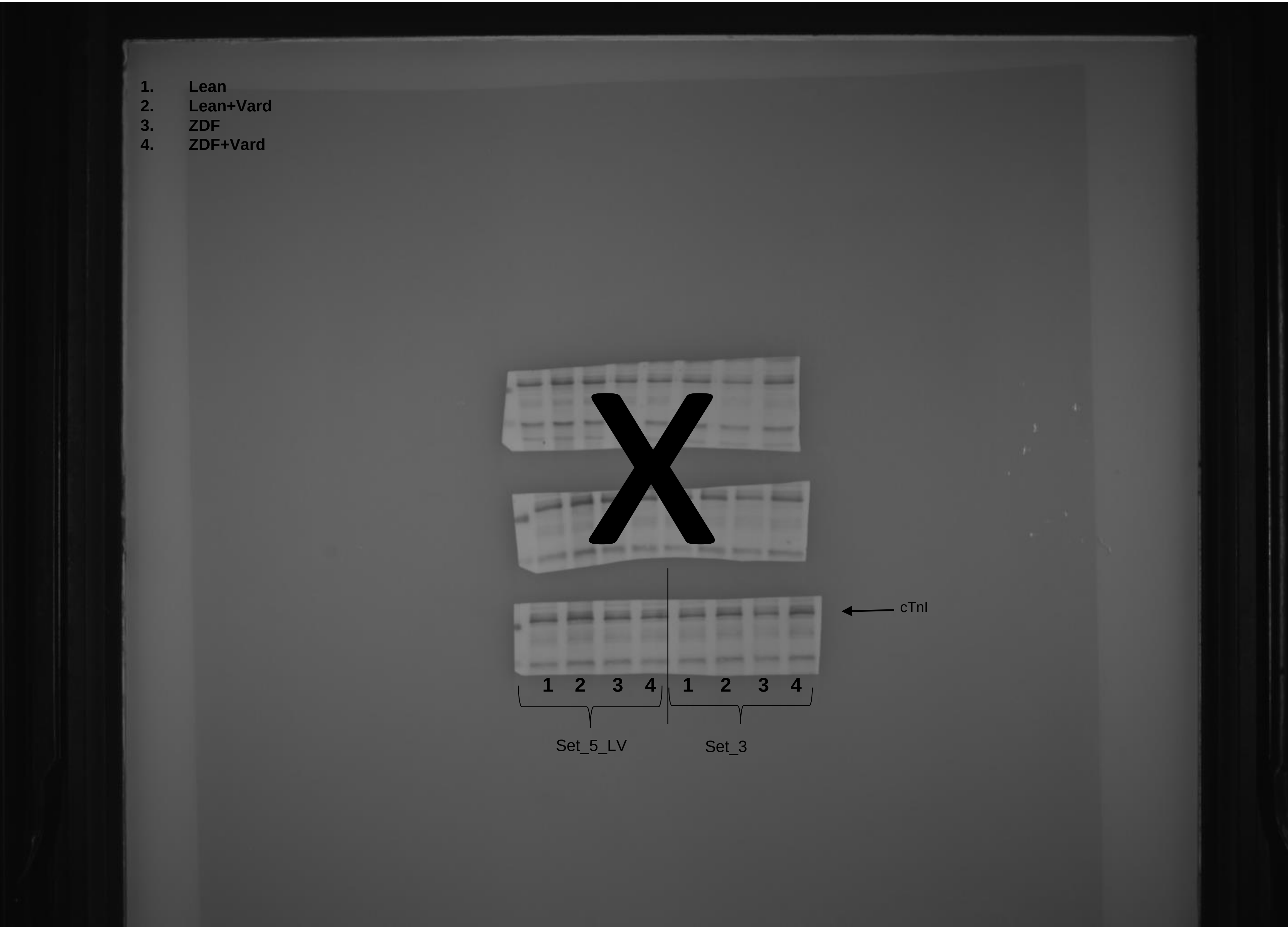

Right ventricle_Cardiac Troponin I_original membranes_ Thr-144-P

1. Lean
2. Lean+Vard
3. ZDF
4. ZDF+Vard

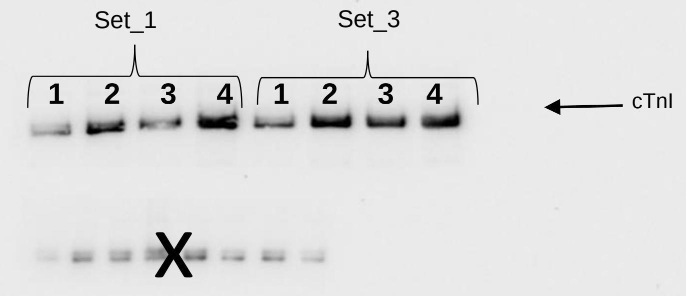

Right ventricle_Cardiac Troponin I_original membranes_ Thr-144-P-Blot stain

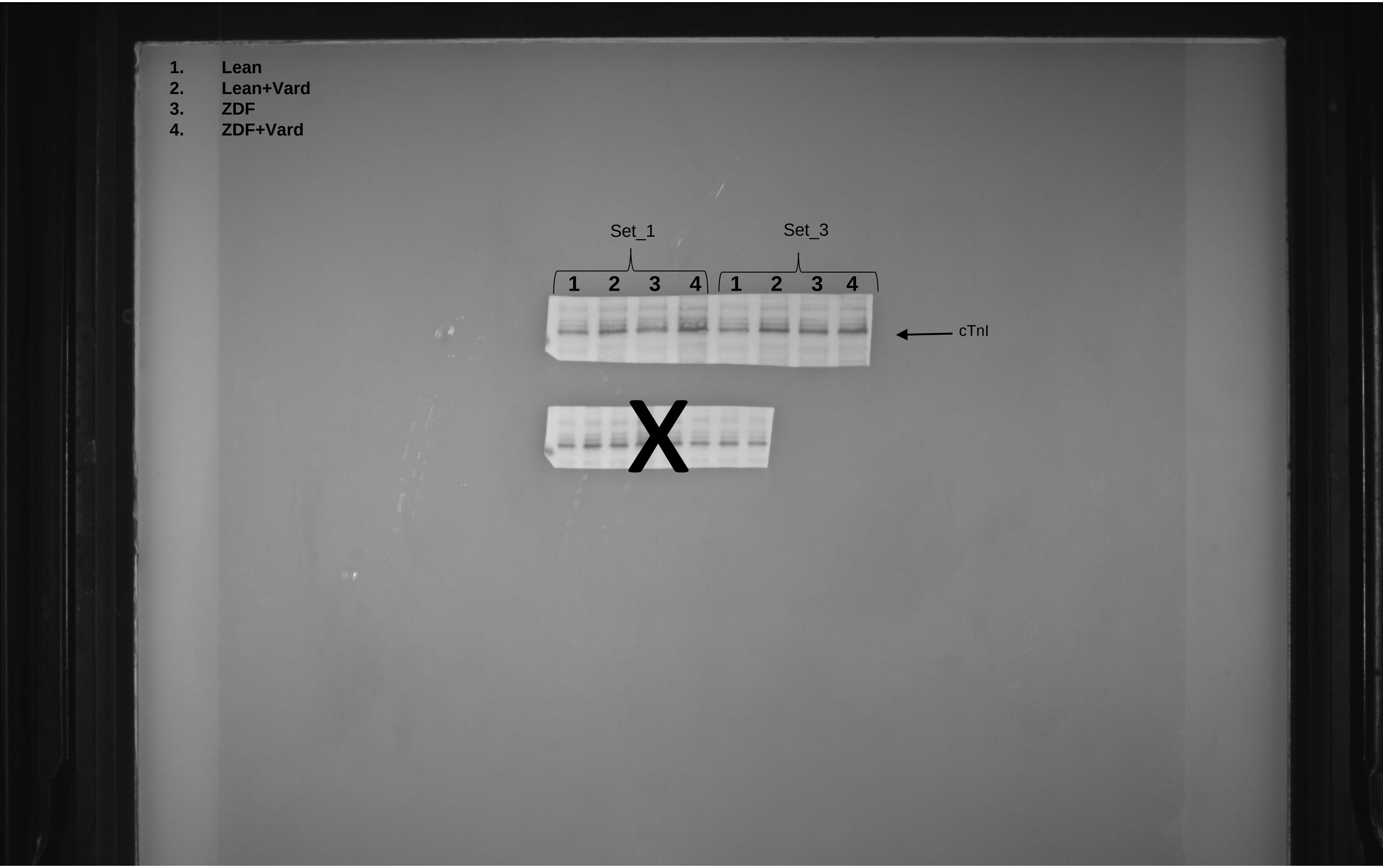

Right ventricle_Cardiac Troponin I_original membranes_ Thr-144-P

1. Lean
2. Lean+Vard
3. ZDF
4. ZDF+Vard

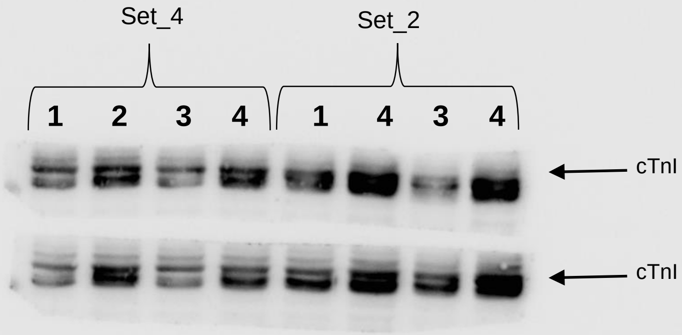

Right ventricle_Cardiac Troponin I_original membranes_ Thr-144-P-Blot stain

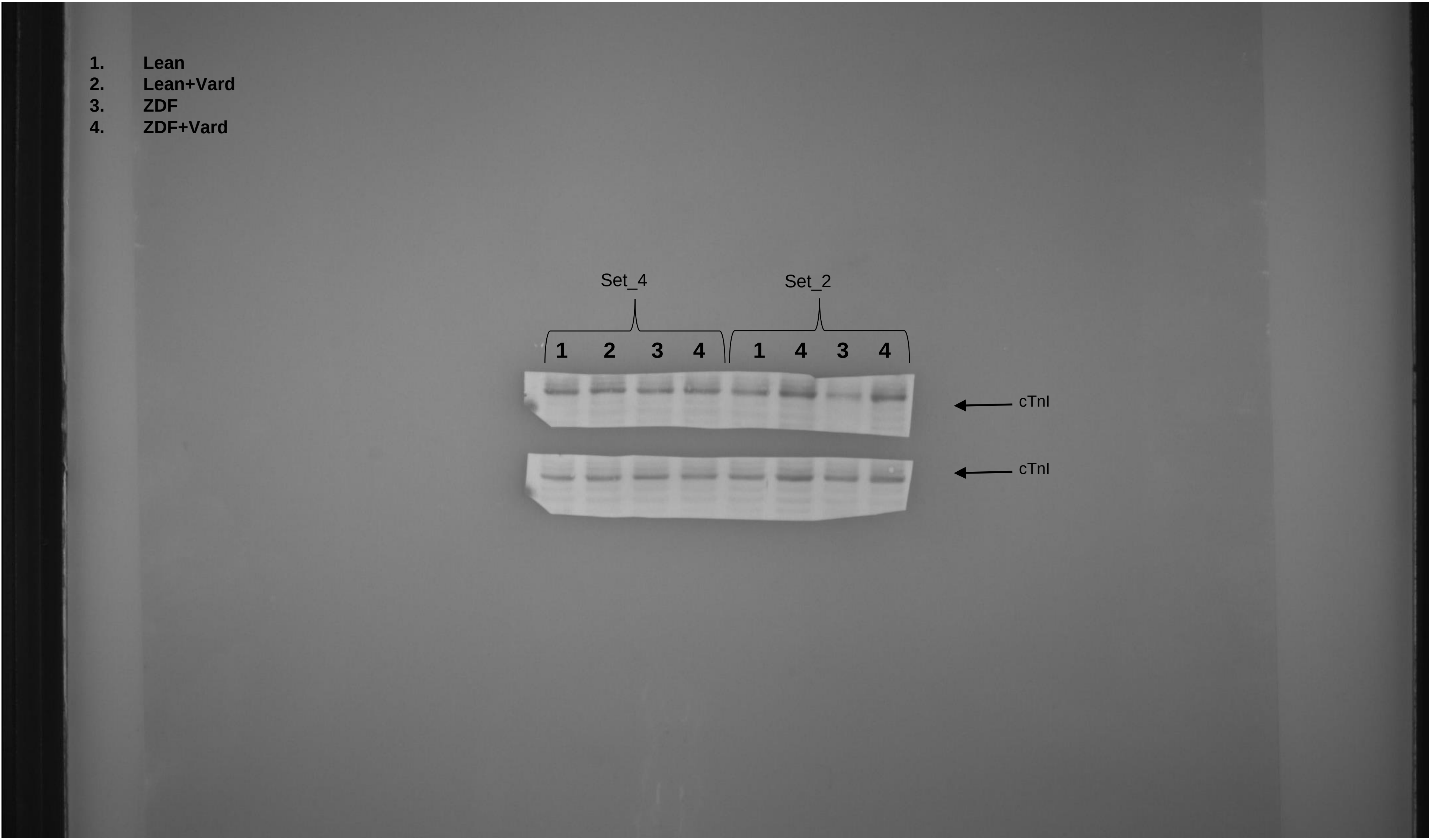

<u>**Right ventricle_Cardiac Troponin I_original membranes_ Thr-144-P**</u>

1. Lean
2. Lean+Vard
3. ZDF
4. ZDF+Vard

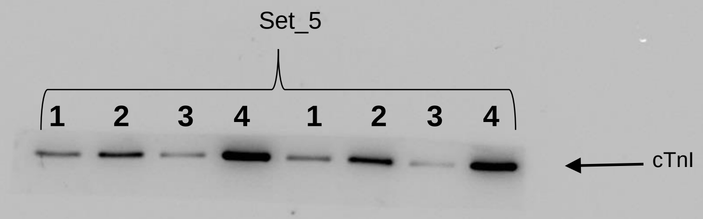

Right ventricle_Cardiac Troponin I_original membranes_ Thr-144-P-Blot stain

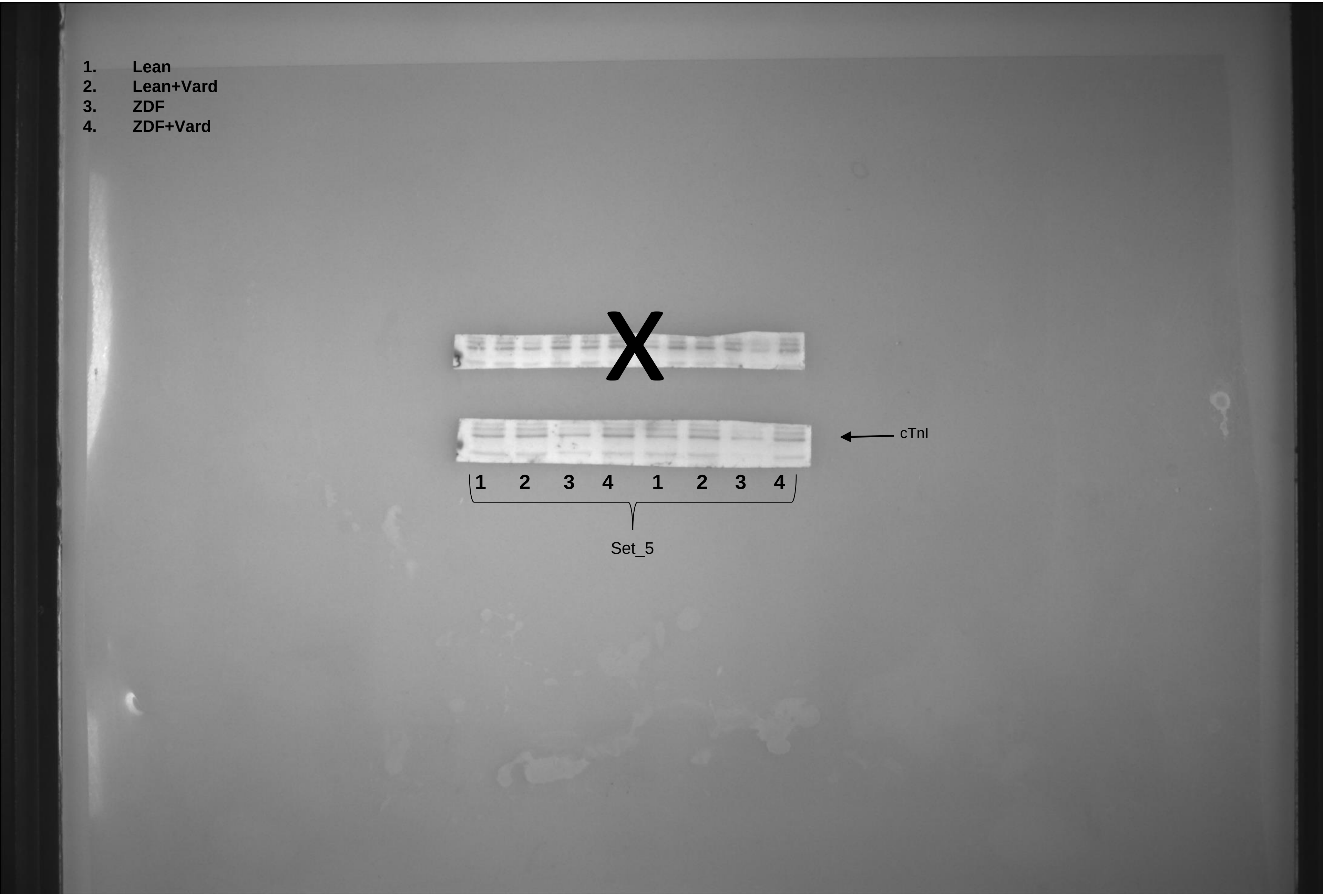

Right ventricle_Cardiac Troponin I_original membranes_ Thr-144-P

1. Lean
2. Lean+Vard
3. ZDF
4. ZDF+Vard

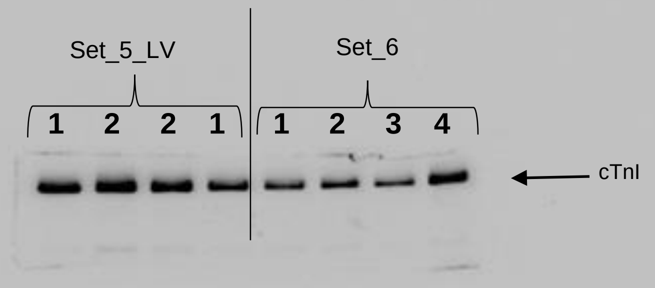

Right ventricle_Cardiac Troponin I_original membranes_ Thr-144-P-Blot stain

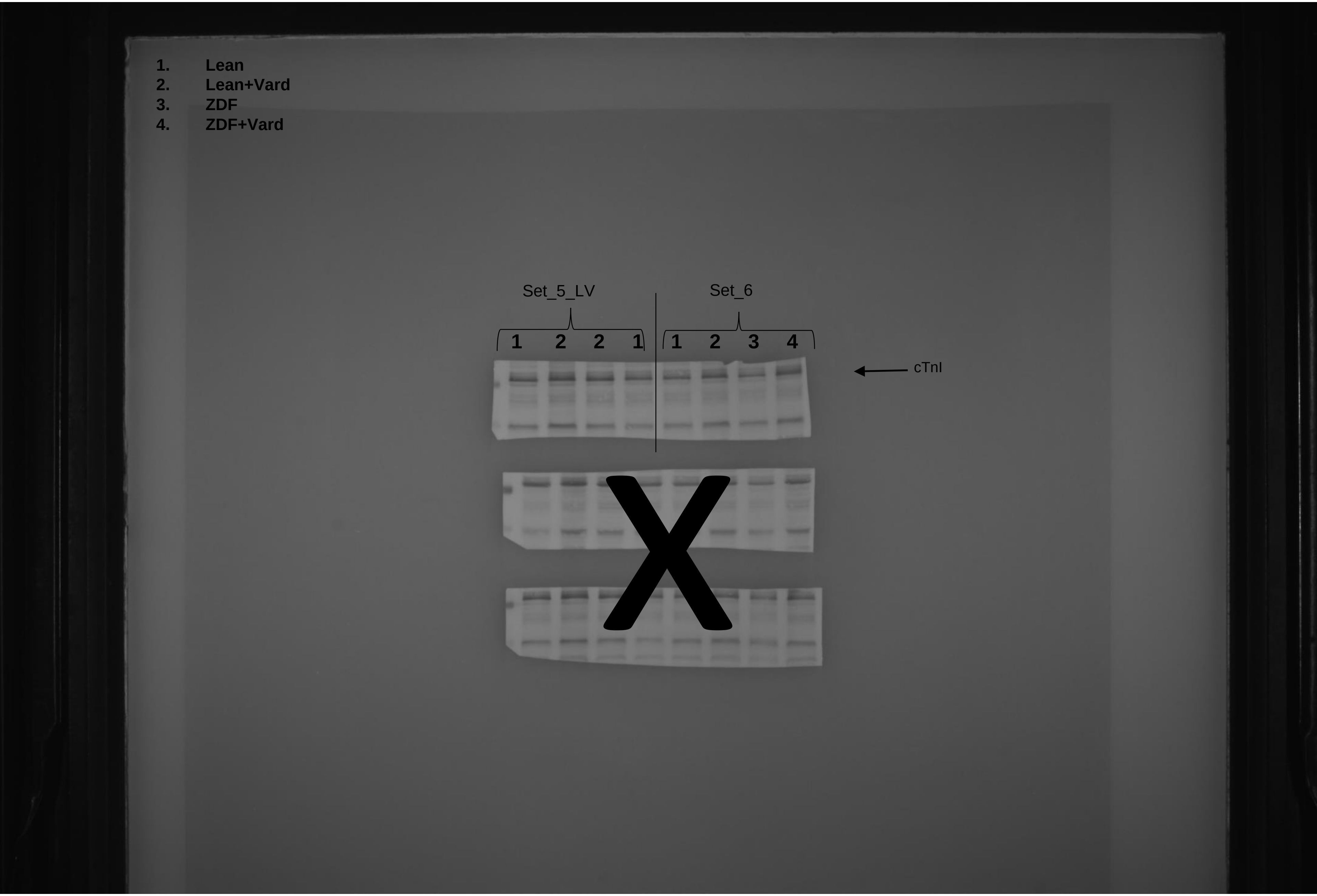

Right ventricle_Cardiac Troponin I_original membranes_ Thr-144-P

1. Lean
2. Lean+Vard
3. ZDF
4. ZDF+Vard

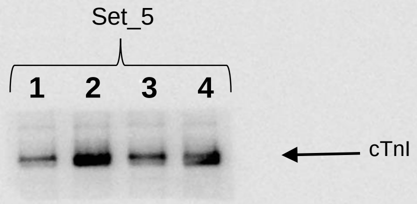

Right ventricle_Cardiac Troponin I_original membranes_ Thr-144-P-Blot stain

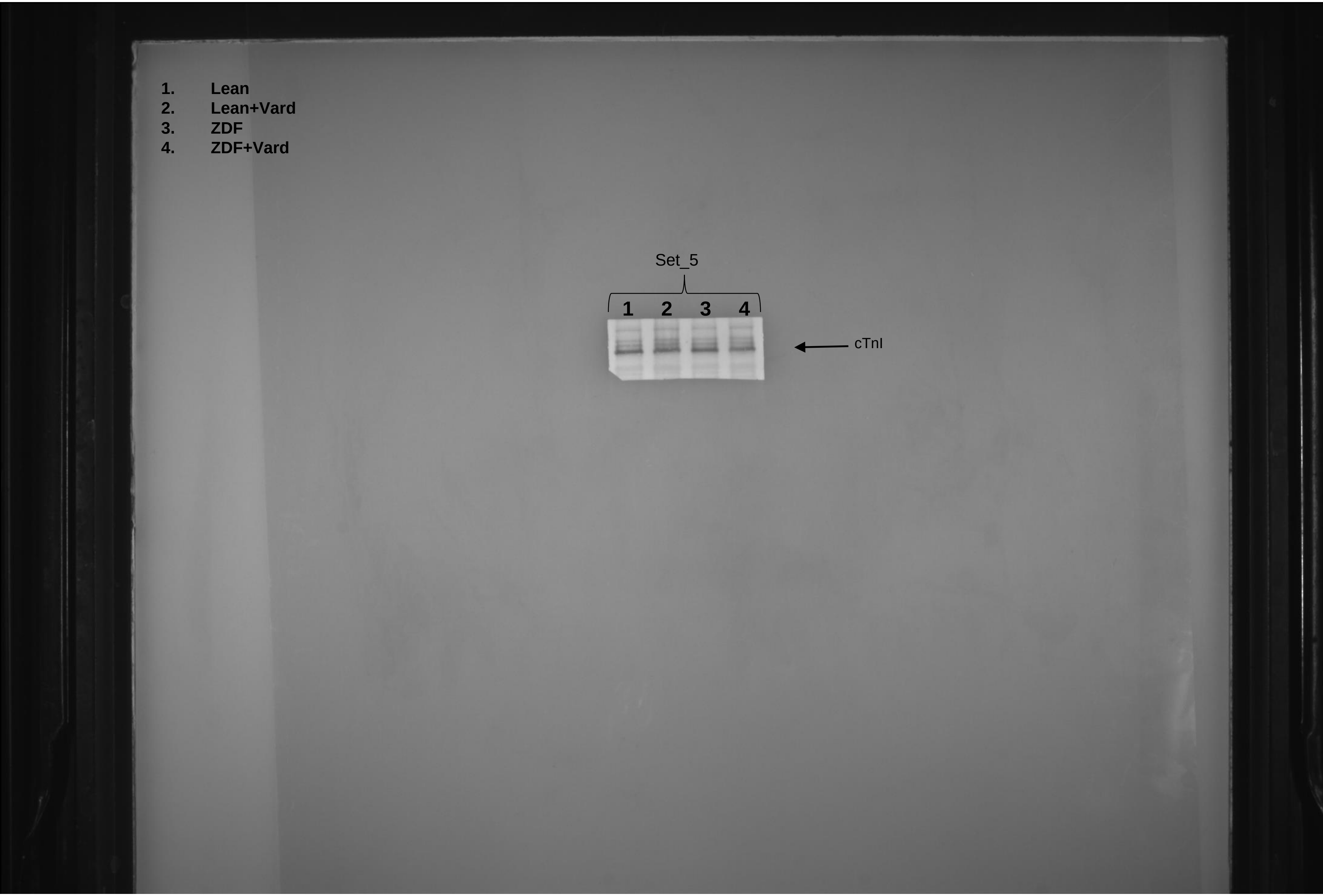

<u>**Right ventricle_Cardiac myosin binding protein C_original membranes_ Ser-282-P**</u>

1. Lean
2. Lean+Vard
3. ZDF
4. ZDF+Vard

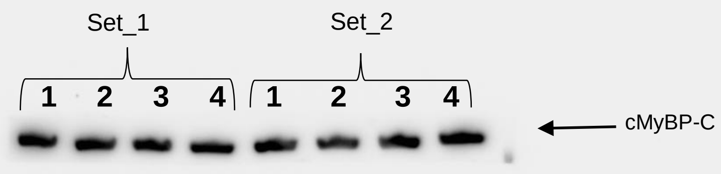

Right ventricle_Cardiac myosin binding protein C_original membranes_ Ser-282--Blot stain

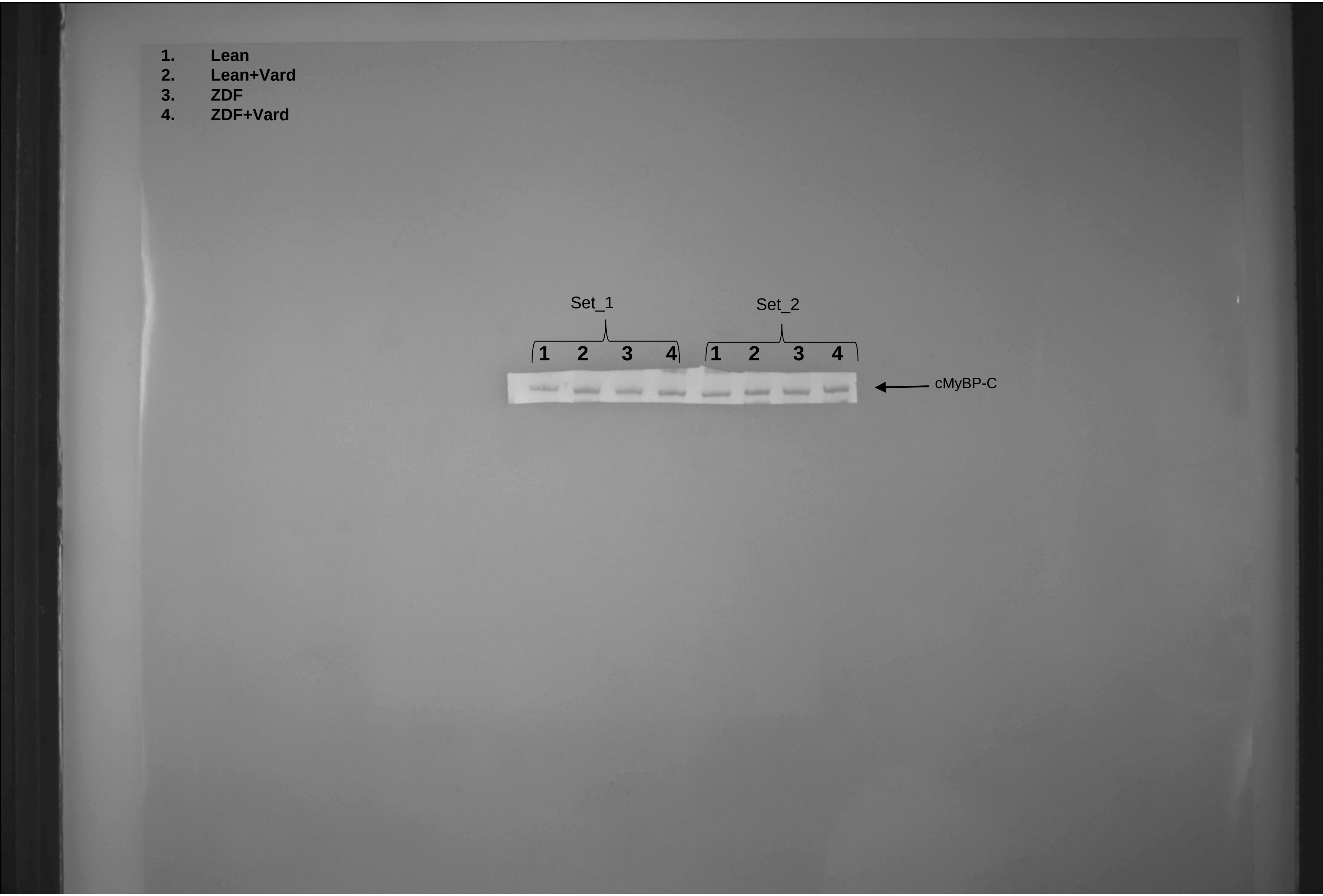

Right ventricle_Cardiac myosin binding protein C_original membranes_ Ser-282-P

1. Lean
2. Lean+Vard
3. ZDF
4. ZDF+Vard

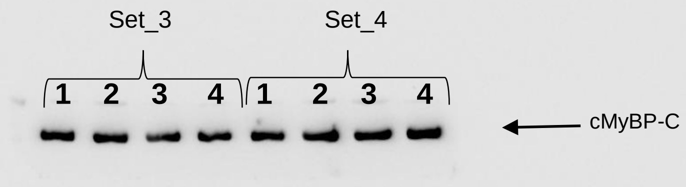

Right ventricle_Cardiac myosin binding protein C_original membranes_ Ser-282-P-Blot stain

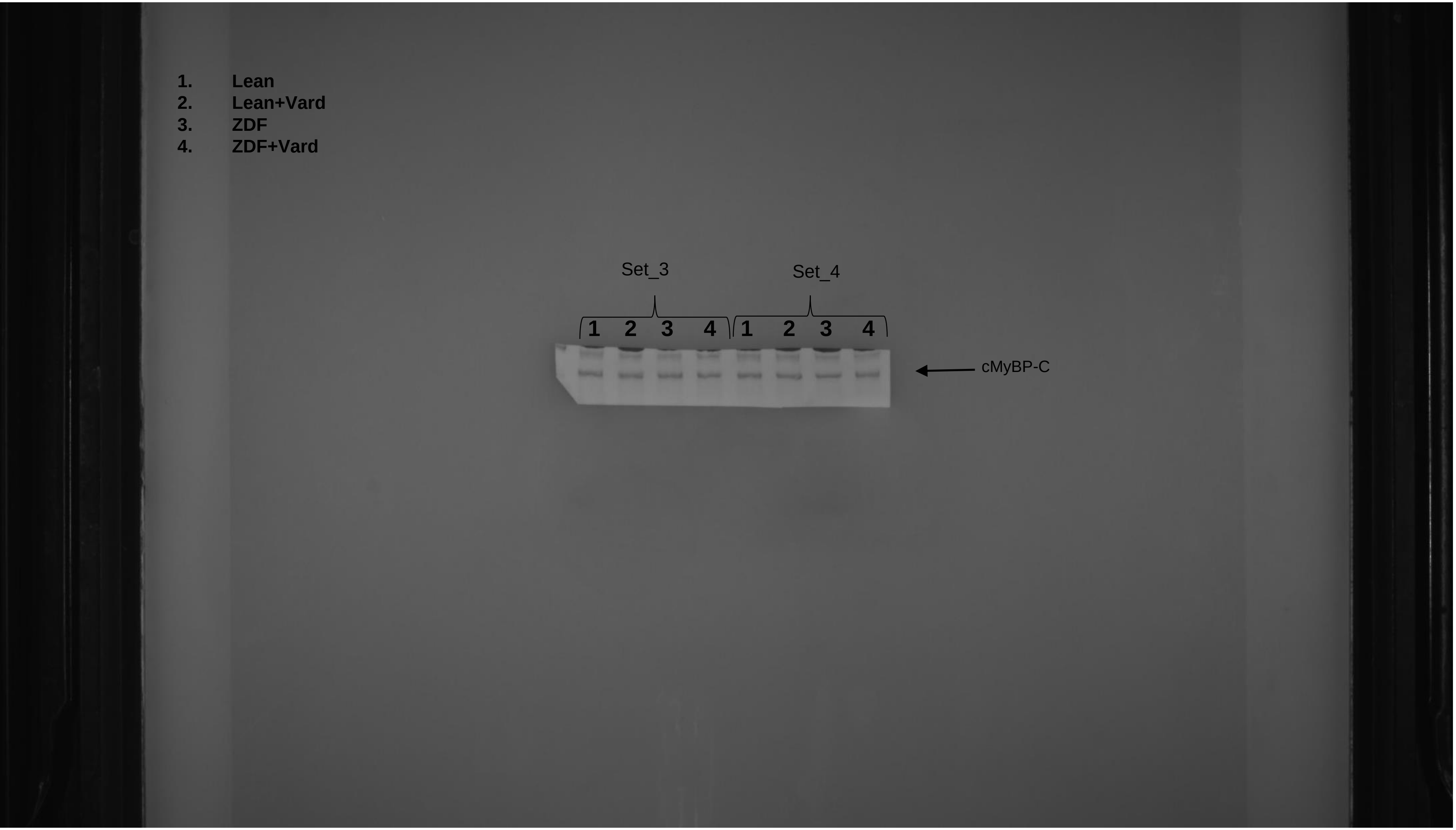

Right ventricle_Cardiac myosin binding protein C_original membranes_ Ser-282-P

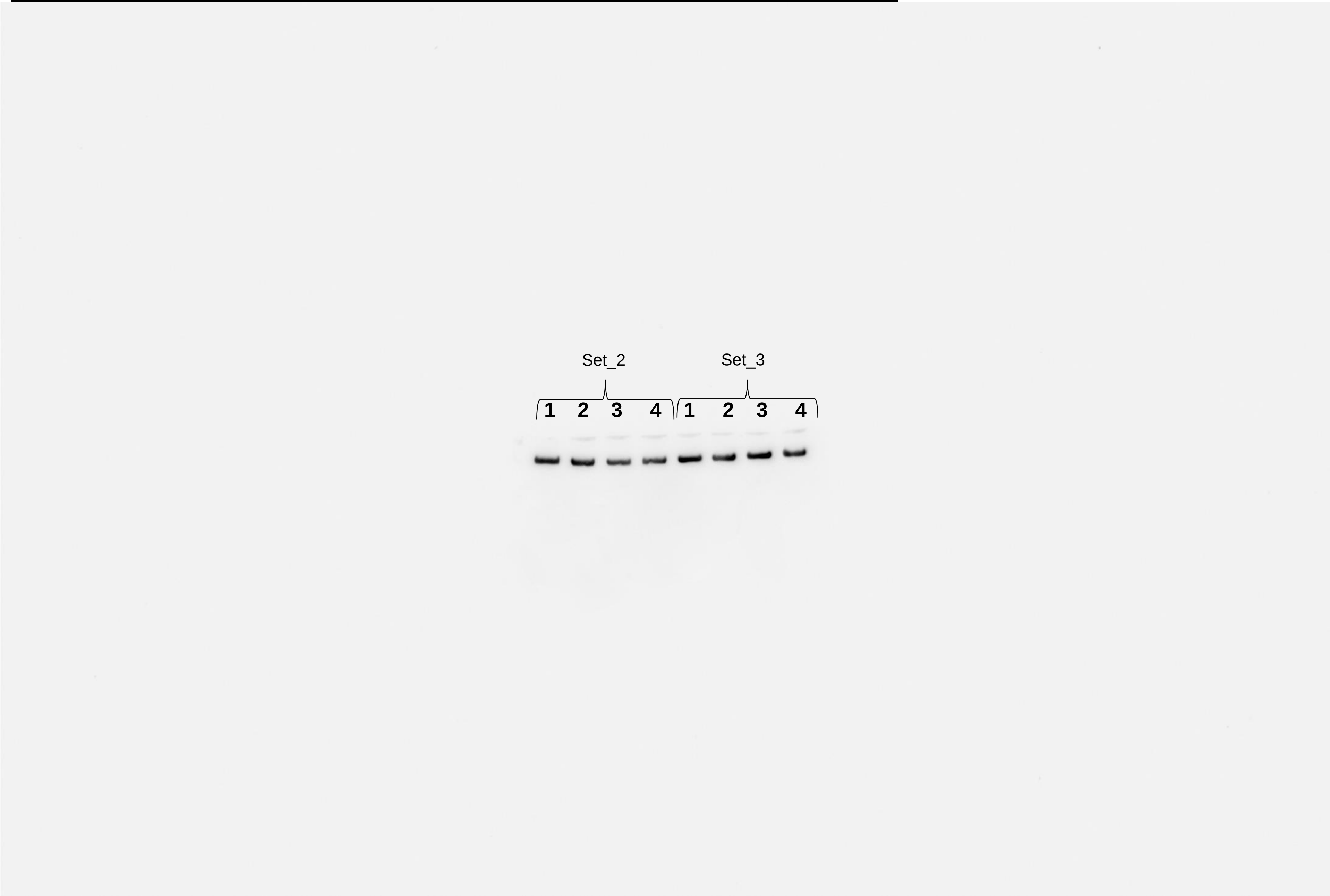

Right ventricle_Cardiac myosin binding protein C_original membranes_ Ser-282-P-Blot stain

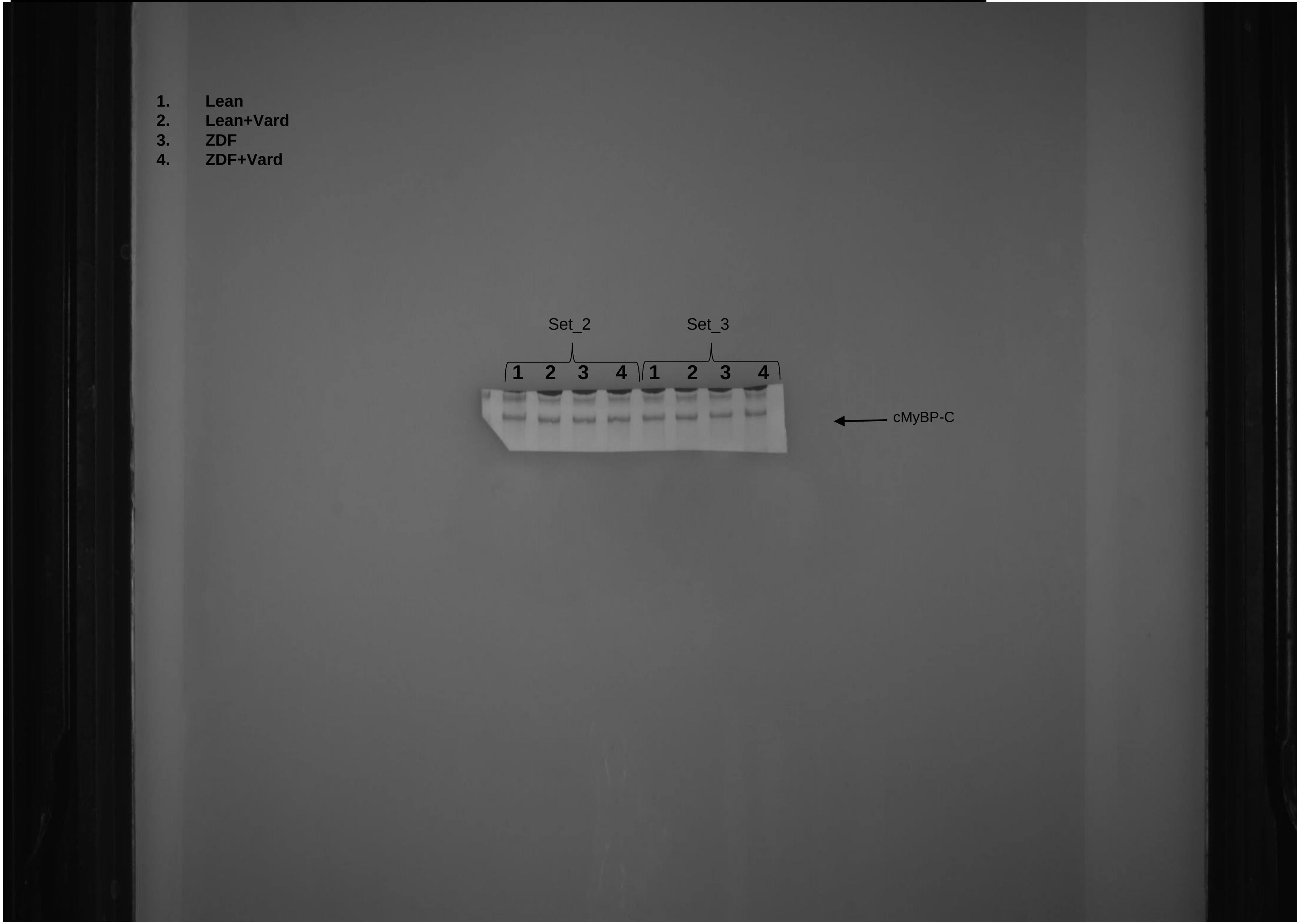

Right ventricle_Cardiac myosin binding protein C_original membranes_ Ser-282-P

1. Lean
2. Lean+Vard
3. ZDF
4. ZDF+Vard

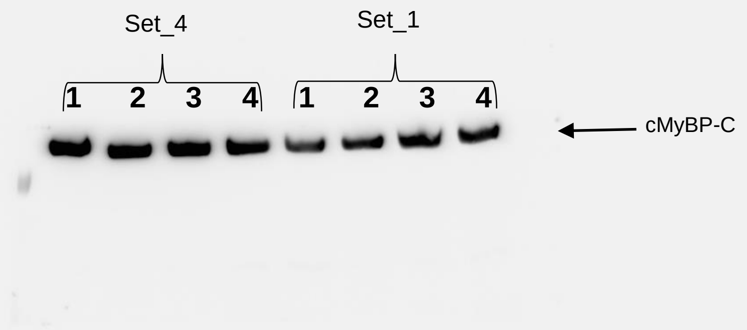

Right ventricle_Cardiac myosin binding protein C_original membranes_ Ser-282-P-Blot stain

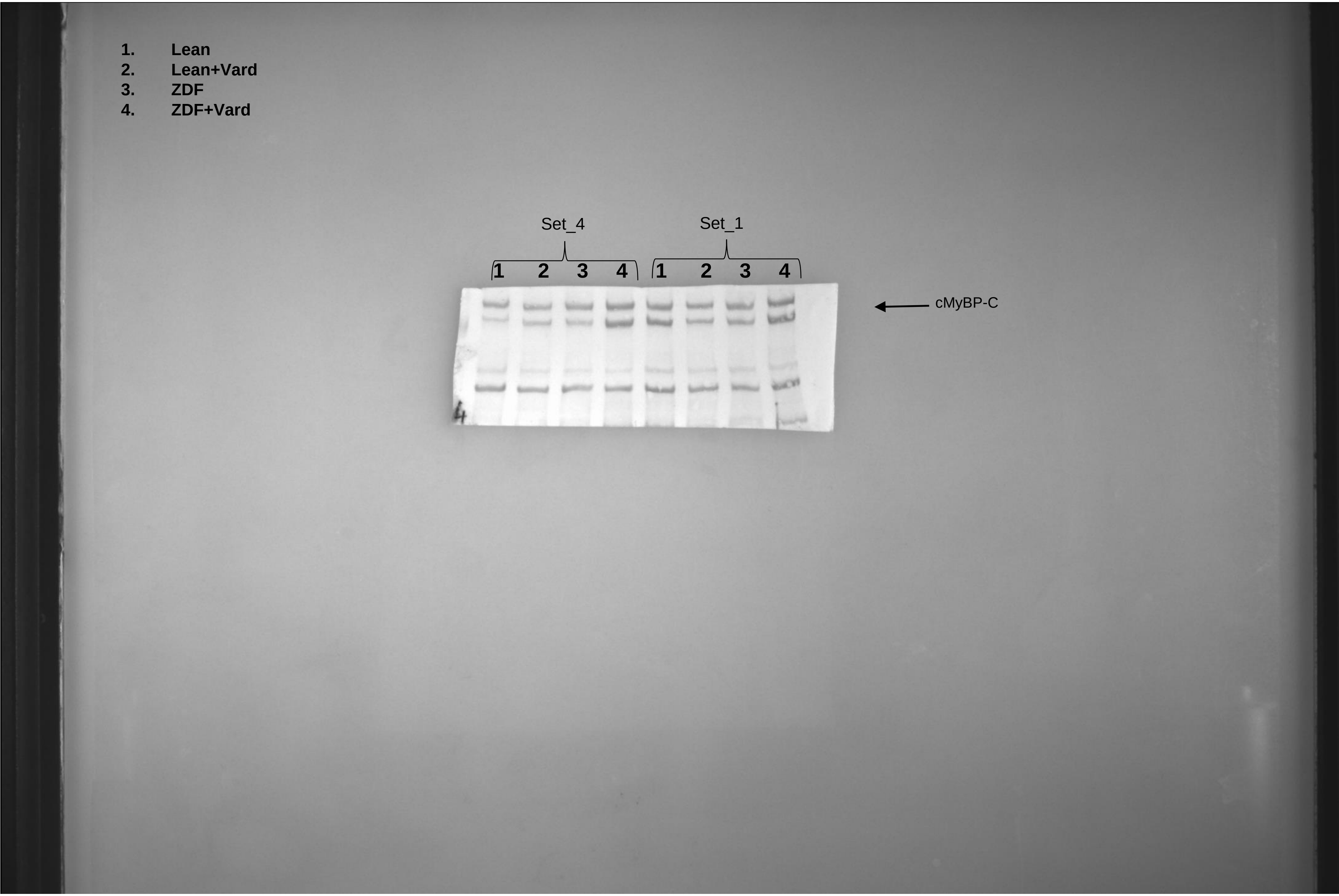

Right ventricle_Titin-P_original gel

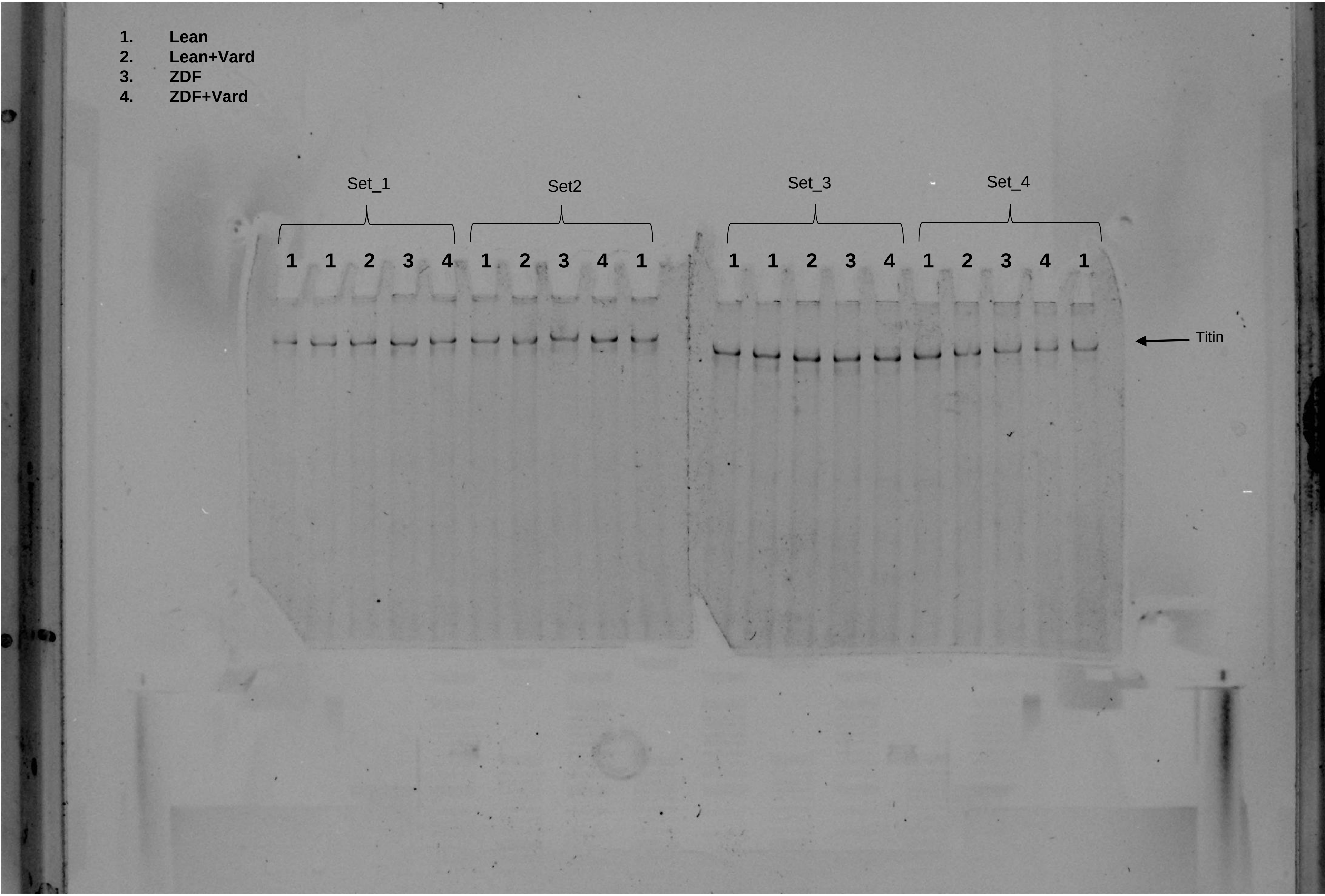

Right ventricle_Titin-total protein_original gel

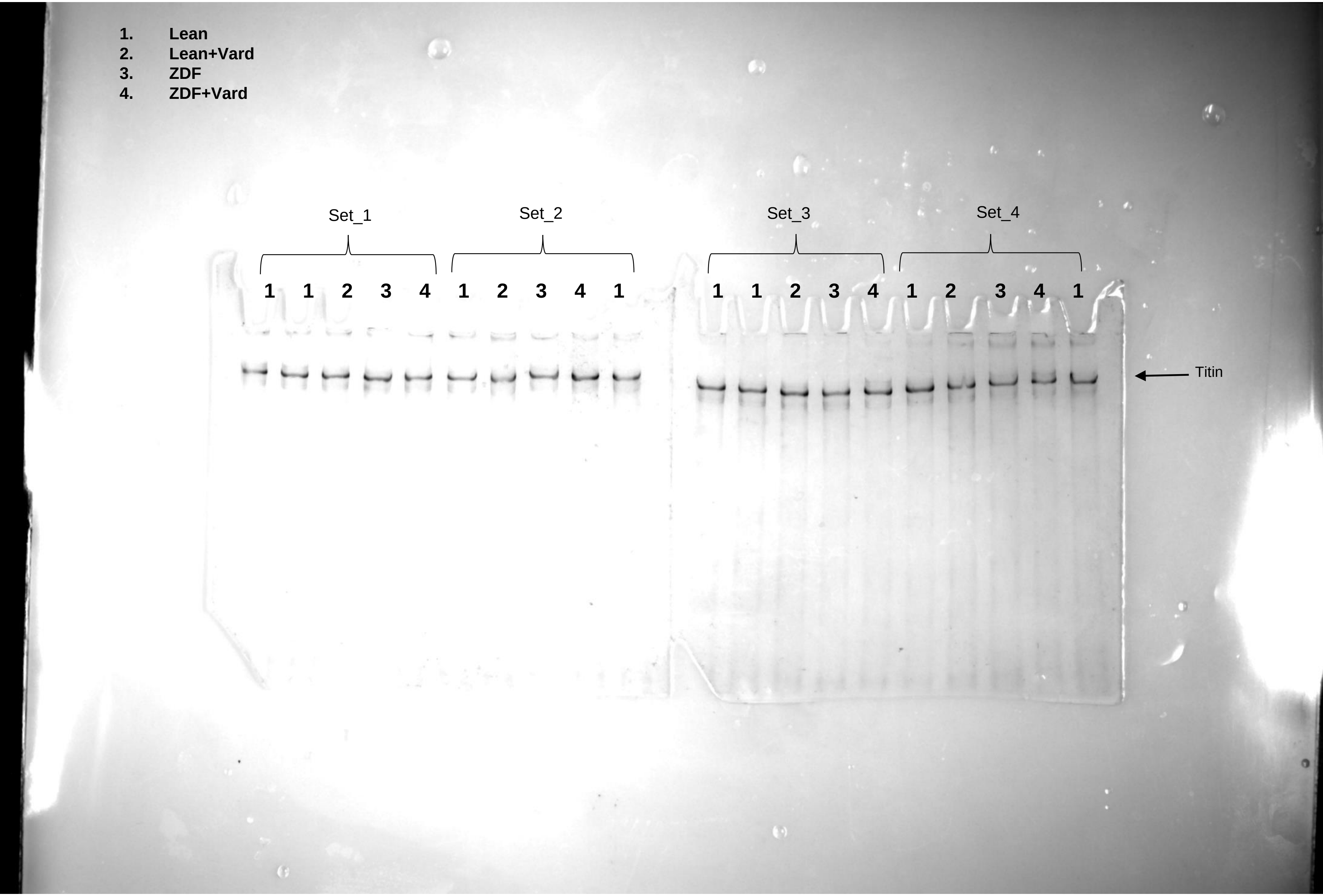

Right ventricle_Titin-P_original gel

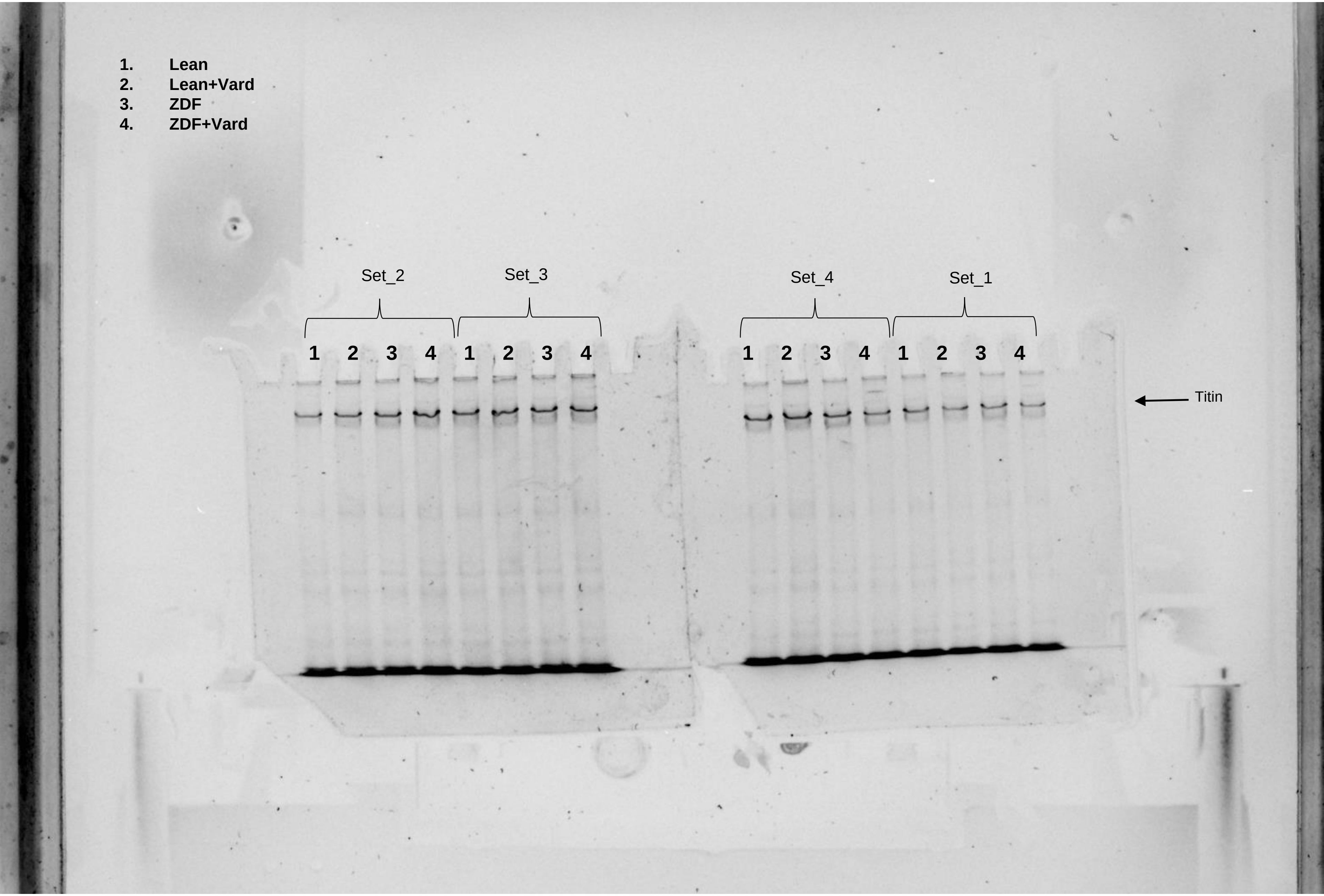

Right ventricle_Titin-total protein_original gel

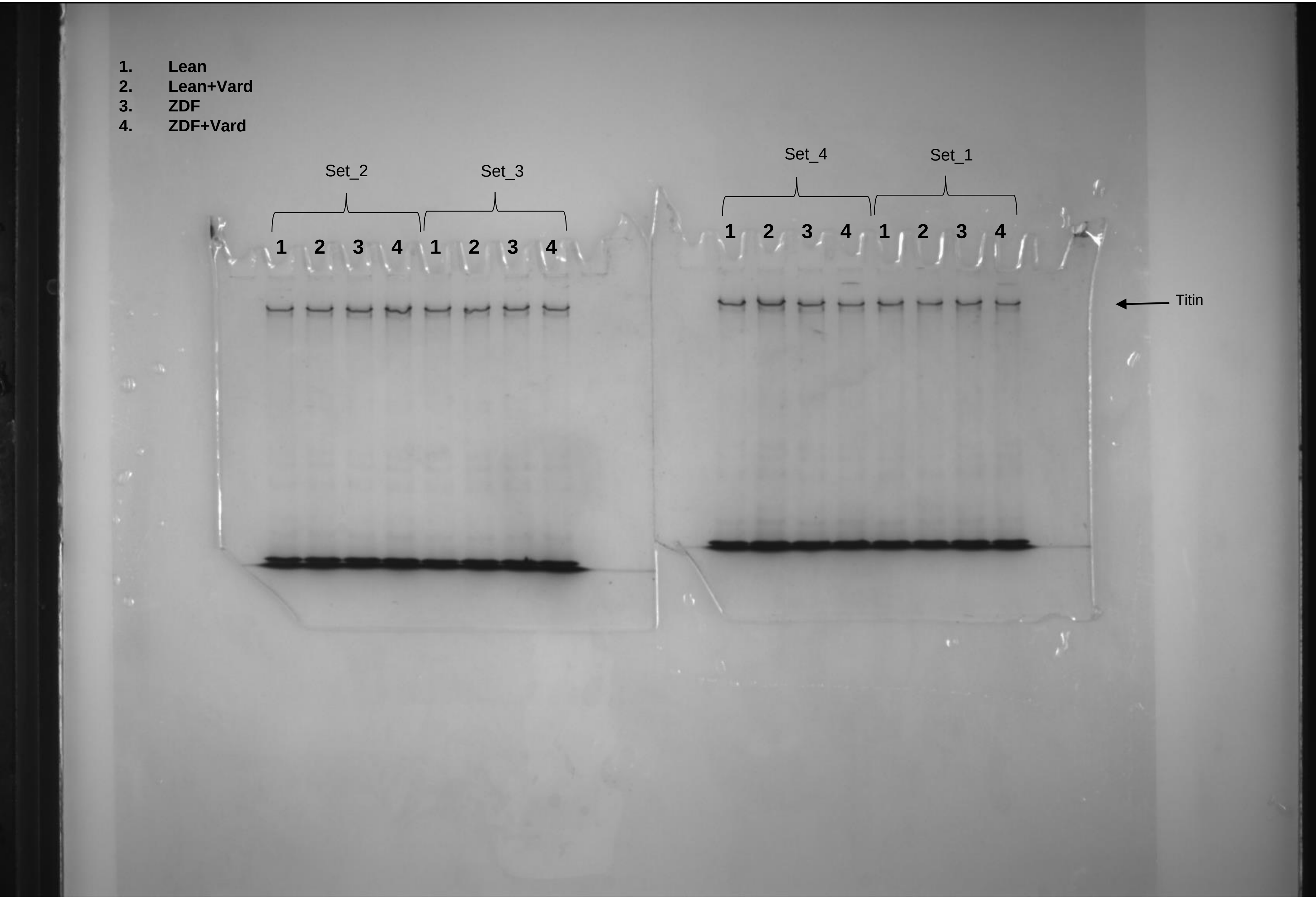

Supplementary Table S1.

	LEFT VENTRICLE								RIGHT VENTRICLE							
	Number of heart samples (n)				Number of wells				Number of heart samples (n)				Number of wells			
	Lean	Lean +Vard	ZDF	ZDF +Vard	Lean	Lean +Vard	ZDF	ZDF +Vard	Lean	Lean +Vard	ZDF	ZDF +Vard	Lean	Lean +Vard	ZDF	ZDF +Vard
**cTnI$^{\text{Ser-22/23-P}}$**	4	4	4	4	13	12	13	11	4	4	4	4	13	11	11	12
**cTnI$^{\text{Ser-43-P}}$**	5	5	5	5	12	14	13	15	4	4	4	4	10	9	11	11
**cTnI$^{\text{Thr-144-P}}$**	5	5	5	5	15	13	12	12	6	5	6	6	14	12	12	15
**cMyBP-C$^{\text{Ser-282-P}}$**	5	5	5	5	10	10	10	10	4	4	4	4	8	8	8	8
**Titin-P**	5	5	5	5	18	12	12	12	4	4	4	4	12	8	8	8
